# A group randomized trial using an appointment system to improve adherence to ART at reproductive and child health clinics implementing Option B+ in Tanzania

**DOI:** 10.1371/journal.pone.0184591

**Published:** 2017-09-28

**Authors:** Dennis Ross-Degnan, John Chalker, Jafary Liana, Mwikemo Deborah Kajoka, Richard Valimba, Suleiman Kimatta, Angel Dillip, Catherine Vialle-Valentin, Martha Embrey, Rachel Lieber, Keith Johnson

**Affiliations:** 1 Harvard Medical School, Boston, Massachusetts, United States of America; 2 Harvard Pilgrim Health Care Institute, Boston, Massachusetts, United States of America; 3 Pharmaceuticals and Health Technologies Group, Management Sciences for Health, Arlington, Virginia, United States of America; 4 Apotheker Consultancy Ltd, Dar es Salaam, Tanzania; 5 Ministry of Health, Community Development, Gender, Elderly and Children, Dar es Salaam, Tanzania; 6 Management Sciences for Health, Dar es Salaam, Tanzania; 7 Ifakara Health Institute, Dar es Salaam, Tanzania; Azienda Ospedaliera Universitaria di Perugia, ITALY

## Abstract

**Introduction:**

In October 2013, Tanzania adopted Option B+ under which HIV-positive pregnant women are initiated on antiretroviral therapy in reproductive and child health clinics at diagnosis. Studies have shown that adherence and retention to antiretroviral treatment can be problematic.

**Methods:**

We implemented a group randomized controlled trial in 24 reproductive and child health clinics in eight districts in Mbeya region. The trial tested the impact of implementing paper-based appointment tracking and community outreach systems on the rate of missed appointments and number of days covered by dispensed antiretroviral medications among women previously established on antiretroviral therapy. We used interrupted time series analysis to assess study outcomes. Clinic staff and patients in intervention clinics were aware of the intervention because of change in clinic procedures; data collectors knew the study group assignment.

**Results:**

Three months pre-intervention, we identified 1924 and 1226 patients established on antiretroviral therapy for six months or more in intervention and control clinics, respectively, of whom 83.4% and 86.9% had one or more post-intervention visits. The unadjusted rate of missed visits declined from 36.5% to 34.4% in intervention clinics and increased from 38.9% to 45.5% in control clinics following the intervention. Interrupted time series analyses demonstrated a net decrease of 13.7% (95% CI [-15.4,-12.1]) for missed visits at six months post-intervention. Similar differential changes were observed for visits missed by 3, 7, 15, or 60 days.

**Conclusion:**

Appointment-tracking and community outreach significantly improved appointment-keeping for women on antiretroviral therapy. The facility staff controlled their workload better, identified missing patients rapidly, and worked with existing community organizations. There is now enough evidence to scale up this approach to all antiretroviral therapy and Option B+ reproductive and child health clinics in Tanzania as well as to evaluate the intervention in medical clinics that treat other chronic health conditions.

**Trial registration:**

Registry for International Development Impact Evaluations ID-55310280d8757

## Introduction

Until recently, all antiretroviral therapy (ART) for HIV and AIDS in Tanzania was administered through specialized clinics. In October 2013, Tanzania implemented a new policy calling for Option B+ treatment the for pregnant women in reproductive and child health (RCH) clinics in 10 regions with a high HIV burden, as previously recommended by World Health Organization (WHO) guidelines published in 2010 [1]. Under Option B+, women who test positive for HIV during pregnancy are initiated on lifelong ART regardless of CD4 count. In Tanzania, RCH clinics now provide ART to women both prenatally and for two postnatal years.

Previous studies in Africa have noted that pregnant women initiated on ART at the time of diagnosis have low rates of adherence to ART and treatment retention, with up to 33% of women discontinuing care within a year [2–7]. Important barriers to retention in care have included stigma and denial of HIV status [2,3], lack of partner support [8], lack of funds for transport [2], starting ART at a younger age [4–9], negative interactions with health workers [3], and facility-based factors such as drug shortages, lack of integration between HIV and non-HIV services, inconvenient operating hours, and long waiting times [10–12]. Researchers have hypothesized that improvements in clinic processes and active tracking of patients after missed visits might help to address these issues [2,7,8,13]. However, the only systematic review of interventions to improve retention in care among pregnant and breastfeeding women concluded that the evidence base is weak overall [14].

We previously showed that attending appointments on time in ART clinics in East Africa was associated with medication adherence and subsequent clinical outcomes [15]. Until recently, few ART clinics in Tanzania could rapidly identify patients who missed visits [16]. Following these studies, the Tanzanian Ministry of Health and Social Welfare (MOHSW) (now known as Ministry of Health, Community Development, Gender, Elderly and Children) distributed the appointment books to all HIV clinical treatment centres and RCH clinics, but staff in the RCH clinics were not trained in their use, and they did not implement the system. The specific objective of the current study was to assess the impact of a managed introduction of this appointment and community-based tracking system in Option B+ RCH clinics on appointment-keeping and medication adherence.

## Materials and methods

### Study design and sample

We implemented a group randomized controlled trial in 24 Option B+ clinics in Mbeya region of Tanzania, which the MOHSW had identified as a priority because of its high prevalence of HIV-positive pregnant women. To reduce cross-contamination, we first paired districts in the region by their numbers of Option B+ clinics having 70 or more women on ART at the time of baseline data collection. We then randomly assigned one district in each pair to the intervention group, which resulted in a total of 12 intervention clinics in four districts and 12 control clinics in four separate districts.

Our study group comprised a fixed cohort of women who had received ART for six or more months and who had made at least one clinic visit three to six months before the intervention (“established” patients). We hypothesized that a focus on appointment-keeping would prevent declining adherence over time in these established patients, as observed in our previous study [17]. No women who met the visit attendance criteria were excluded, although we limited the sample to 200 established patients per clinic. Data were collected on clinic visits from 12 months before to five months after the end of the four-month intervention. Clinic records did not consistently capture reasons for dropout, but we compared key demographic and clinical characteristics of dropouts in intervention and control clinics over time to assess differential dropout.

### Description of the intervention

The intervention consisted of a training of trainers, followed by two clinic staff trainings and four rounds of supportive supervision. The trainers’ training prepared a team to train and supervise clinic staff. The subsequent two-day interactive training sessions introduced Option B+ clinic staff to the skills and systems needed to quickly identify patients who missed clinic appointments and initiate early community-based follow-up to encourage them to return to care. Training participants included two nurses from the Option B+ clinic, one from the ART clinic, and the RCH clinic staff person in-charge from each of the 12 intervention sites, as well as the district RCH coordinators. The training covered: (1) adherence, retention, and the baseline findings in each clinic; (2) introduction of a standardized paper-based appointment register; (3) use of another register to track patients who miss appointments; and (4) calculation of monthly percentages of patients who attend appointments on time to monitor progress.

This training was followed by four monthly supportive supervision visits in each intervention clinic by trainers and MOHSW staff. During each visit, supervisors reviewed appointment books and reinforced the processes for scheduling appointments, recording attendance, following up patients who missed appointments, and calculating monthly progress indicators.

The control clinics received no intervention other than the MOHSW’s previous distribution of appointment and patient-tracking registers.

### Outcomes and data sources

Our primary outcome is the rate of clinic visits missed on the scheduled day, measured using routine data collected from the standard revised clinic treatment card (CTC-2) used in all Tanzanian ART clinics. We also assessed the rates of visits missed by 3, 7, 15 and 60 days or more to measure success in reducing longer gaps in care. In addition, using data on days of medication dispensed, we measured the average proportion of days covered (PDC) by dispensed medications per month, as well as the proportions of patients with more than 80% PDC and more than 95% PDC.

In May 2015, two months before the intervention, we conducted a baseline assessment to collect retrospective data on clinic attendance and dispensing from June 2014 through April 2015, which informed each clinic’s intervention. In another assessment five months after the final supervision visit, we collected retrospective data from April 2015 through April 2016. During the assessments, data collectors identified women in the study population and recorded data on all their clinic visits, including visit date, next scheduled visit date, and medicines dispensed. We also collected data on age, marital status, year of ART initiation, WHO clinical stage at initiation, and month of delivery.

Data were collected on tablet computers with an Open Data Kit application and uploaded daily to a web-based aggregator. Supervisors checked and resolved problems with data collectors each evening after they returned from the clinics.

### Sample size

The baseline rate of missing clinic appointments in ART clinics in our previous study was about 30%. In this context, with 12 clinics per study group, an average of 100 patients per clinic per month, and a planned 12 months before and nine months after the start of the intervention, using a standard difference-in-difference analysis approach, we expected 80% power (two-sided alpha = 0.05) to detect changes in rate of missed visits of 4.6% to 6.0%, assuming 0.001 and 0.01 intra-cluster correlation between rates of visits, respectively [18–20]. The power of interrupted time series with comparison analyses are typically greater than difference-in-difference analyses because they can account for differences in underlying trend in study groups [20].

### Data analysis

All statistical analyses were conducted with Stata V14 [21]. We first summarized patient characteristics and baseline outcome rates in our cohort of established patients in intervention and control clinics and compared the groups statistically using chi-square tests. We then summarized the unadjusted rates of missed visits and monthly PDC in the pre-intervention and post-intervention periods by individual clinic and by study group.

We next graphed monthly rates of all study outcomes. We used aggregate interrupted time series (ITS) models to compare pre-post changes in the rate of study outcomes among established ART patients. We defined the beginning of the post-intervention period as September 1, 2015, a month after the initial training of clinic staff ended, because the intervention could not affect patients until after they had presented for their next monthly visit. We considered the entire period after this as the post-intervention period. To determine relative effects in intervention versus control clinics, we calculated the differences in monthly rates (intervention minus control) and used ITS segmented regression models to estimate post-intervention changes in level and trend of these differences compared to baseline. To summarize the effect of the intervention, we used the Stata margins command to calculate the difference between intervention and control clinics at six months after the start of the intervention.

Our randomized design minimized the possibility that an external co-intervention could be responsible for observed effects. To examine whether differential dropout might explain the effects, we conducted two secondary analyses. First, we estimated intervention effects among established patients with a visit in 2016 near the end of follow-up, comparing these with the estimated effects in the overall study population. Second, we examined the monthly rates of key population characteristics among patients who visited intervention and control clinics over time to detect any discontinuities.

### Ethical approval

The National Institute for Medical Research in Tanzania approved the study. The trial tested a supportive intervention to implement an appointment and outreach system previously mandated by the Tanzania National AIDS Control Programme. The Tanzania National Institute for Medical Research determined that the study was a quality improvement intervention with minimal risk and required no subject consent. The Institutional Review Board of Harvard Pilgrim Health Care Institute determined that the analysis of de-identified data for the evaluation was exempt from human studies review.

## Results

**Fig 1:**
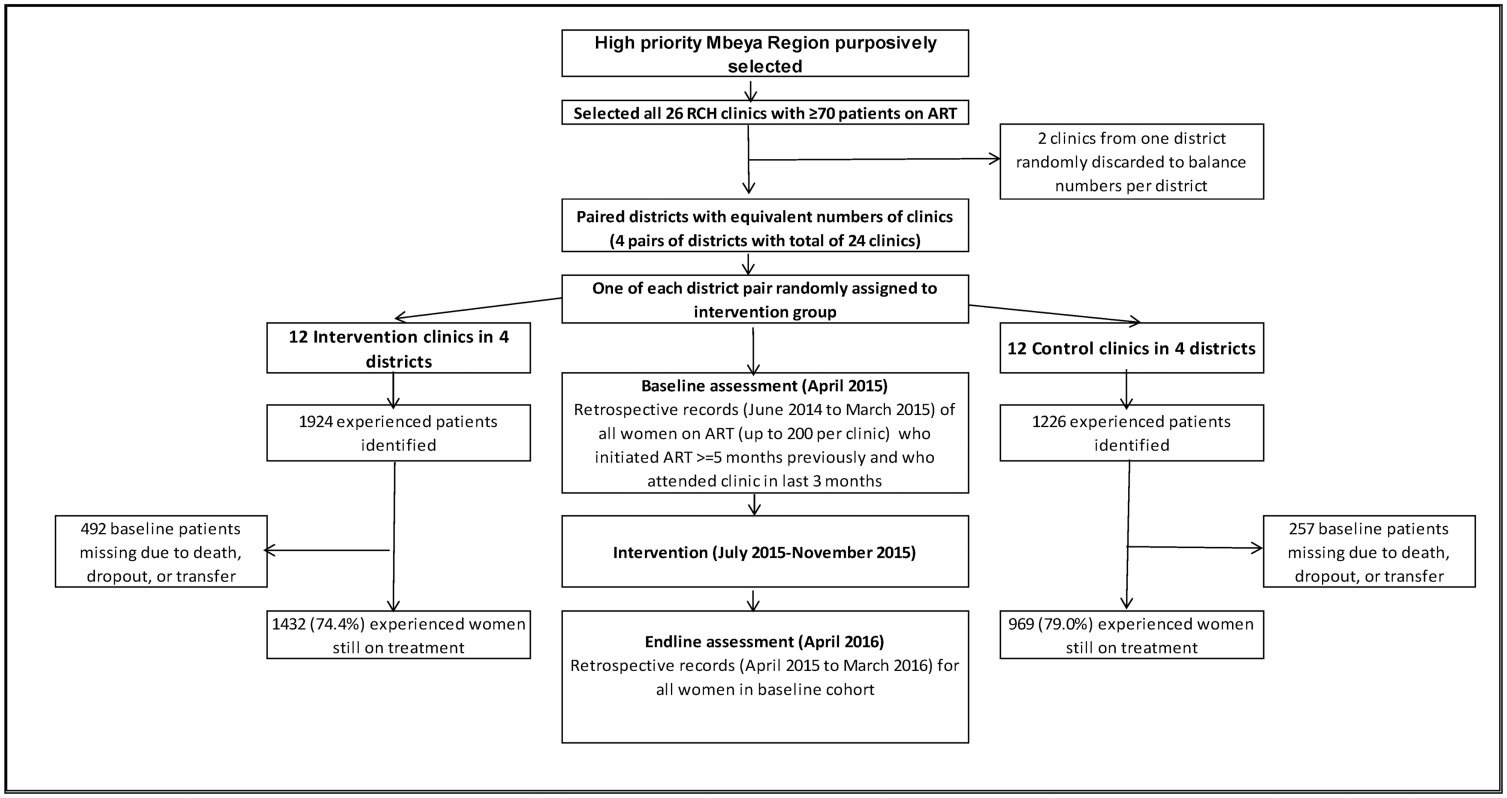
Participant selection and flow during the trial. The waterfall diagram presents sample sizes and exclusions in selecting clinics to participate in the study (top), and in selecting and following patients in intervention (left) and control (right) clinics following random assignment in relation to dates of assessments and the intervention (middle).

The clinic and study participant selection and flow are illustrated in Fig 1. We identified 1924 and 1226 established patients in intervention and control clinics, respectively, of whom 1433 (74.4%) and 970 (79.1%) were still on treatment in the same facility in 2016 (Table 1). Compared to controls, women in intervention clinics were somewhat younger (60.2% vs. 52.4% age 30 or less) and had initiated ART earlier (57.5% vs. 48.0% at WHO stage 1). Baseline rates of missing visits by seven or more days tended to be slightly higher in the intervention group and PDC slightly lower. However, women in the intervention and control groups exhibited few meaningful differences. Women who were no longer in treatment by the end of the study tended to be somewhat younger than those who remained in treatment, initiated ART earlier, and had higher baseline rates of missed visits (data not presented). These relationships were similar in the two study groups.

**Table 1:** Baseline characteristics and unadjusted outcomes among established patients included in the study.

	Control	Intervention	Total	
**Baseline N of patients**	**1,226**	**1,924**	**3,150**	
**Age**				**p<0.001**
<20yo	4.8%	8.2%	6.9%	
21-30yo	47.6%	52.0%	50.3%	
31-40yo	40.7%	35.8%	37.7%	
>40yo	3.5%	1.7%	2.4%	
Missing	3.3%	2.3%	2.7%	
**Marital Status**				**p<0.021**
Single	8.7%	9.1%	9.0%	
Married/cohabiting	62.2%	57.0%	59.0%	
Divorced/widowed	4.2%	4.3%	4.3%	
Missing	24.9%	29.7%	27.8%	
**WHO stage at treatment initiation**				**p<0.0001**
1	48.0%	57.5%	53.8%	
2	19.7%	12.9%	15.6%	
3	20.0%	15.8%	17.4%	
4	4.4%	2.0%	2.9%	
Missing	7.9%	11.9%	10.3%	
**Year of ART initiation**				**p = 0.11**
2012	4.7%	3.2%	3.8%	
2013	24.8%	26.9%	26.1%	
2014	67.7%	67.5%	67.6%	
2015	2.9%	2.4%	2.6%	
**Year of delivery**				**p = 0.80**
2013	9.3%	8.7%	8.9%	
2014	42.7%	42.2%	42.4%	
2015	24.7%	23.9%	24.2%	
2016	9.0%	9.7%	9.4%	
NA/missing	14.4%	15.5%	15.1%	
**Baseline N of visits**	**12,526**	**18,331**		
% visits missed	39.2%	37.0%		**p<0.001**
% missed by 3+ days	27.3%	27.1%		**p = 0.714**
% missed by 7+ days	17.3%	19.4%		**p<0.001**
% missed by 15+ days	12.5%	15.6%		**p<0.001**
% missed by 60+ days	3.3%	4.4%		**p<0.001**
**Baseline months of treatment**	**14,518**	**22,415**		
Avg proportion of days covered (PDC)	86.7%	83.3%		**p<0.001**
% with PDC 80% or more	81.4%	76.8%		**p<0.001**
% with PDC 95% or more	69.3%	63.8%		**p<0.001**

Note: P-values report the results of chi-square tests comparing baseline patients’ characteristics and baseline outcomes between intervention and control groups

Table in S1 Table summarizes the numbers of patients, visits, and unadjusted outcomes before and after the intervention by clinic and by study group. Overall, 1604 of 1924 (83.4%) women in the intervention group and 1065 of 1226 (86.9%) in the control group had one or more visits in the post-intervention period. The average number of visits during the entire study period was 14.9 for women in the intervention group and 15.6 for women in the control group.

Averaged across clinics, the rate of missed visits for women in the intervention group declined slightly from 36.5% to 34.4% after the start of intervention; for women in the control group, the rate of missed visits increased from 38.9% to 45.5% during the same period. A similar pattern of differential change between groups was observed for visits missed by 3, 7, 15, or 60 days. The average monthly percentage of days covered by medicines increased slightly in intervention clinics (83.4% vs. 85.7%) and decreased slightly in control clinics (86.6% vs. 85.2%). There were similar differences between groups in the percentages of women covering 80% or more and 95% or more of days with dispensed medicines.

### Changes in rates of monthly outcomes over time

**Fig 2:**
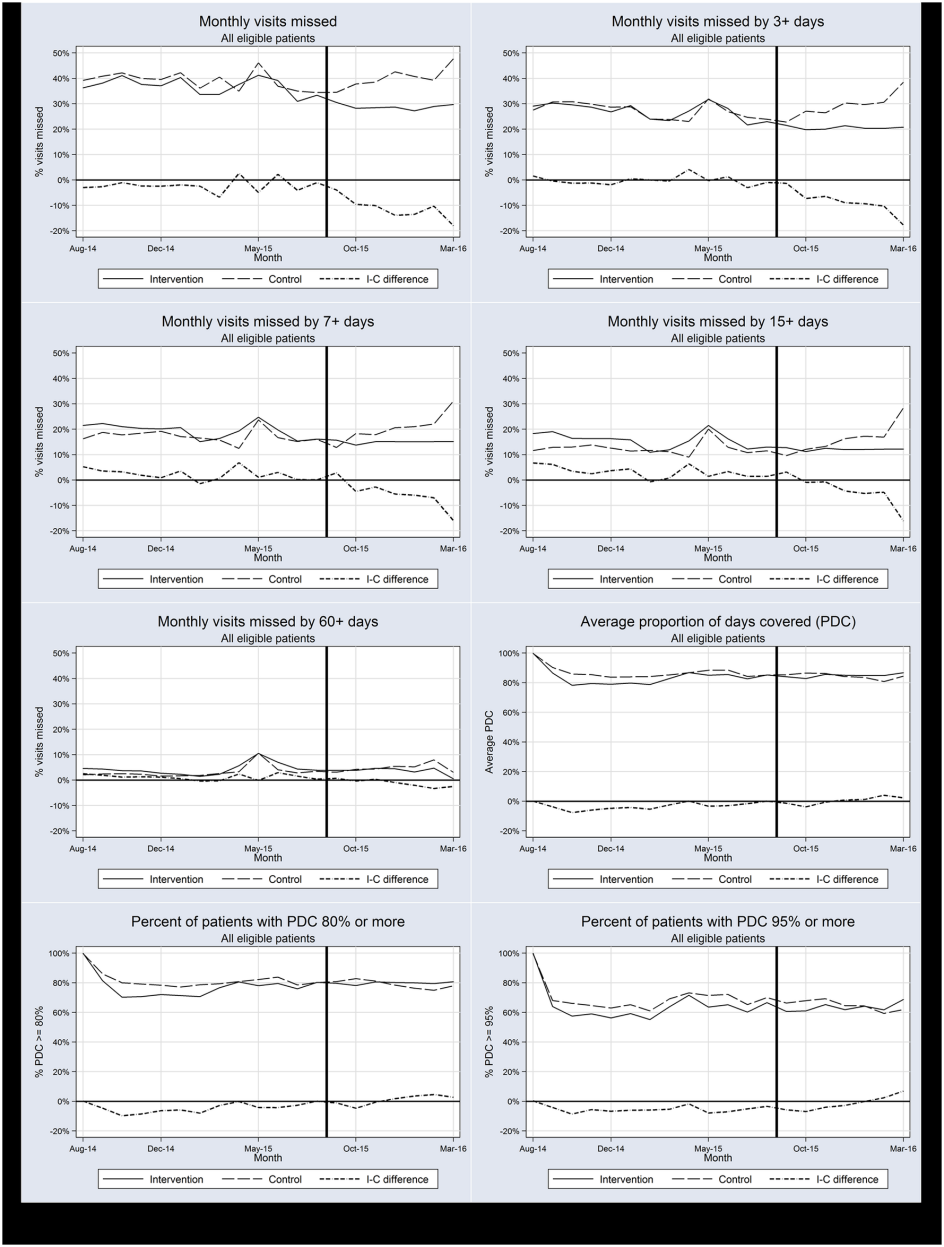
Time series of monthly study outcomes in intervention and control clinics and their differences over time among established patients. The figures present the monthly values of the eight study outcomes from August 2014 until March 2016 averaged across all clinics in the intervention (solid lines) and control (long dashed lines) groups, as well as the monthly differences between these values (short dashed lines). The intervention began in July 2015 (represented by the solid vertical lines) and continued with monthly supervisory visits in the following four months.

Fig 2 displays the time series comparing monthly rates of missed visits and PDC outcomes among women established on ART in intervention and control clinics before and after the start of the intervention, as well as the monthly differences in rates between intervention and control groups. During the baseline period, the groups were similar in both the level and trend of visits missed, as illustrated by monthly differences in rates that hover near zero percent. However, the monthly rates diverged after the start of the intervention, reaching differences of more than 10% between groups by six months after the intervention. The relative differences between groups following the intervention are much smaller for the rates of lost to follow-up, as measured by missing visits by more than 60 days.

For the three PDC study outcomes, post-intervention changes are less visible. The average PDC remains at slightly more than 80% for the entire study period following an initial decline from 100% that is intrinsic to the start of adherence measurement at a defined point in time. Intervention clinics had slightly lower average PDC than control clinics at baseline, and they averaged slightly higher than controls following the intervention. Similarly, the percentages of patients with PDC of 80% or more and of 95% or more in intervention clinics remained below those of control clinics at baseline, but increased to surpass controls during the post-intervention period.

Table 2 presents results of ITS segmented regression analyses of the monthly differences between intervention and control groups for established patients, expressed as relative changes in study outcomes at six months following the intervention; all models control for baseline differences in trend between groups. All models of missed visits identify significant relative declines in trend in intervention clinics following the intervention. The net estimated differences in outcomes in intervention versus control clinics at six months post-intervention are -13.7% (95% CI [-15.4,-12.1] for missing visits by 1 day or more and -12.5% [-14.7,-10.4%], -9.8% [-11.7,-7.8%], -8.7% [-11.1,-6.4%], and -3.3% [-4.8,-1.8%] for visit gaps of 3, 7, 15, and 60 days or more, respectively. Corresponding to the improved rates of appointment-keeping and the more regular supply of medications, the PDC ITS models identify a small nonsignificant increase in trend of average monthly PDC (+0.1% [-1.1, 1.4%]) and in the percentage of patients with PDC of 80% or more (+0.7% [-1.2, 2.6%]) and a significant increase in the percentage of patients with PDC of 95% or more (+6.6% [4.7, 8.4%]).

**Table 2:** Results of aggregate interrupted time series models predicting post-intervention changes in level and trend in the monthly differences between intervention (n = 1924)^&^ and control (n = 1226)^&^ groups, and estimated differences at six months post-intervention.

	Missed Visits*	Missed by 3+ days*	Missed by 7+ days*	Missed by 15+ days*	Missed by 60+ days	Average PDC	% with PDC ≥ 80	% with PDC ≥ 95*
**All Baseline Patients**								
Constant	-0.028***	0.0	0.038***	0.053***	0.016**	-0.072***	-0.095***	-0.067***
	[-0.03,-0.02]	[-0.01,-0.01]	[0.03,0.05]	[0.04,0.07]	[0.01,0.03]	[-0.08,-0.06]	[-0.11,-0.08]	[-0.08,-0.06]
Baseline trend	0.001*	0.0	-0.002**	-0.003**	-0.001	0.005***	0.006***	0.001*
	[0.00,0.00]	[-0.00,0.00]	[-0.00,0.00]	[-0.00,0.00]	[-0.00,0.00]	[0.00,0.01]	[0.00,0.01]	[0.00,0.00]
Level change post	-0.028***	0.0	0.030**	0.047**	0.01	-0.029***	-0.030**	-0.055***
	[-0.05,-0.01]	[-0.02,0.02]	[0.01,0.05]	[0.02,0.07]	[-0.00,0.02]	[-0.04,-0.02]	[-0.05.-0.01]	[-0.07,-0.04]
Trend change post	-0.018***	-0.021***	-0.021***	-0.022***	-0.007***	0.005***	0.006***	0.020***
	[-0.02,-0.01]	[-0.02,-0.02]	[-0.03,-0.02]	[-0.03,-0.02]	[-0.01,-0.01]	[0.00,0.01]	[0.00,0.01]	[0.02,0.02]
**Estimated change at 6 months after intervention**	**-13.7%**	**-12.5%**	**-9.8%**	**-8.7%**	**-3.3%**	**0.1%**	**0.7%**	**6.6%**
	[-0.154,-0.121]	[-0.147,-0.104]	[-0.117.-0.078]	[-0.111,-0.064]	[-0.048,-0.018]	[-0.011,0.014]	[-0.012,0.026]	[0.047,0.084]
**Continuous Patients**								
Constant	-0.020***	0.008	0.046***	0.059***	0.018***	-0.071***	-0.094***	-0.075***
	[-0.03,0.01]	[-0.00.0.02]	[0.04,0.05]	[0.05,0.07]	[0.01,0.02]	[-0.08,-0.06]	[-0.11,-0.08]	[-0.08,-0.07]
Baseline trend	0	-0.001	-0.003***	-0.004***	-0.002***	0.005***	0.006***	0.003***
	[-0.00,0.00]	[-0.00,0.00]	[-0.00,0.00]	[-0.01,0.00]	[-0.00,0.00]	[-0.00,0.01]	[0.00,0.01]	[0.00,0.00]
Level change post	-0.047***	-0.021*	0.011	0.029**	0.006*	-0.023***	-0.024**	-0.037***
	[-0.06,-0.03]	[-0.04,-0.00]	[-0.00,0.03]	[0.01,0.05]	[0.00,0.01]	[-0.03,-0.01]	[-0.04,-0.01]	[-0.05,-0.02]
Trend change post	-0.014***	-0.018***	-0.018***	-0.020***	-0.004***	0.005***	0.006***	0.018***
	[-0.02,-0.01]	[-0.02,-0.01]	[-0.02,-0.01]	[-0.02,-0.01]	[-0.01,-0.00]	[0.00,0.01]	[0.00,0.01]	[0.01,0.02]
**Estimated change at 6 months after intervention**	**-13.3%**	**-12.8%**	**-10.0%**	**-9.1%**	**-1.7%**	**0.9%**	**1.4%**	**7.3%**
	[-0.149,-0.116]	[-0.157,-0.098]	[-0.120,-0.079]	[-0.115,-0.066]	[-0.026,-0.008]	[-0.005,0.023]	[-0.010,0.039]	[0.050,0.095]

Note:

^**&**^ Denominators decline due to dropout during the follow-up period. A total of 1432 patients in the intervention group and 969 patients in the control group had one or more clinic visits in the last three months of follow-up. 95% confidence intervals in brackets.

* p < 0.05,

** p < 0.01,

*** p < 0.001, PDC = Proportion of Days Covered.

Secondary analyses restricted to the subgroup of women who remained in treatment until at least the final three months of follow-up were similar to the overall cohort (Table 2). In addition, there was no evidence of discontinuities in measured population characteristics at the time of the intervention in either the intervention or control group, and more importantly, no evidence of any change in the differences in these characteristics between groups (S1 Fig).

## Discussion

The intervention, which consisted of implementing a paper-based system of appointment-tracking, community outreach to patients who miss appointments, two days of training, and four subsequent supervisory visits, significantly improved appointment-keeping and consistent availability of antiretroviral medicines for patients on long-term ART. The percentage of women with 95% or more coverage of dispensed medicines increased significantly, a critical outcome for reducing the likelihood of drug resistance. Although we were unable to determine if women took their medicine correctly, more patients had medicines in their possession.

At baseline, the percentage of appointments missed ranged from 21% to 48% across clinics, indicating considerable room for improvement. Averaged across all clinics, the rates of missed visits of various durations for women in the intervention group declined following the intervention, while the rate of missed visits in control clinics increased considerably. The increase in the control group was not unexpected. One inclusion criterion for the study for established patients was that they had recently attended the clinic and were thus in active treatment. Selection based on clinic attendance typically results in artifactually higher rates of attendance for a short period with regression to the underlying population mean over subsequent periods [22]. The fact that the attendance rates among members of the intervention group continued to increase over time is evidence for the effectiveness of the intervention.

We obtained similar results in a secondary analysis of attendance rates in the subgroup of patients who continued to attend the clinic after January 2016, which eliminates bias due to differential dropout. The evidence for the internal validity of findings is strong and includes randomized design, visible separation of post-intervention trends in the two study groups, statistically significant findings from the ITS segmented regression models, and no indication of potential confounding in secondary analyses.

The intervention required clinic staff to modify their systems of care in the clinic and then to communicate these changes to patients, who typically attend clinic once a month to receive a month’s supply of medicines. The time series graphs suggest that the effects of the intervention increased over time. Continuing attention to sustaining the new practices is likely to increase intervention effects even further from what we observed in this trial.

Success in avoiding missed visits and maintaining women on antiretroviral medications varied widely across clinics at baseline, and likewise, responses to the intervention also varied. Reports from supervision visits suggested that clinics’ fidelity to the intervention differed, and that the initial training on the appointment system was insufficient to change behaviour. Many clinics received several supervision visits before they successfully implemented the recommended changes, and supportive supervision is likely needed to solidify similar changes in clinic practice. However, even with supervision, not all clinics were able to reduce rates of missed appointments. Issues such as staff turnover, high patient volume, and availability of community resources for tracking patients must be addressed to ensure success.

A key concern in Option B+ clinics is retention of women in antiretroviral treatment after they deliver their babies. Subgroup analyses in this study (unreported) did not indicate a differential effect of the intervention among women who had and had not yet delivered. However, the study was not designed specifically to examine that issue. Future studies will need to determine whether similar interventions can be helpful in retaining post-partum women in care.

Our results have implications for a national scale-up of Option B+ in Tanzania. All Option B+ clinics had previously been given the appointment books and patient-tracking registers by the national HIV/AIDS control programme. However, clinics were not observed to be using these materials prior to this trial. Dissemination of materials requiring a change in clinic processes is unlikely to ensure adoption.

In addition to the current study, there is evidence from HIV clinics in both Tanzania and Kenya demonstrating the effectiveness of paper-based appointment systems and community outreach as a system-level strategy to improve continuity of ART over a 6- to 12-month follow-up period [17,23]. It remains to be seen if effects persist over a longer period. Further research might examine whether annual supportive supervision visits might be sufficient to maintain the improvements in attendance.

Discussing the date and time of appointments is a more respectful way of engaging patients in long-term treatment than simply instructing them to come back on a certain day whether it is convenient or not. Furthermore, the intervention is inexpensive to implement. National implementation of this approach by the MOHSW, in collaboration with regional health offices and implementation partners, would be considerably less costly than it was in this trial, especially if incorporated into routine supervisory activities. Policy makers in all countries should consider adopting similar systems in ART clinics.

This study suffered from some of the challenges of implementation research in a real-world setting. The data collected came from routine clinic records, and the quality of the information recorded in medical and pharmacy records varied. Errors in recorded dates occurred frequently, including incorrect years (especially at the beginning of a new year), transposed dates or the same dates used for the current and next scheduled visit, and reversal of numbers for day and month. However, these errors were not differential by study group and therefore did not bias our results.

In the early implementation of Option B+ in RCH clinics, insufficient attention was paid to the consistency and quality of record keeping. Staff in these clinics were familiar with records for maternal health care, but the information requirements of an ART programme were new and different for them. We also hoped to collect additional covariates such as CD4 counts, occurrence of medication-related side effects, and reported reasons for poor adherence, but they were inconsistently recorded in the CTC-2 cards. Programmes must pay greater attention to the reliable collection and recording of data that are essential for HIV treatment and long-term patient management. In addition, the English-language appointment and patient-tracking registers designed for ART clinics were sent out to Option B+ RCH clinics without adaptation; the materials would benefit from translation of the forms into Kiswahili.

## Conclusion

We have shown that consistent clinic attendance by HIV-positive patients can be improved by orienting and supporting clinic staff in Option B+ RCH clinics to use a manual appointment and patient-tracking system. The new system empowered clinic staff to plan clinic workloads better, monitor attendance, and follow-up on patients who missed their appointments. Using easy-to-produce summaries, staff could monitor progress and engage in continuous performance quality improvement. To our knowledge this is the first time such improvement has been shown in a nonspecialized HIV/AIDS clinic.

With chronic diseases ever more prevalent and African health care systems having to develop capacity to manage them more effectively, finding ways to improve adherence to appointments and maintain continuity of therapy are vital. Lessons learned from introducing a low-cost appointment and patient-tracking system in Option B+ clinics are valuable in moving toward a broader chronic illness care model. Evidence now exists to justify scaling up this approach to all ART and Option B+ RCH clinics in Tanzania, as well as to test such an intervention in general medical clinics treating other chronic health conditions.

## Supporting information

S1 Table Summary of patients, visits, and study outcomes before and after the intervention among all established patients. (DOCX)

S1 Fig Comparison of the prevalence of key population characteristics in intervention and control patients during the study period. The figures present the monthly values of four key population characteristics from August 2014 until March 2016 averaged across all clinics in the intervention (solid lines) and control (long dashed lines) groups. The intervention began in July 2015 (represented by the solid vertical lines) and continued with monthly supervisory visits in the following four months.
(TIF)

S1 Appendix CONSORT 2010 checklist. (DOCX)

S2 Appendix Ethical review board approved protocol. (DOCX)
