# Supplementary material for: A group randomized trial using an appointment system to improve adherence to ART at reproductive and child health clinics implementing Option B+ in Tanzania
Source: PLoS One. 2017 Sep 28;12(9):e0184591. doi: 10.1371/journal.pone.0184591 (PMC5619716; doi:10.1371/journal.pone.0184591)
Supplement: S1 Table — (DOCX) [file pone.0184591.s001.docx]

**S1 Table: Summary of patients, visits, and study outcomes before and after the intervention among all established patients**
