# Supplementary figures and images for: A group randomized trial using an appointment system to improve adherence to ART at reproductive and child health clinics implementing Option B+ in Tanzania
Source: PLoS One. 2017 Sep 28;12(9):e0184591. doi: 10.1371/journal.pone.0184591 (PMC5619716; doi:10.1371/journal.pone.0184591)

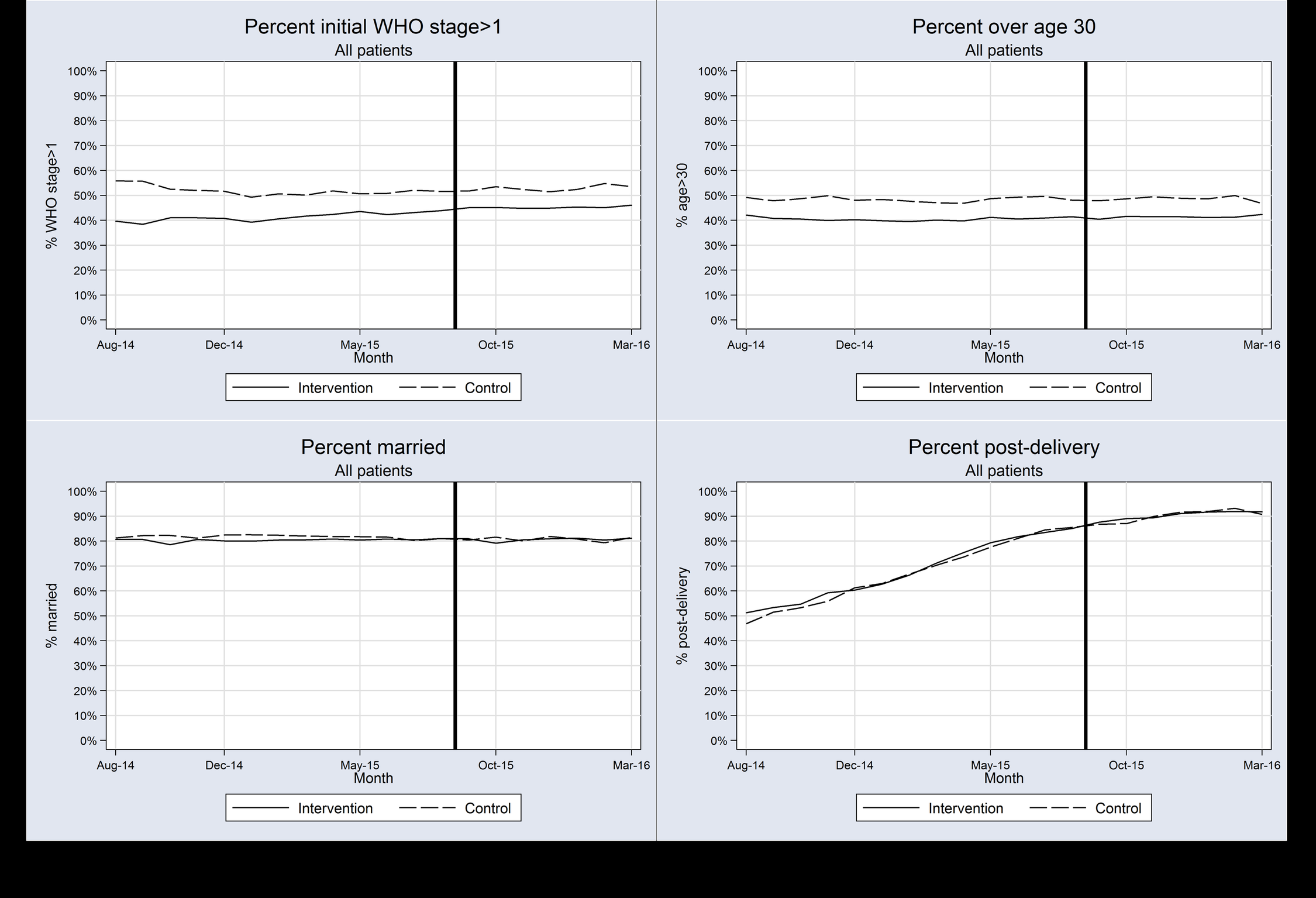

Supplement: S1 Fig — The figures present the monthly values of four key population characteristics from August 2014 until March 2016 averaged across all clinics in the intervention (solid lines) and control (long dashed lines) groups. The intervention began in July 2015 (represented by the solid vertical lines) and continued with monthly supervisory visits in the following four months. (TIF) [file pone.0184591.s002.tif]
