# Supplementary material for: A group randomized trial using an appointment system to improve adherence to ART at reproductive and child health clinics implementing Option B+ in Tanzania
Source: PLoS One. 2017 Sep 28;12(9):e0184591. doi: 10.1371/journal.pone.0184591 (PMC5619716; doi:10.1371/journal.pone.0184591)
Supplement: S2 Appendix — (DOCX) [file pone.0184591.s004.docx]

Improving Adherence to ART at Reproductive and Child Health Clinics Integrating Option B+ in Tanzania

Principal Investigators:

Dr. Suleiman Kimatta (MSH)

Dr. Mwikemo Deborah Kajoka (PMTCT MOHSW)

Ms. Salama Mwatawala, (MSH)

Project Coordinators: Mr. Jafary Liana (MSH)

Ms. Levina Lema (PMTCT MOHSW)

Qualitative Research Coordinator

Dr. Angel Dillip (Ifakara Health Institute)

Research advisors:

Dr. John Chalker (Center for Pharmaceutical Management, Management Sciences for Health, Arlington, Virginia, USA)

Prof. Dennis Ross-Degnan, (Harvard Pilgrim Health Care Institute, Drug Policy Research Group, Dept. of Population Medicine)

Dr. Catherine Vialle-Valentin (Harvard Pilgrim Health Care Institute, Drug Policy Research Group, Dept. of Population Medicine)

# ACRONYMS

| ADDO | accredited drug dispensing outlet |
| --- | --- |
| AIDS | acquired immunodeficiency syndrome |
| ART | antiretroviral therapy |
| CTC | care and treatment clinics |
| DPRG | Drug Policy Research Group (Harvard) |
| HBC | home based care |
| ITS | interrupted time series |
| INRUD | International Network for Rational Use of Drugs |
| INRUD-IAA | INRUD Initiative on Antiretroviral Adherence |
| IMCI | integrated management of childhood illness |
| MOHSW | Ministry of Health and Social Welfare |
| MSH | Management Sciences for Health |
| NACP | National AIDS Control Programme |
| PMTCT | prevention of mother-to-child transmission |
| RCH | reproductive and child health |
| SDSI | Sustainable Drug Seller Initiatives |
| WRP | Walter Reed Program |

# TABLE OF CONTENTS

Contents

[ACRONYMS 2](#_Toc411863309)

[TABLE OF CONTENTS 3](#_Toc411863310)

[SUMMARY 5](#_Toc411863311)

[1.0. INTRODUCTION 7](#_Toc411863312)

[1.1 Background 7](#_Toc411863313)

[1.2 Statement of the Problem 7](#_Toc411863314)

[1.3 Rationale of the study 8](#_Toc411863315)

[1.4 Theory of Change 8](#_Toc411863316)

[1.5 Objectives 11](#_Toc411863317)

[1.5.1 Broad Objective 11](#_Toc411863318)

[1.5.2 Specific Objectives 11](#_Toc411863319)

[1.6 Description of Proposed Interventions 12](#_Toc411863320)

[2.0 METHODOLOGY FOR IMPACT EVALUATION 13](#_Toc411863321)

[2.1 Overview of Impact Evaluation 13](#_Toc411863322)

[2.2 Identification strategy 14](#_Toc411863323)

[2.3 Analytic Methods 14](#_Toc411863324)

[2.4 Qualitative Research 16](#_Toc411863325)

[2.5 Minimizing Bias 17](#_Toc411863326)

[2.6 Data collection, sampling, statistical approach, and power calculations 17](#_Toc411863327)

[2.7 Electronic Data Collection 18](#_Toc411863328)

[2.8 Qualitative Research 18](#_Toc411863329)

[2.9 Cost effectiveness 18](#_Toc411863330)

[2.10 Analytical Methods 18](#_Toc411863331)

[2.11 Policy impact 19](#_Toc411863332)

[3.0. ETHICAL ISSUES 20](#_Toc411863333)

[4.0 PROGRAM IMPLEMETATION AND WORKPLAN 20](#_Toc411863334)

[5.0 STAFFING PLAN 24](#_Toc411863335)

[6.0 ORGANIZATIONAL QUALIFICATIONS 26](#_Toc411863336)

[6.1 Management Sciences for Health 26](#_Toc411863337)

[6.2 Harvard Pilgrim Health Care Institute 29](#_Toc411863338)

[7.0 BUDGET AND BUDGET JUSTIFICATION 30](#_Toc411863339)

[8.0 DATA COLLECTION FORMS 32](#_Toc411863340)

[8.1 Quantitative Forms in English 32](#_Toc411863341)

[8.1.1 Facility Form (1 per facility) 32](#_Toc411863342)

[8.1.2 Patient Form (1 per patient) 33](#_Toc411863343)

[8.1.3 Visit Form (1 per visit) 34](#_Toc411863344)

[8.2 Interview guidelines in English 35](#_Toc411863345)

[8.2.1 District Health Officials interview guidelines 35](#_Toc411863346)

[8.2.2 RCH Clinic Staff interview guidelines 40](#_Toc411863347)

[8.2.3 Patient exit interview guidelines 45](#_Toc411863348)

[8.3 Quantitative Forms in Kiswahili 48](#_Toc411863349)

[8.3.1 Facility Form (1 per facility) 48](#_Toc411863350)

[8.3.2 Patient Form (1 per patient) 49](#_Toc411863351)

[8.3.3 Visit Form (1 per visit) 50](#_Toc411863352)

[8.4 Interview guidelines In Kiswahili 50](#_Toc411863353)

[8.4.1 District Health Officials interview guidelines in Kiswahili 51](#_Toc411863354)

[8.4.2 RCH Clinic Staff interview guidelines in Kiswahili 56](#_Toc411863355)

[8.4.3 Patient exit interview guidelines in Kiswahili 62](#_Toc411863356)

[9.0 INFORMATION AND INFORMED CONSENT FORMS 66](#_Toc411863357)

[9.1 Information in English 66](#_Toc411863358)

[9.2 Informed consent form in English 67](#_Toc411863359)

[9.3 Information in Kiswahili 68](#_Toc411863360)

[9.4 Informed Consent Form in Kiswahili 69](#_Toc411863361)

[10.0 CURRICULUM VITAE OF RESEARCHERS 71](#_Toc411863362)

[10.1 Dr. Suleiman Kimatta 71](#_Toc411863363)

[10.2 Dr. Mwikemo Deborah Kajoka (PMTCT) 74](#_Toc411863364)

[10.3 Mrs. Salama Mwatawala 79](#_Toc411863365)

[10.4 Ms. Levina Lema 83](#_Toc411863366)

[10.5 Mr. Jafary Liana 86](#_Toc411863367)

[10.6 Dr. Angel Dillip 89](#_Toc411863368)

[10.7 Dr. John Chalker 94](#_Toc411863369)

[10.8 Dr. Dennis Ross-Degnan 98](#_Toc411863370)

[10.9 Dr. Catherine Vialle-Valentin 102](#_Toc411863371)

# SUMMARY

Adherence to antiretroviral therapy (ART) is the most powerful predictor of treatment success, survival, and drug resistance. A facility’s rate of patients attending appointments on time correlates with medication adherence and clinical outcome. ART clinics in three East African countries, including Tanzania, found that implementing a minimally invasive, low-cost patient appointment and tracking system allowed them to promptly identify missing patients and promote clinic attendance. Tanzania has since adopted Option B-Plus for HIV-positive pregnant women; they will receive ART at reproductive and child health (RCH) clinics rather than at specialized ART clinics.

Until recently, because ART clinics in Tanzania lacked efficient appointment systems, few knew whom they were expecting each day or who had not arrived when expected. By the time patients were identified as lost to follow-up, they may have already missed several months of treatment, which greatly enhanced their risk of resistance and treatment failure. Recent observations by the Ministry of Health and Social Welfare’s (MOHSW) Prevention of Mother-to-Child Transmission (PMTCT) Unit show that RCH clinics are experiencing a similar challenge.

The proposed intervention will comprise two phases focused on 24 RCH facilities in Mbeya region. The intervention team will include representatives from the PMTCT Unit, Management Sciences for Health (MSH), and the district health team. For the first phase, our intervention team will discuss issues of adherence and retention with the staff in each facility using baseline survey findings and including problems the clinic faces and the local resources available. We will then present the appointment system to the facility staff and demonstrate how to use it. We will either introduce or strengthen an existing system to track patients who have missed their appointments and help create a feedback system, so that the facility staff knows the outcome of the contact with the missing patient. In addition, we will teach the staff how to calculate the monthly appointment-keeping indicator from the appointment system as a means of monitoring how well they are doing. The indicator for appointment keeping is the percentage of patients who arrived for their appointment on or before or within three days of the scheduled day. This is an important tool for instituting a culture of continuous quality improvement. For the second phase, the intervention team will make four ongoing supervision visits over the next three months. The first two visits at fortnightly intervals and the next two monthly. The purpose will be to reinforce earlier messages, assist with any technical difficulties, help with developing facility-level appointment-keeping indicators, and promote discussion on how to improve practices.

Our hypothesis is that the introduction of such an appointment system in RCH clinics with community follow-up of non-attendees will improve adherence to appointment keeping and continuity of ART in women who test positive for HIV. To test this, we will implement a group-randomized controlled trial in 24 RCH clinics offering treatment for HIV-positive women in the Mbeya region. Study outcomes will include clinic attendance on the day scheduled (primary outcome); monthly percentage of days covered by dispensed antiretrovirals (ARVs); time until a gap in clinic attendance of 15 or more days; and time until loss to follow-up (defined as no clinic contact for the previous 60 days).

During baseline and endline assessments, our evaluation team will visit each clinic and review clinic and pharmacy records to extract data on visits (visit date, purpose, date of next visit) and medication dispensing (ARV type, amount, days’ supply). At baseline, we will select all women who were established on treatment for 6 or more months at baseline in each facility for our analytic sample (up to a maximum of 200); we will extract data for these women during the follow-up assessment. In addition, to provide information on key study outcomes in the early phase of treatment, at the follow up assessment we will collect data on a newly treated cohort. In each clinic, up to 5 newly started patients per month will be included in the sample each month for 6 months before and 6 months after the intervention, making a total of up to 60 newly treated patients per facility.

We think it would increase the possibility of contamination to have intervention and control facilities within the same districts, so we will pair districts in the region by the numbers of facilities with 75 or more patients on ART. These 24 clinics each average more than 120 patients currently in treatment.

The baseline assessment will include these data retrospectively for 10 months, while the follow-up assessment will include data retrospectively to the date of the baseline assessment. In total, the study period will include 12 months before the beginning of intervention implementation and seven months after implementation.

At baseline we will also interview key intervention district and clinic staff and patients to address questions concerning perceptions about clinic efficiency, problems in adherence and retention, and patient engagement with care. At endline, we will interview both intervention and control district and clinic staff and patients to assess their perceptions of the intervention and whether any other initiatives had taken place.

The data will be collected on handheld tablets. Assimilating the data from the tablets, we will use aggregate and individual-level interrupted time series (ITS) models to compare changes in the rate of missed visits in intervention versus control clinics before and after implementation of the appointment and patient-tracking intervention. We will use Kaplan-Meier and accelerated failure time methods to assess the impact of appointment systems on 15-day gaps in visits and greater than 60-day loss to follow-up. Our statistical comparison will be the times-to-event in the post-intervention period in intervention versus control patients, relative to the times-to-event in the baseline period in intervention versus controls. All models will adjust for possible pre-post changes in outcomes in the control group and all models will control for clustering/correlation within health facilities

If successful, the benefit of the introduction of an appointment and patient-tracking system will the empowerment of clinic staff to plan their work schedules, rapidly identify patients who miss appointments, develop ways to follow-up on patients who have missed appointments, and produce monthly appointment-keeping indicator values for continuous quality improvement. For patients, the benefit will be a more patient-centred approach that gives them a choice for an appointment time. The ultimate benefit for the MOHSW is to have increased patient attendance and appropriate use of HIV services in the clinics that improves the health of the HIV-positive mothers and prevents the birth of HIV-positive babies.

# [1.0. INTRODUCTION](#_Toc202681914)

## 1.1 Background

Until recently, all ART for HIV/AIDS was administered through specialized clinics in Tanzania. In the middle of 2014, Tanzania officially adopted Option B-Plus for pregnant women in all their RCH clinics, whereby, all HIV-positive women receive lifelong ART regardless of their CD4 count. This is the first time that ART is being administered outside of specialized clinics. Under Option B-Plus, the RCH clinics will provide ART to women prenatally and for three postnatal years. This innovation means that the RCH clinics have had to integrate HIV care into their routine. However, the MOHSW reports that adherence and retention to treatment are problems under the program. Good adherence to ART is essential for successful treatment and averted resistance to the medicines.

From our previous work in ART clinics, we have shown that a facility’s rate of patients attending appointments on time correlates with medication adherence and clinical outcome.^[[1]](#footnote-1)^ But until recently, very few ART clinics in Tanzania could identify whom they were expecting that day or who had not arrived when expected because of not having efficient appointment systems.^[[2]](#footnote-2)^ By the time patients were identified as lost to follow-up, they may have already missed several months of treatment with a greatly enhanced risk of developing resistance and failing treatment. However, ART clinics in three East African countries found that implementing a minimally invasive, low-cost patient appointment and tracking system allowed them to identify missing patients promptly, facilitate the management of their workload, and promote sustainable and consistent clinic attendance by HIV-positive patients.^[[3]](#footnote-3),^^[[4]](#footnote-4),^^[[5]](#footnote-5)^

In June 2013, Tanzania adopted Option B-Plus for pregnant women, whereby all HIV-positive women receive lifelong ART, regardless of their CD4 count. Under this plan, the women will receive ART prenatally and for three postnatal years at RCH clinics rather than the specialized ART clinics. This means that the RCH clinics have to integrate HIV care into their routine for the first time. The plan was to implement Option B-Plus in all 4,914 RCH clinics by June 2014. By December 2013, more than 10,000 mothers were on ART, and by June 2014, almost three-quarters of the clinics were implementing Option B-Plus. However, the PMTCT Unit of MOHSW’s Reproductive and Child Health Section has found that ensuring good rates of adherence and retaining patients in care have been problems for them.

The MOHSW’s PMTCT Unit has committed to using the appointment books and systems that were developed in our previous work, but none of the RCH clinic staff understands how to use them.

## 1.2 Statement of the Problem

Patients fail to adhere to their recommended ART for many reasons: not being able to afford the travel to the clinics (economic status); forgetfulness; unpleasant side effects; thinking they are cured (health literacy); education level; social support; and to avoid stigma; amongst many others.^[[6]](#footnote-6)^ Facility-level determinants also contribute to the adherence problem, including poor staff–patient relationships and long waiting times.^[[7]](#footnote-7)^

## 1.3 Rationale of the study

Using routine data from ART clinics, we showed that on-time appointment attendance at clinics dedicated to HIV-positive patients correlated with patients’ medication adherence and clinical outcomes.^[[8]](#footnote-8)^ However, we also showed a wide variation in attendance rates between clinics, ranging from 15% to 100%.^[[9]](#footnote-9)^ This difference implies that system factors can be changed to improve patient adherence. Yet in these settings, appointment scheduling systems are haphazard, if they exist. Staff members record patients’ arrival in a variety of registers or patient diaries, and typically, there is no way to determine either who is expected to show up at the clinic or whether expected patients did come. By the time patients are identified as lost to follow-up, they may have already missed months of treatment.^[[10]](#footnote-10)^

To maximize the chances of treatment success and minimize the development of drug resistance, ART programs need to promptly identify and follow-up with patients who miss appointments, which can be accomplished with an easy-to-use, but effective appointment system. Our research proved that in specialized ART clinics, the introduction of an appointment and patient-tracking system empowered staff to—

- Plan their work schedules
- Control their work load
- Give patients a choice of day and time for an appointment
- Rapidly identify patients who miss appointments
- Develop ways to follow up on patients who have missed appointments
- Produce and discuss monthly appointment-keeping indicator values^[[11]](#footnote-11),^^[[12]](#footnote-12),^^[[13]](#footnote-13)^

We believe that introducing the same appointment and patient-tracking system coupled with training and follow-up monitoring, will have a similar effect in RCH clinics that are integrating Option B-Plus into their routine work. If we can show that orienting the clinics to the appointment system improves patient retention and adherence, then we believe that the MOHSW will support a national orientation program for widespread use and adoption.

## 1.4 Theory of Change

Adherence to ART is the most powerful predictor of treatment success, survival, and drug resistance.^[[14]](#footnote-14),^^[[15]](#footnote-15),^^[[16]](#footnote-16)^ Adherence to antiretrovirals (ARVs) is a complex phenomenon affected by both individual and system-level determinants.^[[17]](#footnote-17)^,^[[18]](#footnote-18)^,^[[19]](#footnote-19)^,^[[20]](#footnote-20)^ Most relevant to the current proposal, being female, under 35 years old, and single have been also found to be significantly associated with nonadherence.^[[21]](#footnote-21)^

In our previous work in the International Network for Rational Use of Drugs Initiative on Antiretroviral Adherence (INRUD-IAA), we used both quantitative and qualitative methods to explore the prevalence and determinants of adherence and appointment keeping in five East African countries. ^[[22]](#footnote-22)^,^[[23]](#footnote-23)^,^[[24]](#footnote-24)^,^[[25]](#footnote-25)^ INRUD-IAA work was informed by the theoretical constructs of Green’s PRECEDE-PROCEED planning model.^[[26]](#footnote-26)^ The goals of the PRECEDE-PROCEED process are to first understand health-related behaviours and environments and then to design and evaluate interventions that can affect the behaviours themselves, the settings that influence them, and their health consequences. A key construct of the model is that health behaviours are influenced by predisposing, enabling, and reinforcing factors that operate at both the individual and environmental level.

We showed that both individual-level and system-level barriers can affect appointment keeping and adherence to medications in East Africa, which ultimately affects optimal clinical outcomes.2 Individual-level factors that we identified included physical discomfort and side effects, forgetting, fear of social rejection, economic resources and ability to pay for ART-related costs, including transportation, and lost labour when attending the clinic. Systems-related factors included clinic congestion and waiting time, insufficient counselling time to ensure that patients understand the drug regimen, inadequate reassurance and supportive feedback, unreliable drug supply causing stock-outs, and inability to identify and reach out to patients who miss visits.

HIV treatment programs are intended to include counselling to address individual barriers to adherence. However, staff often finds it difficult to identify patients who need counselling. For practical reasons, INRUD-IAA focused on developing affordable systems-level interventions that can address both systems-related and individual barriers to adherence (see Figure 1). What emerged was a focus on establishing practical appointment systems for scheduling patients and tracking attendance, community outreach to reconnect with patients who miss appointments, and monitoring overall performance over time. A functional appointment and patient-tracking system helps to enable patients to attend on time by scheduling their visits on an agreed-upon date and time window. Scheduling patients decongests clinics and allows more time for staff to counsel and reinforce adherence messages. Our theory is that by focusing on establishing appointments and identifying and using local resources such as community outreach systems to help patients attend on time, we can reduce both system-level and individual-level barriers. This will be reinforced by establishing an enabling culture of continuous quality improvement through the calculation of an appointment-keeping indicator and monthly staff discussion of facility performance as measured by this indicator. These interventions should reinforce clinic staff commitment to focusing on attendance and encouraging the development of creative solutions to enable patients to be sustained on therapy. Results should be improvements in: (1) patient engagement; (2) patient-staff communication (3) linkage to community support systems; and (4) a facility focus on improving quality of care.
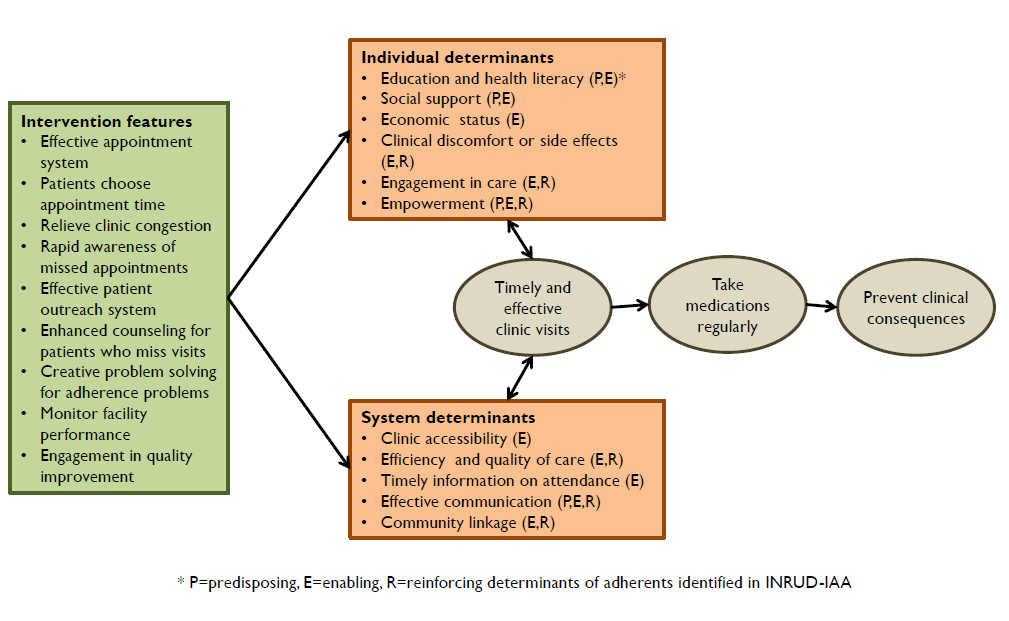


Figure 1: Intervention features addressing individual- and system-level determinants of adherence

We expect that INRUD-IAA’s intervention approach in ART clinics will be even more effective in the new context of RCH clinics where women are treated for HIV infection after being identified through prenatal screening. First, we believe that pregnant women will be motivated to achieve the best outcomes for their children, which requires their own good health. Second, women are initiated on ART upon evidence of HIV infection, rather than after the progression has made them very ill. They have also been counselled on the need for keeping up ART for life; therefore, they will be motivated to remain on treatment. Similarly, RCH staff wish to contribute to the best outcomes for mothers and children and already have a culture of monitoring pregnancy-related indicators. However, the systems in RCH clinics were not developed to monitor adherence of long-term therapy or track clinic attendance or reach out to women who fail to appear for visits, which is the focus of this intervention.

Priority Geographic Areas

The PMTCT Unit has compiled numbers of women by region and district who are HIV-positive and on ART. The Unit sees Mbeya region as a priority. We got information on the number of women on ART per facility from the PMTCT Unit and the Walter Reed Program in Mbeya (Table1).

Table 1: Illustrative numbers on ART by facility and district in Mbeya (for facilities with 75 or more women on ART) as of August 2014

| **District** | **Health facility** | **Total on ART** |
| --- | --- | --- |
| **Busokelo DC** | Mwakaleli Health Centre | 86 |
| **Chunya DC** | Chunya Hospital | 175 |
|  | Makongolosi Dispensary | 119 |
|  | Mwambani Designated District Hospital (DDH) | 226 |
| **Kyela DC** | Ipinda Health Centre | 121 |
|  | Kyela District Hospital | 205 |
| **Mbarali DC** | Chimala Mission Hospital | 191 |
|  | Mbarali District Hospital | 382 |
|  | Small Holders Dispensary | 82 |
|  | St Bhakita Health Centre | 109 |
|  | Ujewa Mission Dispensary | 86 |
| **Mbeya City Council** | Igawilo Health Centre | 306 |
|  | Kiwanjampaka Health Centre | 267 |
|  | Mbeya Regional Hospital | 108 |
|  | Meta Hospital | 265 |
|  | Ruanda Health Centre | 395 |
| **Mbeya DC** | Ifisi Hospital | 196 |
|  | Mbalizi JWTZ Hospital | 101 |
|  | Mbalizi Mission Hospital | 103 |
| **Mbozi DC** | Itaka Dispensary | 102 |
|  | Mbozi Hospital | 82 |
|  | Mlowo Dispensary | 93 |
|  | Vwawa Hospital | 164 |
| **Momba DC** | Kamsamba Health Centre | 86 |
|  | Tunduma Health Centre | 256 |
| **Rungwe DC** | Igogwe Mission Hospital | 122 |
|  | Tukuyu Hospital | 98 |
| **Grand Total** |  | **4526** |

## 1.5 Objectives

### 1.5.1 Broad Objective

Develop an intervention strategy and measure its effects in improving adherence to ART in RCH clinics operating Option B-Plus in Tanzania.

### 1.5.2 Specific Objectives

1. To establish baseline measures for rates of adherence measured through indicators of—

- Clinic attendance on the day scheduled (primary outcome)
- Clinic attendance within three days of scheduled date
- Clinic attendance within seven days of scheduled date
- Percentage of days covered by dispensed ARVs
- Time until a gap in clinic attendance of 15 or more days
- Time until loss to follow-up (defined as no clinic contact for previous 60 days)
- Time until loss to follow-up (defined as no clinic contact for 60 days from missed appointment)

1. To evaluate the effectiveness of restructuring the appointment system to make the date negotiable and the appointment for a specific period of the day in increasing attendance on the day of the appointment and in reducing patient waiting time
2. To evaluate the effectiveness of strengthening the patient-tracking system in improving the identification of patients who have not attended and speeding up their return to the clinic

## 1.6 Description of Proposed Interventions

The intervention will be comprised of two phases, focused on 24 randomly selected RCH facilities in Mbeya region. Activities during the first phase include four stages which will be accomplished during the first two-day visit by the intervention team to each facility. The facility selection process is covered in the evaluation section. The intervention team will comprise representatives from MSH and the PMTCT Unit of the MOHSW.

*First Phase*

**Stage 1**: Our intervention team will discuss issues of adherence and retention with the staff in each facility based on the findings of the baseline survey (described in the evaluation section). By introducing the appointment-keeping indicator and comparing the baseline results of each clinic visited to the baseline results in other clinics, problems the clinic faces and the local resources available will be discussed.

**Stage 2**: We will present the appointment system to the facility staff and demonstrate how to use it. An important and popular feature of the system is the introduction of negotiated appointments based on patients’ preferences for appointment day and time. Currently, all patients arrive early in the morning and then wait for up to five hours to be seen. Time lost waiting is a great disincentive to attend monthly, which a chronic condition such as HIV/AIDS requires. In the new system, each day will be divided into three blocks: morning, mid-morning, and afternoon.

Where the clinics are using paper-based systems, we will introduce a standardized paper-based appointment register that will enable staff to effectively monitor appointment keeping. They will use it to record—

- Which patients are expected that day and in which block of time
- Whether they come or not on that day
- If they do not come that day, whether they attend within three days after the appointment and the reasons for missing scheduled appointments
- How many come on each day without an appointment

The reason for noting whether patients attended within three days of their appointment is because they have an appointment every four weeks, but are given packets containing 30 days of medication. Therefore, if the patient is three days late, they may not have missed any doses of medication. Our previous research validated this three-day indicator.^[[27]](#footnote-27)^

**Stage 3**: We will either introduce or strengthen a system to track patients who have missed their appointments. The first step will be to ensure that the facility has adequate contact information for each patient. If not, staff will need to verify contact information on the patient’s next visit. The new appointment register will be used to rapidly identify patients who miss appointments and then their information will be transferred to the new tracking register.

A patient who has missed an appointment needs to be contacted in some way, which will depend on local resources. In many districts, community organizations could be recruited to follow-up on missing patients, if the clinic knew who was missing and had a relationship with the organization. Other patients who live nearby could be recruited to visit the missing patient, or someone could contact them through mobile phone. The staff members at each facility will determine which option is the most feasible for their particular clinic. The final step of Stage 3 will be the creation of a feedback system, so that the facility staff knows the outcome of the contact with the missing patient.

**Stage 4**: We will teach the staff how to calculate the monthly appointment-keeping indicator from the appointment system as a means of monitoring how well they are doing. The indicator for appointment keeping is the percentage of patients who arrived for their appointment on or before or within three days of the scheduled day. By assessing this each month, the clinic staff can monitor their progress and discuss the results and possible solutions at monthly staff meetings. This is an important tool for instituting a culture of continuous quality improvement.

*Second Phase*

The intervention team will make ongoing supervision visits over the next three months, the first two visits at fortnightly intervals and the next two monthly. The purpose will be to reinforce earlier messages, assist with any technical difficulties, help with developing facility-level appointment-keeping indicators, and promote discussion on how to improve them.

# 2.0 METHODOLOGY FOR IMPACT EVALUATION

## 2.1 Overview of Impact Evaluation

The primary question we wish to answer in this impact evaluation is whether the introduction of an appointment system in RCH clinics in Tanzania with community follow-up of non-attendees improves adherence to appointment keeping and continuity of ART for HIV-positive women. Building on an intervention model developed for HIV/AIDS clinics in INRUD-IAA,^^[[28]](#footnote-28)^,^[[29]](#footnote-29)^,^[[30]](#footnote-30)^,^[[31]](#footnote-31)^^ as described above, intervention components will include: (1) introduction of an appointment and patient-tracking system, (2) training and supervising RCH clinic staff on how to use a paper-based appointment system, (3) daily tracking of attendance and missed appointments for women on HIV treatment, (4) development of localized systems for community outreach to women who fail to attend scheduled appointments within three days, (5) monthly tracking of key facility performance measures by clinic staff; and (6) supervisory visits for problem solving during implementation.

Study outcomes will include: clinic attendance on the day scheduled (the primary outcome); clinic attendance within three days and seven days of scheduled date; monthly percentage of days covered by dispensed ARVs; time until occurrence of a gap in clinic attendance of 15 or more days; and time until loss to follow-up (defined a) as no clinic contact for the previous 60 days); and retention rates defined as no clinic visit within 60 days of a missed appointment. As mentioned, we have shown that most of these measures can be reliably collected in health facilities in East Africa using routine data, and that they predict clinical outcomes.^^[[32]](#footnote-32)^,^[[33]](#footnote-33)^^

In addition to assessing quantitative changes in these measures of clinic attendance, medication adherence, and dropout, we will also use baseline and post-intervention semi-structured interviews with key district health officials and clinic staff to address secondary questions concerning perceptions about clinic efficiency, problems in adherence and retention, and patient engagement with care. In intervention clinics we will interview staff about whether the intervention was acceptable and implemented efficaciously. We will also conduct brief surveys with women attending RCH clinics for ART to collect information about their perceptions of clinic processes as well as perceived barriers to clinic attendance and adherence. The baseline qualitative interviews will only be conducted in intervention clinics to avoid contamination.

## 2.2 Identification strategy

To answer our study questions, we have purposively selected Mbeya, which the MOHSW has identified as a priority region because of high prevalence of HIV-positive pregnant women (13%). We think it would increase the possibility of contamination to have intervention and control facilities within the same districts, so we will purposively select 24 clinics with the most women on ART, and then pair districts in the region by the numbers of these selected facilities and randomly select one district of each pair as intervention and one as control, to end with 12 intervention and 12 control facilities. These 24 clinics each average more than 120 patients currently in treatment. Illustratively from table 1 there are 27 clinics with 75 or more women on ART so would pair districts in the region by the numbers of facilities with 75 or more patients on ART. For this exercise we would eliminate Busokelo district since it only had one facility with 75 or more patients. Then we would pair the other eight districts based on number of facilities and number of women on ART (Table 2). We would then randomly select one of each pair for intervention.

**Table 2: Illustrative pairing of districts based on number of facilities and number of women on ART**

| **Random** | **District** |  | **If ≥75 women on ART** |  |
| --- | --- | --- | --- | --- |
| **Group** | **Pair** | **District** | **# facilities** | **# women** |
| 1 | 1 | Mbarali DC | 5 | 850 |
| 2 | 1 | Mbeya City Council | 5 | 1341 |
| 1 | 2 | Chunya DC | 3 | 520 |
| 2 | 2 | Momba DC | 2 | 342 |
| 1 | 3 | Kyela DC | 2 | 326 |
| 2 | 3 | Rungwe DC | 2 | 220 |
| 1 | 4 | Mbozi DC | 4 | 441 |
| 2 | 4 | Mbeya DC | 3 | 400 |
|  |  | Busokelo DC | 1 | 86 |
|  |  | **Total** | **9052** | **4526** |

## 2.3 Analytic Methods

All quantitative data will be collected by tablet and downloaded onto a local server run by Invention and Technological Ideas Development Organization (ITIDO) which is a local Tanzanian organization. No data will be sent out of the country. All analysis will be performed on the local server. International collaborators will only be providing technical assistance. Our main analytic method will be interrupted time series (ITS) with comparison series analysis,^^[[34]](#footnote-34)^,^[[35]](#footnote-35)^^ comparing changes in clinic attendance and medication adherence in the intervention and control groups from 10 months before to 7 months after initial intervention implementation. We will use both aggregate and individual level ITS models.^7,^[[36]](#footnote-36)^^ To evaluate changes in time until gaps in clinic attendance and time until loss to follow-up, we will use Kaplan-Meier survival curves with accelerated failure time models,^^[[37]](#footnote-37)^,^[[38]](#footnote-38)^^ to compare pre- and post-intervention adjusted survival curves in the intervention and control groups.

Random allocation of health facilities to receive the intervention is our primary strategy to increase internal validity and strengthen causal inference about intervention effects; pre-post changes in randomly assigned control facilities will represent the counterfactual, while pre-post changes in intervention facilities minus pre-post changes in control facilities will represent intervention effects. Random allocation minimizes the likelihood of selection bias as well as most other threats to internal validity.^^[[39]](#footnote-39)^,^[[40]](#footnote-40)^^ By assigning facilities to district pairs, we maximize geographic separation between facilities to minimize contamination. In addition, while ITS with comparison series analyses of group-randomized clinical trials has been shown to produce results that are generally equivalent to more typical difference-in-difference analyses of randomized clinical trial designs,^^[[41]](#footnote-41)^,^[[42]](#footnote-42)^^ this type of longitudinal analysis has the additional advantage of being able to also adjust for differences in pre-intervention trends between groups, which are commonly observed in group-randomized trials with relatively small numbers of groups.^^[[43]](#footnote-43)^ ,^[[44]](#footnote-44)^^ Furthermore, ITS analysis can detect dynamic effects that increase or decrease over the course of the post-intervention period, which may occur during and after the period of post-intervention supervisory visits. Similarly, segmented survival models of time until gaps in clinical attendance and dropout can establish the equivalence of the baseline hazard functions in the intervention and control groups prior to the intervention and compare changes in hazard functions between groups post intervention (see Figures 2 and 3).

All women on ART in the RCH facilities in the intervention districts will receive the intervention. Our analytic sample will be taken from 12 months before through 7 months after the intervention and will include: a) all women (up to of 200 per facility) who were established on treatment for 6 or more months at baseline: and b) up to 5 newly treated patients per facility per month before and after the intervention.

We will capture data on all visits by these women to the sampled health facilities during the entire study observation period. Our individual-level ITS and survival models will use data on patient demographics and clinical characteristics to adjust for differences between intervention and control groups and to accommodate changes in the study population over time.

**Figure 2. Hypothetical effect of appointment and patient tracking system on rate of missed clinic visits**


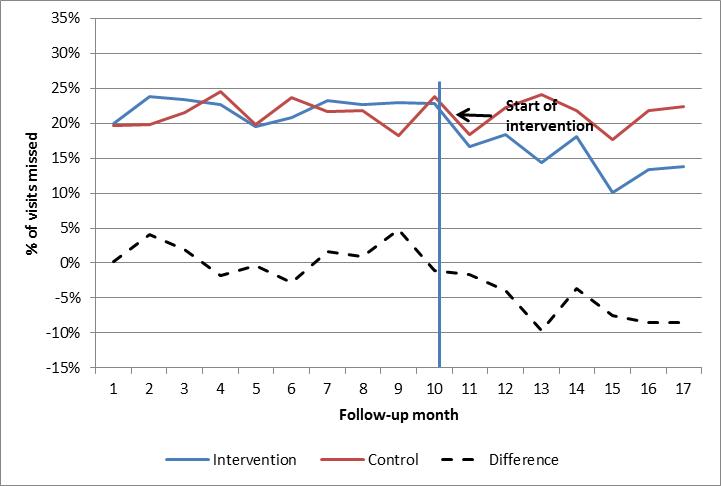


**Figure 3. Hypothetical effect of appointment and patient tracking system on time until 15-day gap in care**


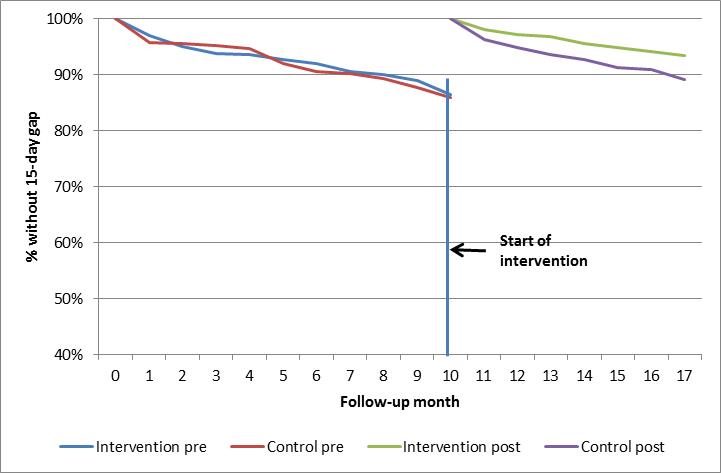


## 2.4 Qualitative Research

The qualitative data that we collect during baseline and follow-up assessments will allow us to learn about notable baseline differences among clinics, for example, how clinics manage attendance and their linkages to community programs, as well learn about and potentially adjust for any program or policy changes that have occurred in these facilities during the follow-up period. Post-intervention qualitative data will also provide context to understand observed intervention effects, for example, by suggesting reasons why the intervention is more successful in some facilities or to detect contamination.

## 2.5 Minimizing Bias

Real-world trials are subject to several threats to internal and external validity. A group-randomized trial of districts located within the same administrative area (i.e., region) is subject to contamination of control districts because staff may hear about the intervention through regular communication with peers or at regional meetings. We will work with regional and district MOHSW officials to engage them in the design of the intervention and encourage them to limit communication about it during the follow-up period. Using qualitative interviews in control facilities at the end of the study, we will explore the degree of knowledge among clinical staff about the intervention and assess whether these facilities adopted similar approaches to improve attendance, medication adherence, or community outreach during the follow-up period. The baseline assessment process also introduces the possibility of observation bias; behaviours related to appointment keeping or adherence can be influenced by the measurement process. However, the randomized design and our longitudinal analytic methods can adjust for changes in both groups that might occur following baseline measurement.

## 2.6 Data collection, sampling, statistical approach, and power calculations

In each district we will conduct a baseline assessment two months prior to the intervention and a follow-up assessment seven months after implementation. During each assessment, our evaluation team will visit each clinic and review clinic and pharmacy records to extract data on visits (visit date, purpose, date of next visit) and medication dispensing (ARV type, amount, days’ supply). At baseline, we will select all women (up to 200 per facility) who were established on treatment for 6 or more months at baseline: we will extract data for these women during the follow-up assessment. At follow up we will add a cohort of newly treated patients, this will provide information on key study outcomes in the early phase of treatment. In each clinic, up to 5 new patients per month will be included in the sample each month for 6 months before and 6 months after the intervention, making a total of up to 60 newly treated patients.

. In addition to data on women’s demographics (age, location of residence, marital status, employment) and clinical details (date of initial HIV test, CD4 at ART initiation, date of ART initiation, initial ART regimen, current ART regimen), the team will record data on the dates of monthly appointment attendance and details about medication regimen and dispensing at each visit. The baseline assessment will include these data retrospectively for 10 months, while the follow-up assessment will include data retrospectively to the date of the baseline assessment. In total, the study period will include 12 months before the beginning of intervention implementation and seven months after implementation.

Our primary outcome in this project is missed clinic visits on the scheduled day, as measured from data in clinical records. We will use aggregate and individual-level ITS models to compare pre-post changes in the rate of missed visits in intervention versus control clinics following implementation of the appointment and patient tracking intervention.

Five of our outcomes (missed visits, visits missed by more than 3 days and 7 days, time until occurrence of a gap in clinic attendance of 15 or more days, and time until loss to follow-up) are derived from dates recorded in medical or pharmacy records. At each completed visit, most clinics record the date of the next expected visit in the patient’s record. If dates are missing, we will base the expected visit date on the number of days of medication dispensed; alternatively, some clinics employ a standard visit interval based on the amount of medication dispensed (e.g., 28 days). The first two outcomes are specified as rates, while the second two are time intervals from the beginning of follow-up (in the baseline period) or the start of the appointment system (in the follow-up period) until the occurrence of the outcome.

We used PASS software, with both 0.001 and 0.01 as estimated values of the intra class correlation coefficient to calculate power, with the higher value resulting in lower power. Typical values for estimating the intra class correlation coefficient range from 0.002 to 0.05 (a wide range). Since missed visits are due to individual as well as system-level issues, we expect relatively low correlations between individuals within each cluster; thus, we used the smaller range of 0.001 to 0.01. The overall rate of missing clinic appointments typical in HIV/AIDS clinics in the baseline period of our previous study was approximately 30%. In this context, we would have 80% power to detect change in rate of missed visits of 4.6% versus 6.0%, assuming 0.001 and 0.01, respectively. The formula is from Donner and Klar.^^[[45]](#footnote-45)^^

## 2.7 Electronic Data Collection

A unique feature of our data collection will be the use of handheld tablets that allow electronic capture of data. In addition to protecting against missing or wild data values, electronic capture will allow us to get preliminary data on baseline rates of missed appointments quickly enough to allow post-collection blocking of facilities prior to randomization. Electronic capture of follow-up assessment data will allow us to maximize the time that the intervention will run at steady state in order to detect dynamic post-intervention trends. We have recently used similar tablet technology to collect data in both health facilities and households during a multicomponent community assessment in Tanzania. Data on tablets will be downloaded onto a local server run by a local Tanzanian organization: Invention and Technological Ideas Development Organization (ITIDO). All analysis will be performed on the local server.

## 2.8 Qualitative Research

During baseline data collection, the teams will interview clinic staff in intervention clinics about whether and how patients who miss appointments are recognized, what is done in response, and what potential local resources may exist (e.g., community HIV support programs, community health workers) to follow up. During both the baseline and follow-up assessments, the team will also conduct semi-structured interviews with district health officials and clinic staff in both intervention and control groups about their perceptions regarding clinic efficiency, problems in adherence and retention, engagement with care. Finally, at follow-up, the teams will question staff in the intervention clinics about their perceptions of the intervention and its effects, and in control clinics on whether there have been any changes in the previous seven months and whether there was any awareness of the interventions implemented in the other districts.

During both assessments, data collectors will conduct semi-structured exit interviews with five women per facility on ART to explore perceived barriers to attendance and medication adherence and their perceptions about clinic efficiency and communication with the care team. This sample size will not be sufficient to allow quantitative comparisons between intervention and control groups; however, we can explore possible changes in patient perceptions following an intervention intended to make clinic care more patient-centred.

## 2.9 Cost effectiveness

We will add a cost-effectiveness analysis. To help with this, we will give diaries to those doing the intervention and supervision to monitor their activities and calculate person days of effort. We will also monitor other intervention costs, such as printing, training, or refreshments at implementation meetings.

## 2.10 Analytical Methods

We will use two rigorous statistical approaches to evaluate program effects: ITS with comparison series and Kaplan-Meier survival curves with accelerated failure time models. All models will adjust for possible pre-post changes in outcomes in the control group and all models will control for clustering/correlation within health facilities.

We will use ITS with comparison series to assess baseline to endline changes in monthly rates of missed visits and medication availability (measured as percentage of days covered with ARVs). The ITS design allows for multiple observations over time before and after an intervention (i.e., implementation of the appointment system) and controls for most threats to internal validity (e.g., secular changes or population aging) because it adjusts for baseline trends in outcomes unrelated to the intervention.^^[[46]](#footnote-46)^^ We will use aggregate (by differencing monthly outcome rates between the intervention and control groups) and patient-level segmented linear regression models/generalized linear mixed models^^[[47]](#footnote-47)^,^^^[[48]](#footnote-48)^^ to estimate changes in both level and trend in study outcomes after the intervention, while controlling for autocorrelation and individual-level covariates using standardization for aggregate ITS.^^[[49]](#footnote-49)^^ These methods can also control for differential dropout through the use of additional covariates to account for changes in population characteristics each month.^^[[50]](#footnote-50)^^ The segmented regression models will include a constant term; an integer variable indicating number of months from the start of the observation period to estimate baseline trend; a binary variable—intervention—set to 1 for time periods after the month the appointment system was introduced to estimate change in level; and an interaction between intervention and time to estimate change in trend. We will adjust for first-order autocorrelation between sequential measurements and assess model fit using likelihood ratio statistics and Akaike information criterion.

An example of our aggregate time series model for the missed visit outcome is as follows:

% of visits missed= β _0_ + β_1_time + β_2_intervention + β_3_time after intervention + e

As illustrated in the equation above, the basic statistical model will include a constant term; an integer variable indicating time in months (t) from the start of the observation period; an indicator of the time period after intervention was implemented; and an interaction between policy and time. The coefficient associated with time (β_1_) estimates the baseline trend in the outcome measure from its level (β _0_) at the beginning of the baseline period. The coefficient associated with the policy indicator (β _2_) estimates the level change in the outcome in the period following intervention, controlling for baseline trend. The coefficient associated with the time after intervention variable (β_3_) estimates the change in trend (slope) following the intervention.

Time series models will control for autocorrelation by testing for autoregressive processes (serial correlation between monthly measures) and correcting for significant correlations, which is easily accommodated using the SAS Autoreg procedure.^[[51]](#footnote-51)^ We will use the Durbin-Watson statistic to test for serial autocorrelation of the error terms in the regression model (D-W statistic near 2.00 indicates no serious autocorrelation). Adjusting for significant first-order autoregressive parameters controls for correlation between consecutive observations, while adjusting for significant twelfth-order parameters controls for annual seasonality. To test assumptions of the linear model, we will examine residuals to determine if they are normally distributed and have no observable pattern over time. We will test models with and without outliers to assess their impact on results.

We will use Kaplan-Meier and accelerated failure time methods to assess the impact of appointment systems on 15-day gaps in visits and greater than 60-day loss to follow-up. We will use Kaplan-Meier survival curves to graphically depict time until these outcomes. We will use accelerated failure time models, allowing average failure time to be estimated and compared.^^[[52]](#footnote-52)^^ We will use a more advanced version of accelerated time models/Bayesian piece-wise exponential models^^[[53]](#footnote-53)^^ to adjust for correlation within person/facility by finding the best distribution for failure time using likelihood ratio tests. Because patients are at risk for repeated gaps in care, the denominator for post-intervention analyses will comprise all patients still in care at the time of the intervention. Our statistical comparison will be the times-to-event in the post-intervention period in intervention versus control patients, relative to the times-to-event in the baseline period in intervention versus controls. Controlling for covariates, we will calculate the relative ratios of average failure time in the follow-up period between the intervention and control cohorts and compare them with the relative ratios in the baseline period.

## 2.11 Policy impact

As described, the entire intervention will be in partnership with the MOHSW, particularly the PMTCT Unit. The PMTCT Unit works closely with the National AIDS Control Programme (NACP), which partnered with us on the previous INRUD-IAA project. Following the pilot study, the NACP adopted the appointment system for all ART clinics as policy, but has not yet enacted it. The MOHSW often relies on development partners to implement new policies such as this; partners thus need to understand the process and put it into their future work plans. As an important part of influencing policy, the PMTCT Unit has appointed a collaborator to work on the project and to be part of the team on all survey and implementation visits. Similarly, we will have one district health staff person accompany the teams on all visits. This engagement should ensure the MOHSW sense of ownership and correspondingly improve the chance of the intervention approach being widely implemented, if successful.

If this low-cost appointment and patient-tracking system in RCH clinics improves adherence and retention, there will be a strong motivation to scale-up the introduction nationwide. The MOHSW knows that adherence and retention are real problems with the newly adopted Option B-Plus program. We will also include other development partners and their locally based funding agencies in the stakeholder meetings at the beginning and end of the project. If the MOHSW adopts the appointment and patient-tracking system as policy, then the other partners will already be oriented on it. As such, this small project has a good chance of having a large policy impact.

# 3.0. ETHICAL ISSUES

All quantitative data will be extracted from routine patient records. All data collectors will treat all data gathered as confidential. There will be no direct patient involvement in the quantitative data collection. All data will be treated in strictest confidence and aggregated anonymously for all analysis and reporting.

All patients and staff will give formal consent to be interviewed and will have the right to stop at any time. All qualitative data will be anonymised.

To meet privacy concerns with how patients might be contacted when they miss appointments, part of the facility preparation exercise will be for staff to ask each patient if and how they would like to be contacted for follow-up. We will record the “refusal to be traced” rate, but it will not otherwise affect our evaluation.

If community health workers are part of the planned tracing process, they will be made very aware of ethical and privacy issues.

# 4.0 PROGRAM IMPLEMETATION AND WORKPLAN

We will carry out the intervention in partnership between PMTCT Unit of the MOHSW in the Reproductive and Child Health Section and MSH. The record review will be recorded anonymously and patients will not be involved directly. The overall PIs in Tanzania will be Dr Suleiman Kimatta (Country Representative of MSH Tanzania), Dr. Mwikemo Deborah Kajoka , (National PMTCT coordinator), and Mrs Salama Mwatawala (MSH and Main organizer of previous INRUD IAA project in Tanzania).

The two main local coordinators will be: Ms Levina Lema (PMTCT MOHSW) and Mr Jafary Liana (MSH) and the Qualitative Research Coordinator will be Dr Angel Dillip (Ifakara Health Institute)

Advisors will be overall Dr. John Chalker from MSH USA, and for the impact evaluation will be Professor Dennis Ross-Degnan and Dr Catherine Vialle-Valentin from the Department of Population Medicine at Harvard Medical School and Director of Research at Harvard Pilgrim Health Care Institute.

Two evaluation teams will conduct the impact evaluation. Each team will include three data collectors and a supervisor from MSH Tanzania with guidance from Harvard Pilgrim. Each team will also be accompanied by a qualitative data collector, Dr. Angel Dillip from the Ifakara Health Institute and her assistant. Mrs. Salama Mwatawala, who implemented the intervention in our previous adherence initiative, will train the intervention teams who will work directly with the facilities.

The Gantt chart and Table 3 below list all proposed project activities for the intervention and evaluation.

Table 3: Activities and time line for the proposed intervention

| **Date** | **Activity** | **Comment** |
| --- | --- | --- |
| December 2014 | | |
|  | Discussions with PMTCT Unit of MOHSW, |  |
|  | Visit to Mbeya for discussion with Regional Health team and Walter Reed Program, and reconnaissance trip to selected facilities to see appointment recording systems. |  |
| February 2015 | | |
| Week 2 | Submission of proposal to IRB of the National Institute for Medical Research |  |
| March 2015 | | |
| Week 1 | Permission from IRB |  |
| Week 2 | Stakeholder meeting |  |
| Week 3 | Preparation for Baseline Survey |  |
| Week 4 | Baseline Survey |  |
| April 2015 | | |
| Week 1 | Baseline Survey continued |  |
| Week 2-4 | Preliminary analysis of baseline survey data for giving feedback during the intervention |  |
| Weeks 2–4 | Preparation of intervention | Materials and clearances to visit facilities |
| May 2015 | | |
| Weeks 1–2 | Training of regional intervention teams | After baseline for impact evaluation |
| Weeks 3–4 | Intervention visits to each intervention facility | 2 teams of 3 for 2-day visit to each facility |
| June 2015 | | |
| Week 2 | Supportive supervision visits to each facility |  |
| Week 4 | Supportive supervision visits to each facility |  |
| July 2015 | | |
| Week 4 | Supportive supervision visits to each facility |  |
| August 2015 | | |
| Week 4 | Supportive supervision visits to each facility |  |
| January 2016 | | |
| Week 1-3 | Endline Survey |  |
| February–March 2016 | | |
| Weeks 1–6 | Final report writing |  |
| Week 8 | Final stakeholder meeting with JC in attendance |  |

December 2014–April 2015

During December, Mr. Liana and Dr. Kimatta from MSH Tanzania worked closely with the MOHSW, in particular the PMTCT Unit, and visited Mbeya to discuss the program with the regional health team and Walter Reed Program staff. They also looked at the recording systems in place in a selection of facilities.

In February 2015, we will prepare and submit the proposal to the National Institute for Medical Research ethical review board. With approval, we will convene a meeting of approximately 40 stakeholders in Dar es Salam to discuss the project. Stakeholders will include representatives from Mbeya region and key people from the MOHSW, Reproductive and Child Health Section, PMTCT Unit, NACP, WRP, and other regions’ support organizations. Stakeholder involvement and collaboration from the outset will facilitate not only this project, but future scale-up if successful.

In February, we will also develop the electronic survey tools, which will include purchasing six tablets and creating data capture programs for the quantitative research. MSH and Harvard recently did this for a large-scale data collection effort under a Gates-funded program. We will use those lessons learned to maximize effectiveness. Harvard will work with MSH Tanzania and Invention and Technological Ideas Development Organization, the local contractor we worked with previously, to develop, test, and finalize these data capture tools.

One of the lessons learnt in our large-scale survey was that data collectors needed careful training to familiarize them with using the tablet methodology. To ensure that this step goes well, a representative from Harvard will travel to Tanzania to supervise the training, which will be during the second week of March. The MSH Tanzania office will prepare for the baseline survey, which will take place over the last week of March and the first two weeks of April.

During April, Harvard will do a preliminary analysis of the quantitative baseline data and Dr. Dillip will process the qualitative data to provide the feedback information to the facilities during the intervention. They will also write up the results of the baseline survey.

May 2015

While the baseline survey is being carried out and the data analysed up to mid-May, we will prepare the materials and permissions for the intervention and identify the intervention team members. The baseline survey analysis will provide important information to inform the intervention. Salama Mwatawala, who helped introduce the appointment system in ART clinics under INRUD-IAA, will train the intervention teams. For the first two weeks of May, she will visit Mbeya and train two intervention teams to use the appointment and tracking system and also how to introduce it to the clinic staff. The training will last for two days. Introduction of the intervention itself will take place in the last two weeks of May. The intervention team will visit each of the 12 intervention clinics for two days to discuss adherence and retention issues with staff using baseline data; introduce the appointment system and patient-tracking system; and show how it enables facilities to better control their work schedules and load, rapidly identify missing patients and follow them up, and use the system to track treatment adherence performance using continuous quality improvement methods. As mentioned earlier, methods to follow up missing patients will vary by facility depending on local resources.

June–August 2015

In the second week of June, a team member will spend a half day in each clinic to provide supportive supervision and to help the clinic adapt to the system. The supervision team will return in the fourth week of June to help the clinic calculate their appointment-keeping indicators and discuss how they can make improvements. The team will then return once each in July and August to assure the system is working, get feedback from the clinic staff, and further track appointment-keeping progress.

November 2015–March 2016

Preparation for the endline assessment, which will occur seven months after the intervention, will begin in the second half of November as we adapt the baseline tools. In December, MSH Tanzania will prepare for the data collectors’ training, which will take place in the third week of December, facilitated by Mr. Liana and a representative from the Invention and Technological Ideas Development Organization.

Over the first two weeks of January, we will carry out the endline survey in intervention and control facilities. The two evaluation teams (one for each region) will be comprised of two data collectors and a supervisor (Mr. Liana or Mr. Valimba) and either Dr. Dillip or her assistant. The teams will spend two days in each of the 12 facilities. As with the baseline data, Harvard will analyse the quantitative data, and Dr. Dillip will analyse the qualitative data over the last two weeks of January and early February. Armed with these results, the evaluation team leaders will return to each intervention clinic share findings with the staff and get input on interpretations of the data.

After the final impact evaluation and data analysis, the intervention teams will visit each region in late February to share preliminary findings and discuss the results. At the end of March we will hold a dissemination meeting with stakeholders to share our experience, and if successful, plan for scale-up.

The specific activities for the impact evaluation are described by month below and also shown in the context of the whole project in the Gantt chart above.

# 5.0 STAFFING PLAN

Overall Principal Investigators

**Dr. Suleiman Kimatta** is currently the Country Representative for MSH Tanzania. In his position he coordinates projects implemented by MSH in Tanzania including the Sustainable Drug Seller Initiatives’ accredited drug dispensing outlet (ADDO) program. Dr. Kimatta served as Senior Program Associate providing oversight and technical input on the child health component of the ADDO program, including developing training, advocacy, and monitoring materials and tools. Dr. Kimatta also served as UNICEF National Project Officer–Health‐IMCI, Malaria, EPI and Nutrition; Regional Medical Officer for Mtwara, Zonal Paediatrician–Southern Zone; and Zonal CDD/ ARI Program Coordinator in the Southern Zone. He has also served in international technical assistance positions such as a World Health Organization (WHO) temporary advisor for inter-country IMCI courses in Arusha/ Tanzania; and WHO temporary advisor for intermediate and district level management of disease control programs (WHO/AFRO in Ghana and Ghana and Tanzania). Dr. Kimatta is a holder of Doctor of Medicine and Master Degree in Paediatrics from School of Medical Sciences, University of Santiago, Cuba.

**Dr. Mwikemo Deborah Kajoka** has 13 years of expertise and achievements in HIV and AIDS program design, planning and management, relationship management, business planning and strategy, health system strengthening, influencing policy change, and general medical practice. As the National PMTCT Coordinator since January 2011, she has been responsible for leading the country towards achieving the agenda of eliminating mother-to-child transmission of HIV and contributing to the national HIV and AIDS response.

**Salama Mwatawala** is a pharmacist with a Master’s Degree in Public Health from the University of Dar es Salaam. She is a Senior Technical Advisor for MSH Tanzania and has coordinated pharmaceutical management assessments and operational research initiatives including those related to pharmaceutical management information systems , pharmacovigilance, and neglected tropical diseases in Tanzania. She was the main implementer of the INRUD-IAA activities in Tanzania, where she introduced the appointment system to selected ART clinics and carried out most of the supportive supervision. She is currently working with the USAID-funded Systems for Improved Access to Pharmaceuticals and Services program, providing technical assistance to selected African countries to strengthen pharmaceutical management systems for tuberculosis. Ms. Mwatawala also supported the national TB program in Tanzania to pilot interventions engaging private drug outlets in increasing tuberculosis case finding.

**Overall Coordinators**

**Levina Lema** is a statistician who has been the Strategic Information Officer in the PMTCT Unit since 2007. Before that she was a researcher with the National Institute of Medical Research and has a wide range of experience in both quantitative and qualitative research.

**Jafary Liana** is a pharmacist with 15 years’ experience in both the public and private pharmaceutical sector in Tanzania. He is a Senior Technical Advisor for MSH Tanzania working as a local project lead for the Sustainable Drug Seller Initiatives program, which provides technical support to the Pharmacy Council of Tanzania on the maintenance and sustainability of the ADDO program. Mr. Liana has managed private sector programs related to access to essential medicines and pharmaceutical services, and he has successfully coordinated activities related to project planning, implementation, rapid assessments, and monitoring and evaluation of private sector medicine access initiatives. **Mr. Liana** will coordinate all activities in Tanzania, with technical guidance from Ms. Mwatawala and with technical and administrative support from the MSH Tanzania office. MSH’s Tanzania office will organize flights, vehicle hire, local meetings, and per diems . Jafary will ensure all that the in-country collaborations, permissions, invitations, and logistics are in place for both the evaluation and the intervention. He will also act as leader of the Mbeya evaluation team for the baseline and endline surveys and ensure the quality of the work throughout.

**Qualitative Research Coordinator**

**Angel Dillip, PhD** is a social epidemiologist with diverse academic and field experience in public health research. She has been involved in project design, management, and monitoring and evaluation in social and human development interventions, especially in community and rural development. For the past 10 years, Dr. Dillip has been immersed in public health-related social research focusing on social underpinnings of ill health, the social impacts of disease and illness, and epidemiological implications of social behaviour. She has a PhD in Public Health and Epidemiology from the University of Basel, Switzerland. She has been a Research Scientist with the Ifakara Health Institute since 2006, and since 2012, has worked with the data analysis cluster to provide expert consultancy on mixed methods analyses at the outset of project work. Under the guidance of MSH and Harvard Pilgrim, Dr. Dillip will lead the qualitative research as part of the impact evaluation to explore attitudes about adherence and about the intervention itself. She will recruit and manage an assistant to go to the second region and perform the interviews during the baseline and endline surveys. Dr. Dillip has worked with MSH several times and has produced high-quality qualitative research results.

**Advisors**

**John Chalker, MBchB, PhD** will advise on the overall coordination of the project’s activities. He will help the PIs ensure the timeliness and fidelity of the intervention and coordinate with the evaluation team. Dr. Chalker will regularly liaise with project staff through weekly conference calls to check on progress and plan next steps, and he will submit regular reports to 3ie. He will be backed up by Keith Johnson, who is the Global Technical Lead for Private Sector Initiatives at MSH. Dr. Chalker will also have the full technical and administrative support of MSH’s home office. Dr. Chalker is a UK-trained physician with a Diploma in Tropical Medicine and Hygiene, a Master’s degree in Community Health in Developing Countries, and a Doctorate in Health Systems Research. Based in England, he is a Principal Technical Advisor for MSH. He recently completed his role as the coordinator for INRUD-IAA, the five-year Swedish-funded program with the goal of monitoring and improving adherence to ART in East Africa. He has more than 25 years of international experience in designing, implementing, and managing health development projects and quality improvement interventions in a wide range of resource-poor countries in Africa, Asia, and the Middle East, including Yemen, Vietnam, and Burma.

**Impact Evaluation Advisors**

**Dennis Ross-Degnan, Sc.D**., is Associate Professor at the Department of Population Medicine at Harvard Medical School and Director of Research at Harvard Pilgrim Health Care Institute. He holds a doctorate in health policy and management from the Harvard School of Public Health. For over thirty years, Dr. Ross-Degnan’s work has focused on improving health systems in the United States and developing countries, including research on the behavioural and systems factors that determine use of medicines, impacts of health and pharmaceutical policies on utilization and clinical outcomes, interventions to improve quality of care, and appropriate methods for health and pharmaceutical systems research. He has a broad background in the design and evaluation of health systems interventions, with a focus on disparities in access to and outcomes of care. Dr. Ross-Degnan will be chief advisor for the impact evaluation. Harvard Pilgrim has a long record of evaluating the effect of interventions in resource poor settings and has collaborated with MSH for over 20 years. He and Dr. Vialle-Valentin will act as quality control for the data collection instruments, the electronic tablet-based data entry, and the data itself. They will also perform the quantitative analysis.

**Catherine Vialle-Valentin, MD, MPH**, is a Senior Research Associate in the Department of Population Medicine at Harvard Medical School and the Harvard Pilgrim Health Care Institute. Dr. Vialle-Valentin received her medical degree from the Claude Bernard School of Medicine in Lyon, France and her Master of Science degree in Health Care Management from the Harvard School of Public Health. She is an experienced health services researcher in the area of policy strategies to improve access to medicines in LMICs. She has collaborated extensively with the WHO Department of Essential Medicines and Pharmaceutical Policies in the development and implementation of methods to measure the effects of pharmaceutical policy interventions with a focus on household surveys and pharmaceutical country profiles. She has also worked recently with MSH to develop a multi-component assessment of the role of ADDOs in the pharmaceutical sector in Tanzania. She is a member of the WHO Technical Working Group on Good Governance in the Pharmaceutical Sector.

To ensure the impartiality of the evaluation, neither Harvard Pilgrim nor Dr. Dillip will be involved in the intervention. Potential bias is most likely to occur in the interviews after the intervention, but using an independent researcher should help eliminate that bias. The quantitative data collection teams will be led by the same MSH Tanzania staff members that will lead the intervention team because they are experienced in data collection exercises and can ensure good quality data collection. For the baseline survey, there will not be bias because intervention and control facilities will not be identified. For the endline survey, the quantitative data recorded are objective data from the patient records, so any systematic bias is unlikely. Analysis will be performed by Harvard staff who will not be involved in the intervention.

# 6.0 ORGANIZATIONAL QUALIFICATIONS

## 6.1 Management Sciences for Health

MSH has more than 40 years of experience in establishing, managing, and evaluating pharmaceutical sector activities in developing countries. Early work in the 1970s led to the recognition of the key role that pharmaceuticals play in the delivery of high-quality health care in developing countries. MSH provides technical assistance and training in pharmaceutical and commodity management worldwide and helps to build locally owned, planned, and implemented initiatives that are sustainable.

The key to MSH’s success in pharmaceutical management has been its focus on program development that is built on the findings of sound pharmaceutical sector assessments, along with its commitment to employ the best and most experienced staff and consultants available to do this work. We emphasize the development of local staff capacity. If international technical assistance is needed, we have about 700 staff members working out of our primary office in the Washington, D.C., area and other locations worldwide, as well as a roster of more than 50 expert consultants.

Technical Expertise and Approach

Our core competencies and the areas in which MSH provides long- and short-term technical assistance include—

- Pharmaceutical diagnostic assessments
- Design and use of pharmaceutical management indicators
- Diffusion of emerging technologies, such as new treatments and diagnostics
- Policy analysis and dialogue
- Procurement and inventory management
- Pharmaceutical management information systems (manual and computerized)
- Pharmaceutical services, including counselling, adherence, and retention in therapy
- Pharmaceutical regulation and product quality, including pharmacovigilance
- Finance and health reform
- Laboratory services
- Rational use of medicines, such as containing antimicrobial resistance
- Public-private initiatives, such as accreditation programs for retail drug outlets
- Capacity building and training, including development of training materials
- Information development and dissemination
- Applied research

MSH work­s in collaboration with the public and the pri­vate sectors, nongovernmental organizations, and donors to develop combina­tions of technical and managerial approaches for appropriate, cost-effective use of health care commodities. In addition, we work with poli­cy makers, managers, and health care provid­ers to strengthen pharmaceutical management sys­tems and improve access to and use of essential medicines and other health commodi­ties. MSH often partners with local organizations to build their capacity to carry out the work and to foster sustainability.

In all of our work, we base our goals for im­proving commodity management on the need to develop capacity within partner countries and to foster collaboration between countries and institutions within those countries. As a result, the key to many MSH project achievements has been the broad-based support from all stakeholders built through a participatory approach to project design and implementation. Our successes have come in part from our focus on making the projects the property of the institutions involved and not of MSH or of the donor funding the project. We take the strategies, interventions, and materials developed as part of our work, as well as the lessons learned, and disseminate them widely for potential adaptation and use in all resource-limited settings.

MSH places a high priority on developing partnerships and maintaining good working relationships with the many international agencies and donors sponsoring the work that it does—including the U.S. Agency for International Development, the WHO, the Pan American Health Organization, the United Nations Children’s Fund, the Swedish International Development Cooperation Agency, and the World Bank—and foundations such as the Bill & Melinda Gates Foundation and the Rockefeller Foundation. In addition, we often partner with other providers of technical assistance to leverage resources and bring together complementary experience and skills. Three examples of recent MSH projects that show our capacity to lead similar research and intervention efforts include INRUD-IAA and Sustainable Drug Sellers Initiatives, as follows.

**The International Network for the Rational Use of Drugs Initiative on Adherence to Antiretrovirals**

**Grant from Swedish International Development Cooperation Agency**

**May 2006–June 2012**

**Program Officer: Moa Bergman**

**Address: Valhallavägen 199, 105 25 Stockholm, SWEDEN**

**Email:** [**Moa.Bergman@sida.se**](mailto:Moa.Bergman@sida.se)

In 2006, MSH received a five-year grant from the Swedish International Development Cooperation Agency for INRUD-IAA. Since then, INRUD-IAA, in collaboration with national AIDS control programmes in Kenya, Rwanda, Tanzania, and Uganda, has made major contributions to measuring and understanding antiretroviral adherence and how patient adherence and retention relates to facility-level operations. Specifically, INRUD-IAA has—

- Documented inconsistency in approaches used by programs and facilities within a given country and across countries to measure ART adherence and retention.
- Developed and validated reliable indicators for monitoring adherence that are measured using routine data available in treatment centres. Of particular interest has been the development of reliable and validated indicators based on appointment keeping (actual indicators being the monthly percentages of patients who attended clinic: on or before the day of their next scheduled appointment or within three days of their scheduled appointment).
- Documented that adherence rates differ substantially between facilities.
- Conceptualized and tested practical and scalable health system interventions to improve adherence and retention.

Successful interventions were implemented and assessed in Kenya, Rwanda Tanzania, and Uganda. They all included development of an appointment register to fix negotiated appointments, monitor appointment keeping, and reach out in a timely way to patients who miss appointments. In Tanzania, Rwanda, and Kenya, the national AIDS control programmes have decided to scale-up nationwide the use of standardized appointment registers and patient tracking registers. In Rwanda, the attendance indicators were added to the national performance-based financing program.

INRUD-IAA contributed a number of articles to the literature on adherence to ART and chronic care:

Chalker J. Wagner A, Tomson G, Laing R, Johnson K, Wahlstrom R, and Ross-Degnan D, on behalf of INRUD-IAA. Appointment systems are essential for improving chronic disease care in resource-poor settings: learning from experiences with HIV patients in Africa. **International Health** 2013**,** 5 (3): 163-165. <http://inthealth.oxfordjournals.org/content/early/2013/07/11/inthealth.iht013.full.pdf?keytype=ref&ijkey=Tjcx3IpChKATyP1>

Boruett P, Kagai D, Njogo S, Nguhiu P, Awuor C, Gitau L, Chalker J, Ross-Degnan D, Wahlström R, Tomson G, on behalf of INRUD-IAA. Facility-level intervention to improve attendance and adherence among patients on anti-retroviral treatment in Kenya’s quasi-experimental study using time series analysis. **BMC Health Services Research** 2013, 13:242. <http://www.biomedcentral.com/1472-6963/13/242>

Mwatawala S, Sand D, Malele R, Moshiro C, Senyael B, Somi G, Maselle A, Chalker J, & Ross-Degnan D. Strengthening the appointment and tracking systems for patients on antiretroviral therapy in Tanzania: A optimizing adherence to ART as part of people-centered public health. **International Journal of Person Centered Medicine** 2012, 2(4):825-836. <http://www.ijpcm.org/index.php/IJPCM/article/view/302>

Gusdal AK, Obua C, Andualem T, Wahlström R, Chalker J, Fochsen G, on behalf of the INRUD-IAA project. Peer counselor’s role in supporting patients’ adherence to ART in Ethiopia and Uganda. **AIDS Care**, June 2011, 23:6, 657-662

Obua C, Gusdal A, Waako P, Chalker J, Tomson G, Wahlström R, and The INRUD-IAA Team. Multiple ART Programs Create a Dilemma for Providers to Monitor ARV Adherence in Uganda. **The Open AIDS Journal** 2011, 5, 17-24. [http://www.benthams cience.com/open/toaidj/articles/V005/17TOAIDJ.pdf](http://www.benthamscience.com/open/toaidj/articles/V005/17TOAIDJ.pdf)

Chalker J. Wagner A, Tomson G, Laing R, Johnson K, Wahlstrom R, and Ross-Degnan D, on behalf of INRUD-IAA. Urgent need for coordination in adopting standardized antiretroviral adherence performance indicators. **Journal of Acquired Immune Deficiency Syndromes** 2010, 53(2):159-161**,** <http://journals.lww.com/jaids/Fulltext/2010/02010/Urgent_Need_for_Coordination_in_Adopting.1.aspx>

Chalker J, Andualem T, Gitau L, Ntaganira J, Obua C, Tadeg H, Waako P, Ross-Degnan D. Measuring adherence to antiretroviral treatment in resource-poor settings: The feasibility of collecting routine data for key indicators. **BMC Health Services Research** 2010, **10**:43. <http://www.biomedcentral.com/1472-6963/10/43>

Ross-Degnan D, Pierre-Jacques M, Zhang F, Tadeg H, Gitau L, Ntaganira J, Balikuddembe R, Chalker J, Wagner A. Measuring adherence to antiretroviral treatment in resource-poor settings: The clinical validity of key indicators. **BMC Health Services Research** 2010, **10**:42. <http://www.biomedcentral.com/1472-6963/10/42>

Chalker J, Andualem T, Tadeg H, Gitau L, Ntaganira J, Obua C, and Waako P.. Developing standard methods to monitor adherence to antiretroviral medicines and treatment defaulting in resource-poor settings: INRUD-IAA. **WHO** **Essential Medicines Monitor** 2009, 1:3-8. <http://www.who.int/medicines/publications/monitor/EMM_art3Issue_1_2009.pdf>

Gusdal AK, Obua C, Andualem T, Wahlström R, Tomson G, Peterson S, Ekström AM, Thorson A, Chalker J, Fochsen G, on behalf of the INRUD-IAA project. Voices on adherence to ART in Ethiopia and Uganda: A matter of choice or simply not an option? **AIDS Care** 2009, [21](http://www.informaworld.com.ezp-prod1.hul.harvard.edu/smpp/title~db=all~content=t713403300~tab=issueslist~branches=21#v21) ([11](http://www.informaworld.com.ezp-prod1.hul.harvard.edu/smpp/title~db=all~content=g916270514)):1381-1387.

Chalker J, Andualem T, Minzi O, Ntaganira J, Ojoo A, Waako P, Ross-Degnan D. Monitoring Adherence and Defaulting for Antiretroviral Therapy in 5 East African Countries: An Urgent Need for Standards; **Journal of the International Association of Physicians in AIDS Care** 2008, 7 (4): 193-199.

**Sustainable Drug Seller Initiatives (SDSI)**

**Grant from: Bill & Melinda Gates Foundation, P.O. Box 23350, Seattle, WA, USA**

**April 2011–December 2014**

**Program Officer: Mara Hansen**

**Email:** [**mara.hansen@gatesfoundation.org**](mailto:mara.hansen@gatesfoundation.org)

**Phone: +1 206 709 3357**

This program shows MSH’s ability to work in close collaboration with government agencies for many years to take interventions to scale. SDSI built on the successes of MSH’s Strategies for Enhancing Access to Medicines program, which began in 2000, and East African Drug Seller Initiatives program, which created and implemented public-private partnerships using government accreditation to increase access to quality pharmaceutical products and services in underserved areas of Tanzania and Uganda. In turn, SDSI ensured the maintenance and sustainability of the drug seller initiatives in Tanzania and Uganda and introduced the initiative in Liberia. SDSI also strengthened the adaptability of the initiatives in the face of changing health needs and local system context; facilitated the institutionalization of the initiatives through partnerships with local health training institutions; and produced data related to consumer access to and use of medicines and to inform public health policy, regulatory standards, and treatment guidelines. Through MSH’s work in the three countries, SDSI not only expanded access to medicines and treatment to additional geographical areas, but also solidified the global view that initiatives to strengthen the quality of pharmaceutical products and services provided by private sector drug sellers are feasible, effective, and sustainable in varied settings.

The ADDO initiative in Tanzania has now been scaled up nationwide, covering all of Tanzania’s 25 mainland regions. More than 9,200 accredited shops are open—approximately 7,000 accredited since 2011. Over 13,000 ADDO dispensers have been trained and licensed. In Uganda, Tanzania’s ADDO model was adapted to address the country’s needs. MSH and partners implemented the adapted program in five Ugandan districts with 569 accredited drug shops opened and over 1,100 shop attendants trained. Following a 2013 launch of the initiative in Liberia, 120 shops and 358 dispensers were accredited and licensed. In all three countries, approximately 13.5 million people benefit from improved access to quality medicines and care.

## 6.2 Harvard Pilgrim Health Care Institute

The Drug Policy Research Group (DPRG), in the Department of Population Medicine at Harvard Medical School and the Harvard Pilgrim Health Care Institute has over three decades of experience in applied research, providing technical support, and training on improving access to and use of medicines in the United States and in low- and middle-income countries. The DPRG has worked extensively with MSH and WHO at global, regional, and national levels and with many other international partners. The DPRG’s goal is to ensure financial and physical access to safe, effective, and high-quality medicines used appropriately and efficiently within health systems .

Key DPRG international activities include the following—

- Developing practical, reliable, and valid tools to measure medicines use, monitor medicines situations, and evaluate interventions, including country pharmaceutical sector assessments, health facility surveys, and household surveys
- Developing, implementing, evaluating, and disseminating innovative approaches to improve medicines policies, systems , and individual behaviours in collaboration with ministries of health, health delivery organizations, and communities
- Applying health systems research and evaluating programs to provide evidence for policymaking
- Conducting basic and advanced training in measuring medicines use, intervention methods, pharmaceutical systems development, and pharmaceutical policy analysis

The DPRG has partnered with the Tanzania MOHSW, INRUD/Tanzania, MSH Tanzania, and other local partners for many years. DPRG faculty have provided support to a household survey of medicines access and use, medicines price surveys and monitoring approaches in the public and private sectors, and development and evaluation of the INRUD-IAA intervention to improve access to medicines for patients with HIV/AIDS. The DPRG’s strengths include: (1) extensive experience working in Africa, Southeast Asia, East Asia, and Latin America; (2) high-level skills in evaluating policy, behavioural, and system change interventions; (3) implementing innovative, effective methods to build capacity in the pharmaceutical sector; and (4) building productive, lasting collaborations with international, regional, and country organizations on global and local medicines initiatives.

DPRG led the impact evaluations on **INRUD-IAA** and the **Sustainable Drug Seller Initiatives**.

# 7.0 BUDGET AND BUDGET JUSTIFICATION

|  | **February 2015 to May 2016** | | | | |  |
| --- | --- | --- | --- | --- | --- | --- |
|  |  |  |  |  |  |  |
|  | **Q1** | **Q2** | **Q3** | **Q4** | **Q5** | **TOTAL** |
| Salaries and Wages | $ 21,682 | $ 31,159 | $ 5,617 | $ 6,378 | $ 33,707 | **$ 98,543** |
| Travel and Transportation (local) | $ 2,530 | $ 49,020 | $ 11,040 | $ - | $ 27,480 | **$ 90,070** |
| Travel and Transportation (International) | $ 6,833 | $ - | $ - | $ - | $ 3,222 | **$ 10,055** |
| Sub-contracts | $ 23,909 | $ 14,795 | $ 10,150 | $ 7,753 | $ 16,027 | **$ 72,634** |
| Other Direct Costs | $ 14,664 | $ 4,120 | $ 420 | $ 1,686 | $ 12,770 | **$ 33,660** |
| Indirect costs | $ 7,278 | $ 12,929 | $ 2,765 | $ 1,349 | $ 11,871 | **$ 36,191** |
|  |  |  |  |  |  |  |
| **TOTAL** | **$ 76,896** | **$ 112,023** | **$ 29,992** | **$ 17,165** | **$ 105,077** | **$ 341,153** |

1. **Salaries and Wages**

Salaries and Wages include Tanzanian (local) and US-based MSH staff salaries as well as local and US-based staff benefits.

- **Local professional and local support staff -** MSH Tanzania technical professional and local support staff are budgeted with the work-days necessary for implementing all aspects of the project, including overseeing local partners’ work, and project monitoring and evaluation.
  - Local professional staff: Jafary Liana, Richard Valimba, Salama Mwatawala
  - Local support staff: Pamela Lema
- **US-based technical staff and home office support-** US-based MSH Center for Pharmaceutical Management staff will assist in developing technical approach and will provide technical oversight and remote technical and operational assistance, as needed.
  - US-based technical staff: John Chalker, Martha Embrey, Rachel Lieber, Keith Johnson
  - US-based home office support: TBD finance and contracts staff
- **Benefits-** Local and U.S. staff benefits are budgeted in accordance with local law and practice and MSH’s standard package of benefits for each country based on guidance from MSH’s Office of Human Resources. Tanzanian Local Payroll benefits are based on MSH’s obligation to meet the legally-mandated employee benefits requirements of Tanzania.

1. **Travel and Transportation (local)**

Local travel and transportation cost are included to facilitate the implementation and evaluation of the technical activities in Tanzania. Transportation costs are included for MSH, sub-contractor, data collector and key partner travel within Tanzania. Costs include travel for initial site visits to Mbeya, survey teams (baseline and endline), Mbeya intervention implementation teams , and supportive supervision teams . Travel costs include per diem, vehicle hire, and flights from Dar es Salaam to Mbeya.

1. **Travel and Transportation (international)**

Three international trips are budgeted for this project:

- Dr. John Chalker to participate in the initial stakeholders meeting.
- Dr. Dennis Ross-Degnan to train baseline survey data collectors.
- Dr. Chalker to participate in the final stakeholder dissemination meeting.

International travel costs include airfare, per diem, visa purchase, and airport transfer.

1. **Sub-contracts**

MSH will issue sub-contracts to two partners to help facilitate the study:

- Harvard Pilgrim Health Care - Harvard Pilgrim, led by Dr. Dennis Ross-Degnan, will lead the impact evaluation. Harvard Pilgrim has a long record of evaluating the effect of interventions in resource poor settings and have collaborated with MSH’s Center for Pharmaceutical Management for over 20 years. They will act as quality control for the data collection instruments, the electronic data entry programs , and the data itself. They will also perform the quantitative analysis. HPHC’s budget includes staff time for Dr. Ross-Degnan, Catherine Vialle-Valentin (senior researcher), and Ann Maas (grants administrator). The budget also includes an approved indirect cost rate.
- Apotheker Consultancy, Ltd. - Apotheker will provide qualitative research scientist, Dr. Angel Dillip, to lead the qualitative research as part of the impact evaluation to explore attitudes about adherence and about the intervention itself. Dr. Dillip will perform qualitative interviews in half of the Mbeya facilities during the baseline and endline surveys. Dr. Dillip will train the qualitative data collector who will complete the second half of the qualitative interviews during the baseline and endline surveys. Dr. Dillip has worked with MSH several times and has produced high-quality qualitative research results. Apotheker will also engage a regional consultant for Mbeya to carry out the intervention and supervision activities in conjunction with MSH. Apotheker’s budget includes staff time for Dr. Angel Dillip, Dr. Romuald Mbwasi (technical oversight), and a TBD local consultant.

1. **Other Direct Costs**

Other direct costs include trainings, meetings, supplies, and outside services.

- Trainings: Data collector training before baseline and endline surveys.
- Meetings: Initial stakeholders meeting and final stakeholder dissemination meeting.
- Supplies: Communications, printing, postage, fees for open access journals, and electronic data collection tools.
- Outside Services: Data collectors for baseline and endline surveys, and local company to program the electronic data collection tool.

1. **Indirect Costs**

As a Non-Governmental Organization, MSH strives to keep indirect costs as low as possible. For the purpose of this grant proposal, MSH has complied with the donor’s indirect cost allowance rate.

# 8.0 DATA COLLECTION FORMS

## 8.1 Quantitative Forms in English

The data will be collected on hand-held tablets and is presented in the tables below. The facility table is filled in once during the baseline and follow-up visit. The patient table is filled at baseline from the clinic form known as the CTC-2 form.  The visit table would contain all of the useful data from the CTC-2 form for each patient visit.  For the patient and visit table there is an indication of where the data appear on the CTC-2 form.

### 8.1.1 Facility Form (1 per facility)

| **Field name** | **CTC2 location** | **Field type** | **Index** | **Description** |
| --- | --- | --- | --- | --- |
| Facility name | in header | alpha |  | select from facilities listed at right |
| Facility ID |  | numeric | Y | see table at right (automatically filled) |
| District name |  | alpha |  | see table at right (automatically filled) |
| Name of in-charge |  | alpha |  | name of facility in-charge |
| Name of data contact |  | alpha |  | name of local person assisting in data collection |
| Contact number |  | numeric |  | telephone number of local contact person |
| Baseline data collection date |  | dd/mm/yy |  | date of team visit during baseline survey |
| Baseline data collection team number |  | numeric |  | team number |
| Baseline data earliest date recorded |  | dd/mm/yy |  | earliest date of baseline data collected |
| Baseline data latest date recorded |  | dd/mm/yy |  | last date of last baseline data collected |
| Number ART patients on baseline survey date |  | numeric |  | number of active patients on day of data collection |
| Number of ART patients in month of earliest date |  | numeric |  | number of active patients in month of earliest baseline data |
| Follow-up data collection date |  | dd/mm/yy |  | date of team visit during follow-up survey |
| Follow-up data collection team |  | numeric |  | team number |
| Follow-up data earliest date recorded |  | dd/mm/yy |  | earliest date of follow-up data collected |
| Follow-up data latest date recorded |  | dd/mm/yy |  | last date of follow-up data collected |
| Number of ART patients on follow-up survey date |  | numeric |  | number of active patients on day of follow-up data collection |
| Usual number of ARV days dispensed (initial) |  | numeric |  | usual number of ARV days dispensed in initial prescription (dispenser report) |
| Usual number of ARV days dispensed (second) |  | numeric |  | usual number of ARV days dispensed in second prescription (dispenser report) |
| Usual number of ARV days dispensed (continuing) |  | numeric |  | usual number of ARV days dispensed in continuing prescription (dispenser report) |

### 8.1.2 Patient Form (1 per patient)

| **Field name** | **CTC2 location** | **Field type** | **Index** | **Description** |
| --- | --- | --- | --- | --- |
| Facility ID |  | numeric | Y | automatically pre-filled from facility table |
| Study ID |  | numeric | Y | sequential ID assigned by facility (start each facility with 101) |
| Unique CTC ID number | in header | alphanumeric |  | ID used in facility records |
| Patient name | in header | alpha |  | name as it appears in facility records |
| Birth date | in header | dd/mm/yy |  | if available, otherwise blank |
| Age | in header | numeric |  | years if available, otherwise blank |
| Marital status | in header | code |  | see list (pick 1) |
| Number of children | On back of form | numeric |  | number if available, otherwise blank |
| Referred from | in header | code |  | see list (pick 1) |
| Date confirmed HIV | in header | code |  | if available, otherwise blank |
| Date enrolled in care | in header | dd/mm/yy |  | if available, otherwise blank |
| Date medically eligible | in header | dd/mm/yy |  | if available, otherwise blank |
| Date eligible and ready | in header | dd/mm/yy |  | if available, otherwise blank |
| Date start ART | in header | dd/mm/yy |  | if available, otherwise blank |
| Age at start | in header | numeric |  | if available, otherwise blank |
| WHO stage at start | in header | numeric |  | if available, otherwise blank |
| CD4 count/% at start | in header | numeric |  | if available, otherwise blank |
| Body weight at start | in header | numeric |  | if available, otherwise blank |

### 8.1.3 Visit Form (1 per visit)

| **Field name** | **CTC2 location** | **Field type** | **Index** | **Description** |
| --- | --- | --- | --- | --- |
| Study ID |  | numeric | Y | unique ID assigned consecutively by facility ID (e.g. 101.001, 101.002, etc.) |
| Visit ID |  | numeric | Y | automatically assigned consecutively by unique patient |
| Visit date | 1 | dd/mm/yy |  | date of current visit |
| Visit type | 2 | text |  | see code 2 (pick one) |
| Weight | 3 | numeric |  | weight in kgs |
| WHO stage | 5 | numeric |  | stage 1-4 |
| CD4 count | 6 | numeric |  | CD4 count/% |
| Signs and symptoms | 7 | text |  | see code 3 (check all that apply) |
| ARV reason | 13 | numeric |  | see code 9 (pick 1) |
| ARV regimen | 14 | text |  | see code 10 (pick 1, enter code only) |
| ARV days dispensed | 14 | numeric |  | enter if present (or enter usual days dispensed in this facility) |
| ARV adherence | 15 | text |  | see code 11a (pick 1) |
| ARV adherence poor reason | 15 | numeric |  | see code 11b (pick 1 only if previous item=P) |
| OI treatment | 16 | numeric |  | see code 12 (check all that apply) |
| Next visit date | 23 | dd/mm/yy |  | date of next scheduled visit (must be greater than current date) |
| Follow-up status | 24 | text |  | see code 16 (pick 1) |
| Discussed adherence this date | on back | text |  | Y/N (default to N) |
| Comments about adherence | on back | text |  | add comments (only if previous item=Y) |
| Discussed appointments this date | on back | text |  | Y/N (default to N) |
| Comments about appointments | on back | text |  | add comments (only if previous item=Y) |

## 8.2 Interview guidelines in English

### 8.2.1 District Health Officials interview guidelines

**TOOL 1: SEMI-STRUCTURED INTERVIEW GUIDE FOR DISTRICT HEALTH OFFICIALS (DMO, RCH Coordinator, HIV/AIDS coordinator)**

***For Intervention Districts at Baseline and Control Districts at Endline. For Intervention Districts at Endline use questionnaire at the end***

(The interview will take place after signing the consent form, Respondents will answer questions in their own words and the interviewer will probe for elaboration.)

District____________Ward________________

1. How long have you been working in this district?
2. What is your position and how long have you been working in the said position?
3. When did health facilities in this district start providing HIV care and treatment in Reproductive and Child Health (RCH) clinics? (Probe on how many villages/wards are served by the clinic)
4. How did the district and health care providers respond to the integration of HIV care and treatment in RCH clinics?
5. How did the community respond to the integration of HIV care and treatment in RCH clinics?

**ADHERENCE, RETENTION AND PATIENT ENGAGEMENT WITH CARE (Specific for RCH Clinics, for all these questions, adherence and retention will be asked separately)**

1. Do you think that women with HIV at RCH clinics on ART have problems with adherence? (probe on general perception of the problem)
2. Why do you think that these women have problems with adherence in your district?

Probe for

- 1. Individual factors i.e. behavioural, structural and psychological factors (life style factors, poverty, depression
  2. Health system factors i.e. Patient-provider relationship, confidentiality, unavailability of drugs
  3. Community factors i.e. Stigma, un/availability of social support

1. Do problems at RCH facilities potentially contribute to poor adherence and retention to care?
   1. Which kinds of problems?
   2. Why do you think that is the case?
2. Are there programs in place to improve adherence to ART in this district? (If yes please explain)
3. What are the existing programs at RCH clinics to support patient adherence? Probe for
   1. Existing programs at RCH facilities and community and how they function
   2. Involvement of any HBC and how they function to improve adherence
4. How do RCH facilities assess adherence among clients? (probe for methods used to monitor adherence i.e. self-reports, pill count)
5. What challenges do RCH facilities face in monitoring adherence among patients?
6. What adherence information is reported to the district (probe on how it is used)
7. How do the RCH facilities use adherence information? (Probe if it used for any intervention)
8. What do you think could be done to improve adherence and retention to ART for women at RCH clinics? (probe on potential interventions at both individual, health system and community levels)
9. Does the district have any programs to support adherence and retention to ART at RCH clinics? (probe on the district support to respective health facility, challenges faced)

**PATIENT APPOINTMENTS AND FOLLOW-UPS**

1. What is the current system of making appointments for patients and how is it confirmed whether they have attended?

Probe on

a) Do providers ask if the proposed day is convenient for the patient?

b) How do providers recognize patients who miss their appointment?

1. How do providers follow up patients who miss their appointment and challenges involved? (Probe if they call them/no follow-ups done?, what role does the district play in this)
2. What relationships exist between health facilities and community programs? (Do facilities liaise with any existing local resources in tracking/following up patients (probe on use of HIV/AIDS related HBC i.e. community HIV support program/organization, CHW). If not why?
3. Do you think it is important to liaise with any existing local resources in tracking/following up patients who miss appointment? (Why?)
4. What other/potential local resources do you think are important to liaise with in following up patients (for any mentioned resource ask the reason)
5. What do you think would be a more acceptable way of following up patients who miss appointments?

***End with question 23 for baseline in intervention districts.***

1. Is there anything else you would like to share or add regarding patients’ adherence and retention to care in this district?

***For endline in control districts continue with questions 24-26***

1. Have you heard of appointment and tracking system that has been introduced in some health facilities in this district? (If yes, probe on when did they hear of the intervention, what do they know about the purpose of intervention, what do they think are the advantages and disadvantages of the intervention)
2. For the past eight months, has there been any change in the way you arrange for appointment and tracking missing patients? (probe for any intervention that took place in the past eight months, policy change related to appointment and tracking missing patients)
3. Is there anything else you would like to share or add regarding patients’ adherence and retention to care in this district?

Thank you for participating in the study.

**Endline question guide for intervention district health officials**

1. How long have you been working in this district?
2. What is your position and how long have you been working in the said position?
3. Do you think that women with HIV at RCH clinics on ART have problems with adherence? (probe on general perception of the problem)
4. Why do you think that these women have problems with adherence in your district?

Probe for

- 1. Individual factors i.e. behavioural, structural and psychological factors (life style factors, poverty, depression
  2. Health system factors i.e. Patient-provider relationship, confidentiality, unavailability of drugs
  3. Community factors i.e. Stigma, un/availability of social support

1. Does your district have any programs to help patients with HIV treat their condition effectively?
2. Are you aware of the patient appointment and tracking system introduced at RCH clinic in this district? (Probe on when it was introduced, by whom and for what purpose)
3. How does the new tracking and appointment system differ from the old one (Probe on how the new system work compared to the old one)
4. Using the new system, how does the health provider make appointments for patients and confirm whether they have attended?

Probe on

- 1. Do they ask if the proposed day is convenient for the patient? (any negotiating done with regard to date and time)
  2. What time of the day are patient appointments arranged? (in the morning/afternoon/evening)
  3. How do they recognize patients who miss their appointment?

1. Using new system, how often do health providers track attendance and missed appointments for women on ART? (Probe on how frequent is attendance and missed appointments tracking done i.e. daily, weekly, monthly, how feasible is it and any challenges involved)
2. How do health providers track/follow women who miss their appointment? (Probe on the use of local resources i.e. HBC, CBOs, phone calls, fellow patients) and how did they decide on local resources to work with?
3. What relationships does RCH clinics have with community organizations/outreach programs (HBC, CHW, Community HIV support program and other local organizations, how do they work together in tracking women on ART? (Probe on the names of organizations, their roles, benefits and challenges involved in working with them).
4. Do you think the new system of appointment and tracking has improved adherence and retention among women to ART? (If yes why? Probe how they see improvements in adherence compared to the past, do many women appear on their appointment day compared to past? Why?)
5. Do you think the new system has contributed to improved health facility performance in this district (i.e. clients flow/timeliness of treatment, availability of drugs, treatment confidentiality, patient-provider interaction)
6. How do you like the new system of appointment and tracking compared to the old one? (Probe on the advantages and disadvantages of each, i.e. how well is the new system working, problems in implementing it, and how to overcome challenges)
7. What was the response of community/women on ART following the introduction of the new system? (Probe on the uptake of the system by women and whether they like the system or not and reasons behind each response, probe on issues on perceived stigma after using community organizations)
8. Do you think the new system of patient appointment and tracking should continue and be introduced to other facilities? (If yes why, if no why)
9. Has there been any support from district to facility level in implementing the new system? (If yes what support, if no why?)
10. What else do you think could be done to improve the new system of patient appointment and tracking to increase adherence and retention to ART? (probe on potential interventions at both individual, health system and community levels)
11. Is there anything else you would like to share or add regarding patients’ appointment system and adherence/retention to care?

Thank you for participating in the study.

### 8.2.2 RCH Clinic Staff interview guidelines

**TOOL 2: INTERVIEW GUIDE FOR CLINIC STAFF (RCH)**

*For use for Intervention clinic staff at Baseline and Control clinic staff at Endline. The Endline intervention clinic staff interview guidelines are at the end*

(The interview will take place after signing the consent form, Respondents will answer questions in their own words and the interviewer will probe for elaboration.)

Name of health facility_______________District____________Ward________________-

Type of facility (circle): a) Public b) Private c) Faith Based

Level of facility (circle): a) Hospital, b) Health Center c) Dispensary

1. How long have you been working (Probe on a) in providing health care services in general b) at this facility and c) at providing HIV and AIDS services? d) at providing HIV and AIDS services in this facility?
2. When did this facility start providing HIV care and treatment at MCH clinics? (Probe on how many villages/wards are served by the clinic)
3. How have clinic staff responded to the integration of HIV care and treatment in MCH clinic?
4. How have community/patients responded to the integration of HIV care and treatment in MCH clinic?

**ADHERENCE, RETENTION AND PATIENT ENGAGEMENT WITH CARE (For all these questions, adherence and retention will be asked separately)**

1. What do you understand by adherence? ( probe the understanding of adherence as opposed to retention)
2. Do you assess adherence amongst your clients? If so how do you do it? (probe for methods used to monitor adherence i.e. self-reports, pill count)
3. What challenges do you face in monitoring adherence among your patients? How does the facility use adherence information? (Probe if it used for any intervention)
4. Does this facility have problems with supporting patients in adhering to ART and retaining patients in care? (probe on general perception of the problem)
5. What problems do you think the clients have with regard to poor adherence in this facility?

Probe for

- 1. Individual factors i.e. behavioural, structural and psychological factors (life style factors, poverty, depression
  2. Health system factors i.e. Patient-provider relationship, confidentiality, unavailability of drugs
  3. Community factors i.e. Stigma, un/availability of social support

1. Are there challenges faced by this MCH facility that could potentially be contributing to poor adherence and retention to care?
2. Have you put into place any strategies to improve adherence to ART and retention to care in this facility?
   1. Probe: Could you describe them? When did they begin? Why did you implement them?
3. What else do you think could be done to improve client adherence and retention to ART? (probe on potential interventions at both individual, health system and community levels)

**PATIENT APPOINTMENTS AND FOLLOW-UPS**

1. What is the current system of making appointments for patients on ART to come back for follow-up care?
2. Do you have a way of knowing if patients have attended?

(Ask to see the register and how it works)

Probe on

1. Do you ask if the proposed day is convenient for the patient?
2. How do you recognize patients who miss their appointment? (Probe on when do they recognize)
3. Do you have a system to follow up patients who miss their appointment at home?
   1. If yes, are there challenges involved? And if so what are some of the challenges? (Probe if they call them/no follow-ups done?)
4. Do you liaise with any community programs in following up patients?
   1. (probe on use of HIV/AIDS related HBC i.e. community HIV support program/organization, CHW). If not why?
5. Do you think it is important to liaise with community programs in tracking/following up patients who miss appointments?
   1. (Why? perceived advantages and disadvantages)
   2. How could you follow up with patients who miss appointment in a way that would be more accepted by the clients?

***(End with question 18 if it is baseline in intervention facilities, continue with questions 19-21 for endline in control facilities)***

1. Is there anything else you would like to share or add regarding patients’ adherence/retention to care and appointment system?

Thank you for participating in the study.

1. Have you heard of appointment and tracking system that has been introduced in some health facilities in this district? (If yes, probe on when did they hear of the intervention, what do they know about the purpose of intervention, what do they think are the advantages and disadvantages of the intervention)
2. For the past eight months, has there been any change in the way you arrange for appointment and tracking missing patients? (probe for any intervention that took place in the past eight months, policy change related to appointment and tracking missing patients)
3. Is there anything else you would like to share or add regarding patients’ adherence/retention to care and appointment system?

Thank you for participating in the study.

**Question Guide for interviewing facility staff at Endline in intervention facilities**

1. Name of facility________________District________________Ward_______________
2. Level of facility (circle) a) Hospital b) Health Centre c) Dispensary
3. Type of facility (circle): a) Public b) Private c) Faith Based
4. How long have you been working (Probe on a) in providing health care services in general b) at this facility and c) at providing HIV and AIDS services? d) at providing HIV and AIDS services in this facility?
5. Does this facility have problems with supporting patients in adhering to ART and retaining patients in care? (probe on general perception of the problem)
6. What problems do you think the clients have with regard to poor adherence in this facility?

Probe for

- 1. Individual factors i.e. behavioural, structural and psychological factors (life style factors, poverty, depression
  2. Health system factors i.e. Patient-provider relationship, confidentiality, unavailability of drugs
  3. Community factors i.e. Stigma, un/availability of social support

1. Does your clinic have any programs to help patients with HIV take their medicines more consistently?
2. Are you aware of the patient appointment and tracking system introduced at MCH clinic in this facility? (Probe on when was it introduced, by whom and for what purpose)
3. How does the new tracking and appointment system differ from the old one (Probe on how the new system work compared to the old one)
4. Using the new system, how do you make appointments for patients and confirm whether they have attended? (Ask to see the appointment registers and ask how it works) Probe on
   1. Do you ask if the proposed day is convenient for the patient? (any negotiating done with regard to date and time)
   2. What time of the day are patient appointments arranged? (in the morning/afternoon/evening)
   3. How do you recognize patients who miss their appointment?
5. Using the new system, how often do you track attendance and missed appointments for women on ART? (Probe on how frequent is attendance and missed appointments tracking done i.e. daily, weekly, monthly, how feasible is it and any challenges involved)
6. How do you track/follow women who miss their appointment? (Probe on the use of local resources i.e. HBC, CBOs, phone calls, fellow patients) and how did you decide which local resources to work with?
7. What are the common reasons mentioned by women who miss their appointment for one to three days?
8. What community organizations/outreach programs (HBC, CHW, Community HIV support program and other local organizations) are you working with in tracking women on ART? (Probe on the names of organizations, their roles, benefits and challenges involved in working with them)
9. Do you assess adherence amongst your clients? If so how do you do it? (Probe for methods used to monitor adherence using the new system, benefits and challenges, what is done in case of poor adherence .i.e. monthly meetings to discuss progress?)
10. Do you think the new system of appointment and tracking has improved adherence and retention among women to ART? (If yes why? Probe how they see improvements in adherence compared to the past, do many women appear on their appointment day compared to past? Why?)
11. Do you think the new system has addressed health system challenges in this facility? Has it improved things? (i.e. clients flow/timeliness of treatment, availability of drugs, treatment confidentiality, patient-provider interaction)
12. What was the response of community/women on ART following the introduction of the new system? (Probe on the uptake of the system by women and whether they like the system or not and reasons behind each response, probe on issues on perceived stigma after using community organizations)
13. How do you assess/compare the new system of appointment and tracking compared to the old one? (Probe on the advantages and disadvantages of each, how well is it working, challenges in implementing it, suggestions to improve).
14. Do you think the new system of patient appointment and tracking should continue and be introduced to other facilities? (If yes why, if no why)
15. What else do you think could be done to improve the new system of patient appointment and tracking to increase adherence and retention to ART? (probe on potential interventions at both individual, health system and community levels)
16. Is there anything else you would like to share or add regarding patients’ appointment system and adherence/retention to care?

Thank you for participating in the study.

### 8.2.3 Patient exit interview guidelines

**TOOL 3: EXIT INTERVIEW GUIDE FOR WOMEN ON ART**

*For baseline in intervention clinics and endline in intervention and control clinics.*

(The interview will take place after signing the consent form, Respondents will answer questions in their own words and the interviewer will probe for elaboration.)

Name of health facility_______________District____________Ward________________-

Type of facility (circle): a) Public b) Private c) Faith Based

Level of facility (circle): a) Hospital, b) Health Center c) Dispensary

1. Can you please tell me a bit about yourself?
   1. (probe for religion, marital status, age, education level and occupation)
   2. When and where were you diagnosed with HIV? Was it at this clinic?
   3. When did you start using ART?
2. Do you know about CD4?
   1. If yes, can you tell me why this test is done?
   2. (probe on knowledge about CD4 changes and its implication)
3. Do you think it is important to take the medicines for your HIV regularly as recommended?
   1. (probe on successful treatment, drug resistance)
   2. How long do you think that you will need to keep taking these pills?
   3. How important is it to take all the pills each day?
4. Can you share with me your experience so far in taking ART?
   1. (probe on how easy/difficult it is taking ART tablets).
   2. (probe on motivating factors, including individual, health facility and community related)
   3. (probe on barriers, including individual, health facility and community related)
   4. What do you do to overcome the barriers?
   5. What do you think you can do to improve treatment adherence?
   6. Are there things that the clinic or community can do to support you in this? (probe for potential interventions at individual, health facility and community levels and which interventions are likely to be more accepted by the majority)
5. Have you ever missed a clinic appointment since you started taking ART?
   1. If no: Can you tell me how you have been so successful in attending the clinic on time?
   2. If yes: Can you share with me some of the reasons why you have missed clinic visits?
      1. Motivating factors for appointment keeping (individual, health facility and community related)
      2. Barriers (individual, health facility and community related)
      3. What do you do to overcome barriers?
6. Are there things that the MCH clinic or community can do to support you in keeping your appointments?
   1. (probe for potential interventions at individual, health facility and community levels; and which interventions are likely to be more accepted by the majority).
7. How are you satisfied with the ART services you receive at this MCH clinic? Probe on
   1. Confidentiality
   2. Patient-provider interaction/communication
   3. Availability of drugs and other equipment
   4. Waiting time/Timeliness of care
8. What recommendations do you have to improve the ART services you receive at this MCH clinic?
9. Are you assisted in managing your HIV by any community organization such as a home-based care organization, a community health worker or a local NGO?
   1. If yes how are you assisted? (Probe for every mentioned organization), How do you assess they their services? What can be done to improve such services?

***For baseline interview in intervention clinics, end the interview with question 10***

***For endline assessment in intervention facilities continue with questions 11-15.***

***For endline assessment in control facilities skip questions 10-15 and continue with question 16-18***

1. Is there anything else you would like to share about your experience on ART?

Thank you for participating in the study.

**Additional questions in intervention clinics at endline interview**

11. Are you aware of the new patient appointment and patient tracking system that has been introduced at this MCH clinic?

- 1. If yes, can you tell me about the new system? What is different from before? Do you like the new system? Has it made it easier or harder to attend the clinic on time? (Probe about issues of date and time negotiations, whether they like/dislike the new system and why)

12. Have you noticed any changes lately in what happens when you miss an appointment at this MCH clinic?

a) If yes, please explain the changes. Was there any follow up done before the new system?)

- 1. What is different from before? Do you like the new system?
  2. Has the new system made it easier or harder for you to attend the clinic on time? (Did you agree to be followed up by this i.e. person/organization/phone call, who has been following you up in case you miss your appointment?, are you comfortable to be followed up this way?)

13. Does following up at home make it easier or harder for you to attend the clinic on time?

a) What kinds of problems does following up at home create for you? (Probe on challenges related to perceived stigma from the follow up source)

14. What else do you think could be done to support you in attending the clinic on time and taking your medicines as recommended? (Probe on potential interventions at both individual, health system and community levels)

15. Is there anything else you would like to share or add regarding the new appointment system or other of your experience on HIV?

Thank you for participating in the study.

**Additional questions in control clinic at endline interview**

16. Have you heard of appointment and tracking system that has been introduced in some health facilities in this district? (If yes, probe on when did they hear of the intervention, what do they know about the purpose of intervention, what do they think are the advantages and disadvantages of the intervention)

17. For the past eight months, has there been any change in the way providers arrange your appointments and tracking missing visits? (probe for any intervention that took place in the past eight months, policy change related to appointment and tracking missing patients)

18. Is there anything else you would like to share or add regarding barriers to attendance and adherence to ART?

Thank you for participating in the study.

## 8.3 Quantitative Forms in Kiswahili

### 8.3.1 Facility Form (1 per facility)

| **Taarifa ya kujaza** | **Sehemu ya kadi ya CTC2** | **Aina ya taarifa ya kujaza** | **Ufupisho** | **Maelezo** |
| --- | --- | --- | --- | --- |
| Jina la kituo | Ipo katika sehemu ya juu ya kadi | Herufi |  | Chagua kutoka vituo vilivyoorodheshwa kulia |
| Namba ya utambulisho ya kituo |  | Nambari | Y | Tizama kutoka jedwali la kulia ( lilojazwa mojakwamoja) |
| Jina la Wilaya |  | Herufi |  | Tizama kutoka jedwali la kulia ( lilojazwa mojakwamoja) |
| Jina la mkuu wa kituo |  | Herufi |  | Jina la mkuu wa kituo |
| Jina la mtunza takwimu |  | Herufi |  | Jina la muhudumu anayesaidia ukusanyaji wa taarifa |
| Namba ya simu |  | Nambari |  | Namba ya simu ya muhusika katika kituo |
| Tarehe ya ukusanyaji taarifa za utafiti wa awali |  | Tar/mwez/mwk |  | Tarehe ya kufika kwa timu ya ukusanyaji taarifa za awali |
| Namba ya timu ya ukusanyaji taarifa za utafiti wa awali |  | Nambari |  | Namba ya timu |
| Tarehe ya ya mwanzo ya ukusanyaji taarifa za utafiti wa awali |  | Tar/mwez/mwk |  | Tarehe ya mwanzo ya ukusanyaji taarifa za utafiti za awali |
| Tarehe ya karibuni ya ukusanyaji wa taarifa za utafiti wa awali |  | Tar/mwez/mwk |  | Tarehe ya mwisho ya ukusanyaji wa taarifa za utafiti wa awali |
| Idadi ya wagonjwa wanaotumia ART walokuwepo katika tarehe ya utafiti wa awali |  | Nambari |  | namnba ya wagonjwa waliosalijiwa katika siku ya ukusanyaji wa taarifa |
| Idadi ya wagonjwa wanaotumia ART katika mwezi wa mwanzo |  | Nambari |  | Namba ya waginjwa waliosajiliwa katika mwezi ukusanyaji ukusanyaji mara ya kwanza wa taarifa za utafiti za awali |
| Tarehe ya ukusanyaji taarifa za ufuatiliaji |  | Tar/mwez/mwk |  | Tarehe ya timu kufika katika ukusanyaji taarifa katika ufuatiliaji |
| namba ya timu ya ukusanyaji taarifa za ufuatiliaji |  | Nambari |  | Namba ya timu |
| Tarehe ya awali ilorekodiwa katika ukusanyaji taarifa za |  | Tar/mwez/mwk |  | tarehe ya awali ya ukusanyaji taarifa za ufuatiliaji |
| Tarehe ya karibuni ilorikodiwa ya ukusanyaji wa tarifa za ufuatiliaji |  | Tar/mwez/mwk |  | Tarehe ya mwisho ya ukusanyaji wa taarifa za ufuatiliaji |
| Namba ya wagonjwa wanaotumia ART katika tarehe ya ukusanyaji taarifa za ufuatiliaji |  | Nambari |  | Namba ya wagonjwa waliopo katika siku ya ukusanyaji wa taarifa za ufuatiliaji |
| Idadi ya siku tangu ARV za mwazo zilipotolewa |  | Nambari |  | Idadi ya siku tangu ARV za mwazo zilipotolewa |
| Idadi ya siku tangu ARV zilipotolewa mara ya pili |  | Nambari |  | Idadi ya siku tangu ARV zilipotolewa mara ya pili (taarifa ya mtoa dawa ) |
| Idadi ya siku ARV ARV zilizotolewa katika ufuiatiliaji |  | Nambari |  | Idadi ya siku tangu ARV zilipotolewa wakati wa ufuatiliaji mwingine (taarifa ya mtoa dawa) |

### 8.3.2 Patient Form (1 per patient)

| **Taarifa za Kujaza** | **Sehemu ya kadi ya CTC2** | **Aina ya taarifa ya kujaza** | **Ufupisho** | **Maelezo** |
| --- | --- | --- | --- | --- |
| Namba ya utambulisho ya Kituo |  | Nambari | Y | Inajazwa kabla moja kwa moja toka kutoka katika taarifa za kituo |
| Namba ya utambulisho ya utafiti |  | Nambari | Y | Namba pekee ya mtiririko inayotolewa na kituo (huanza 101 kwa kila kituo) |
| Namba ya pekee ya utambulisho ya CTC | Ipo katika sehemu ya juu ya kadi | Nambari au herufi |  | Namba ya utambulisho inayotumika kwa kumbukumbu za kituo |
| Jina la mgonjwa | Ipo katika sehemu ya juu ya kadi | Herufi |  | Jina kama inavyoonekana katika kumbukumbu za kituo |
| Tarehe ya kuzaliwa | Ipo katika sehemu ya juu ya kadi | Tar/mwz/mwk |  | Kama inapatikana, vinginevyo acha wazi |
| Umri | Ipo katika sehemu ya juu ya kadi | Nambari |  | Kwa miaka kama inapatikana, vinginevyo acha wazi |
| Hali ya ndoa | Ipo katika sehemu ya juu ya kadi | Ufupisho |  | Angalia orodha ( chagua moja) |
| Rufaa kutoka | Ipo katika sehemu ya juu ya kadi | Ufupisho |  | Angalia orodha ( chagua moja) |
| Tarehe ya uthibitisho wa kuwa na VVU | Ipo katika sehemu ya juu ya kadi | Ufupisho |  | Kama inapatikana, vinginevyo acha wazi |
| Tarehe ya kusajiliwa kwenye huduma za VVU | Ipo katika sehemu ya juu ya kadi | Tar/mwz/mwk |  | Kama inapatikana, vinginevyo acha wazi |
| Tarehe ya kuthibitika kuanza dawa | Ipo katika sehemu ya juu ya kadi | Tar/mwz/mwk |  | Kama inapatikana, vinginevyo acha wazi |
| Tarehe ya kuthibitika na kuwa tayari kuanza dawa | Ipo katika sehemu ya juu ya kadi | Tar/mwz/mwk |  | Kama inapatikana, vinginevyo acha wazi |
| Tarehe ya kuanza dawa | Ipo katika sehemu ya juu ya kadi | Tar/mwz/mwk |  | Kama inapatikana, vinginevyo acha wazi |
| Umri wakati wa kuanza dawa | Ipo katika sehemu ya juu ya kadi | Nambari |  | Kama inapatikana, vinginevyo acha wazi |
| Ngazi/daraja la WHO | Ipo katika sehemu ya juu ya kadi | Nambari |  | Kama inapatikana, vinginevyo acha wazi |
| Kipimo cha CD4 kwa % wakati wa mwazo | Ipo katika sehemu ya juu ya kadi | Nambari |  | Kama inapatikana, vinginevyo acha wazi |
| Uzito wa mwili wakati wa mwanzo | Ipo katika sehemu ya juu ya kadi | Nambari |  | Kama inapatikana, vinginevyo acha wazi |

### 8.3.3 Visit Form (1 per visit)

## 8.4 Interview guidelines In Kiswahili

| **Taarifa za Kujaza** | **Sehemu ya kadi ya CTC2** | **Aina ya taarifa ya kujaza** | **Ufupisho** | **Maelezo ya ujazaji** |
| --- | --- | --- | --- | --- |
| Namba ya utambulisho pekee katika utafiti |  | Nambari | Y | Namba pekee ya utambulisho hutolewa kwa mpangilio (km. 101.001, 101.002, n.k.) |
| Utambuslisho wa hudhurio |  | Nambari | Y | Hutolewa moja kwa moja kwa mpangilio kufuatilia kwa upekee kwa kila mgonjwa |
| Tarehe ya hudhurio | 1 | Tar/Mwz/Mwk |  | Tarehe ya hudhurio la sasa |
| Aina ya hudhurio | 2 | Herufi |  | Tazama kifupisho 2 ( chagua mojawapo) |
| Uzito | 3 | Nambari |  | Uzito kwa kilo |
| Ngazi/ daraja la WHO | 5 | Nambari |  | Ngazi ya 1-4 |
| Kipimo cha CD4 | 6 | Nambari |  | Kipimo cha CD4 (%) |
| Dalili alizo nazo mgonjwa | 7 | Herufi |  | Tazama kifupisho cha 3 (chagua yote yanayohusika) |
| Sababu ya kupata ARV | 13 | Nambari |  | Tazama kifupisho 9 (chagua mojawapo) |
| Kundi la tiba ya ARV | 14 | Herufi |  | Tazama kifupisho 10 (chagua mojawapo na ingiza kifupisho tu) |
| Idadi ya siku alizopatiwa ARV | 14 | Nambari |  | Ingiza kama ipo (au ingiza idadi ya siku za kawaida ambazo dawa zinatolewa na kituo hiki |
| Uzingatiaji wa matumizi ARV | 15 | Herufi |  | Tazama kifupisho 11a (chagua mojawapo) |
| Uzingatiaji dhaifu wa matumizi ya ARV | 15 | Nambari |  | Tazama kifupisho 11b (chagua mojawapo iwapo tu kama iliyopita ilikuwa sawa na P) |
| Matibabu ya maginjwa nyemelezi | 16 | Nambari |  | Tazama kifupisho 12 (chagua mojawapo) |
| Tarehe ya hudhurio linalofuata | 23 | Tar/Mwz/Mwk |  | Tarehe ya siku ya hidhurio lijalo (lazima iwe tarehe ya mbele ya leo) |
| Hali ya mgonjwa katika ufuatiliaji | 24 | Herufi |  | Tazama kifupisho 16 (chagua mojawapo) |
| Kama uzingatiaji wa matumizi ya dawa umejadiliwa | upande wa nyuma | Herufi |  | N/H ( inayojitokeza ni H) |
| Maelezo ya uzingatiaji wa matumizi ya dawa | upande wa nyuma | Herufi |  | Ongeza maelezo (iwapo iliyopita ilikuwa ni N) |
| kujadili kuhusu miadi | upande wa nyuma | Herufi |  | N/H (inayojitokeza ni H) |
| Maelezo ya miadi | upande wa nyuma | Herufi |  | Ongeza maelezo (iwapo iliyopita ilikuwa ni H) |

### 8.4.1 District Health Officials interview guidelines in Kiswahili

**DODOSO NAMBA 1: MUONGOZO WA MAHOJIANO YA KINA KWA WATUMISHI WA AFYA NGAZI YA WILAYA (DAKTARI MKUU WA WILAYA, MRATIBU WA KITENGO CHA AFYA YA UZAZI NA MTOTO NA MRATIBU WA KITENGO CHA UKIMWI)**

(Kwa Wilaya zilizoshiriki katika utekelezaji wa kipindi cha awali na wilaya zitakazotembelewa mwishoni mwa utafiti ambazo hazitafanyiwa utekelezaji. Kwa wilaya zilizoshiriki katika utekelezaji na kutembelewa kipindi cha mwisho wa utafiti, tumia hojaji lililo mwisho kabisa)

(Mahojiano yataanza baada ya kusaini fomu ya ridhaa, wahojiwa watajibu maswali kwa maneno yao wenyewe na mhojaji atapeleleza kupata taarifa zaidi)

Wilaya____________Kata________________

1. Umefanya kazi katika wilaya hii kwa muda gani sasa?
2. Unafanya kazi katika cheo gani na umekuwa kwenye cheo hiki kwa muda gani sasa?
3. Ni lini kituo hiki kilianza kutoka huduma na tiba ya virusi vya UKIMWI katika kliniki za Afya ya uzazi na mwana? (Peleleza kujua ni vijiji/kata ngapi zinahudumiwa na kliniki husika)
4. Je wilaya na watoa huduma wa afya walipokea vipi mpango mzima wa kuingizwa kwa huduma na tiba ya Virusi Vya UKIMWI (VVU) katika kliniki za afya ya uzazi na mwana?
5. Je jamii ilipokea vipi mpango mzima wa kuingizwa kwa huduma na tiba ya VVU katika kliniki za afya ya uzazi na mwana?

**UZINGATIAJI NA, KUENDELEA KUWEPO KWENYE MATIBABU (Kwa ajili ya Kliniki za afya ya mzazi na mwana, Maswali kuhusu matumizi sahihi ya dawa yaulizwe tofauti na maswali ya uendelezaji wa tiba)**

1. Je unafikiri wanawake wenye VVU na wanaotumia dawa za kupunguza makali ya VVU katika kliniki za afya ya uzazi na mama na mwana wana matatizo katika uzingatiaji wa tiba? (peleleza kujua tatizo kwa ujumla)
2. Kwa nini unafikiri wanawake hawa wana matatizo katika uzingatiaji watiba kwenye wilaya yako?

Peleleza kujua

- 1. Sababu binafsi kama vile za kitabia, za ki miundo na kisaikolojia (tabia za kimaisha, umasikini, mawazo au huzuni)
  2. Sababu za mfumo wa afya kama vile Mahusiano kati ya mgonjwa na daktari, usiri, na ukosekanaji wa dawa
  3. Sababu za kijamii kama vile kutengwa, upatikanaji/ukosefu wa misaada ya kijamii

1. Je changamoto zilizopo katika kliniki za uzazi na mwana zinaweza kuchangia tatizo la uzingatiaji duni wa tiba na kutoendelea kuwepo katika tiba?
   1. Matatizo ya aina gani?
   2. Kwa nini unafikiri inakuwa hivi?
2. Je kuna mipango yoyote katika wilaya hii ya kuboresha uzingatiaji wa tiba za ARV? katika wilaya yako (Kama ndio tafadhali elezea mipango hii)
3. Je mipango ipi iliyopo katika kliniki za afya ya uzazi na mwana za wilaya hii ya kusaidia uzingatiaji wa tiba kwa wanaioshi na VVU? Peleleza kujua
   1. Mipango iliyopo katika kliniki na kwenye jamii na inafanya vipi kazi
   2. Ushirikishwaji wa watoa huduma wa nyumbani (HBC) na wanafanya vipi kazi kuboresha matumizi sahihi ya dawa
4. Je ni jinsi gani vituo vya afya ya uzazi na mwana vinatathimini uzingatiaji wa tiba kwa wanaoishi na VVU? (peleleza njia zinazotumia kama vile ripoti binafsi, kuhesabu dawa)
5. Je ni changamoto zipi vituo vya afya ya uzazi na mwana vinakutana nazo katika ufuatiliaji wa uzingatiaji wa tiba kwa watu wanaoishi na vVVU?
6. Je ni taarifa gani za uzingatiaji wa tiba zinaripotiwa wilayani? (peleleza kujua ni jinsi gani taarifa hizo zinatumika)
7. Je ni jinsi gani vituo vya afya ya uzazi na mwana vinatumia taarifa ya uzingatiaji wa tiba? (Peleleza kujua kama taarifa hizo zinatumika kwa ajili ya utekelezaji wowote)
8. Unafikiri nini kifanyike kuboresha uzingatiaji wa tiba na uwepo kwenye matibabu ya UKIMWI kwa wanawake katika vituo vya afya ya uzazi na mwana ? (peleleza kuhusu utekelezaji katika ngazi ya mtu binafsi, mfumo wa utoaji huduma za afya na katika ngazi ya jamii)
9. Je wilaya ina mipango yoyote ya kusaidia matumizi sahihi ya dawa na uwepo kwenye matibabu ya UKIMWI katika vituo vya afya ya uzazi na mwana? (peleleza kujua msaada wa wilaya kusaidia vituo vya afya na changamoto wanazokutana nazo)

**AHADI ZA WAGONJWA KUFIKA KWENYE MATIBABU NA UFUATILIAJI**

1. Je ni mfumo gani uliopo wa kuweka ratiba au apointmenti ya kuona wagonjwa na jinsi gani wanajua kama wamefika au hapana?

Peleleza kujua

a) Je wahudumu wanauliza kama siku iliyopendekezwa ni muafaka kwa mgonjwa?

b) Je wahudumu wanagundua vipi wagonjwa walioshindwa kufika siku waliyopangiwa?

1. Je ni jinsi gani wahudumu wanawafuatilia wale walioshindwa kufika siku waliopangiwa na changamoto zipi wanakutana nazo katika kuwafatilia? (Peleleza kujua kama wanawapigia simu, au hawawafatilii kabisa, na wilaya inahusika vipi katika hili)
2. Je uhusiano gani ulipo kati ya vituo vya kutolea huduma za afya na vikundi vya kijamii? (Je vituo vya afya vinafanya kazi na vikundi vya kijamii katika kufuatilia wale wanaoshindwa kufika katika matibabu –peleleza kujua matumizi ya vikundi vya kijamii au watoa huduma nyumbani kama vile vikundi au mashirika yanayojihusisha na shughuli za HIV na UKIMWI, wahudumu wa afya jamii). Kama hakuna uhusiano, je ni kwa nini?
3. Je unafikiri ni muhimu kujihusisha au kufanya kazi na vikundi au programu za kijamii kufatilia wanaoshindwa kufika kliniki siku walizopangiwa? Kama ndiyo kwa nini?
4. Je programu gani za kijamii ambazo unafikiri ni muhimu kushirikiana nazo katika ufuatiliaji wa wale wanaoshindwa kufika kliniki siku walizopangiwa) (kwa kila program peleleza kujua sababu husika ya ushirikishwaji wake)
5. Je unafikiri ni njia gani inaweza kukubalika zaidi ya kufatilia wale wanaoshindwa kufika kiniki siku walizongiwa?

***Malizia na swali no 23 iwapo ni utafiti wa mwanzo katika wilaya zitakazofaniyiwa utekelezaji***

1. Je kuna jambo ligine ungependa kuniambia au kunishirikisha kuhusu matumizi sahihi ya dawa na uwepo katika matibabu kwa wanaoishi na virusi vya UKIMWI katika wilaya hii?

***Kwa utafiti wa mwisho kwenye wilaya ambazo hazifanyiwia utekelezaji endelea na maswali 24-26***

1. Je umewahi kusikia kuhusu programu ya uwekaji miadi ya kufika kliniki na ufuatiliaji wa wanaoishi na VVU ambao umefanyika katika baadhi ya vituo vya kutolea huduma ya afya katika wilaya hii? (Kama ndio peleleza kujua walisikia lini kuhusu hiyo program, wanaelewa nini kuhusu jinsi program ilivyofanyika na wanafikiri ni nini faida na hasara ya programu hiyo)
2. Kwa miezi minane iliyopita, je kumekuwa na mabadiliko yoyote kwa jinsi ambavyo kliniki zimekuwa zikifanya miadi ya kuona wagonjwa na kufatilia wale wanaoshindwa kufika siku husika? (Peleleza kujua kama kuna program au utekelezaji wowote uliofanyika miezi minane iliyopita, na kama kuna sera yoyoye iliyobadilika kuhusiana na kuona na kufatilia wanaoshindwa kufika kliniki)
3. Je kuna jambo ligine ungependa kuniambia au kunishirikisha kuhusu matumizi sahihi ya dawa na uwepo katika matibabu kwa wanaoishi na VVU katika wilaya hii?

Asante kwa kushiriki katika utafiti

**MUONGOZO WA MASWALI KATIKA UTAFITI WA MWSHO KWA WILAYA ZITAKAZOFANYIWA UTEKELEZAJI**

1. Umefanya kazi katika wilaya hii kwa muda gani sasa?
2. Unafanya kazi katika cheo gani na umekuwa kwenye cheo hiki kwa muda gani sasa?
3. Je unafikiri wanawake wanaoishi na VVU katika kliniki za afya ya uzazi na mwana wana matatizo katikauzingatiaji wa tiba za ARV? (peleleza kujua tatizo kwa ujumla)
4. Je kwa nini unafikiri wanawake hawa wana matatizo katika uzingatiaji wa tiba kwenye wilaya yako?
   1. Sababu binafsi kama vile za kitabia, za ki miundo na kisaikolojia (tabia za kimaisha, umasikini, mawazo au huzuni)
   2. Sababu za mfumo wa afya kama vile Mahusiano kati ya mgonjwa na daktari, usiri, na ukosekanaji wa dawa
   3. Sababu za kijamii kama vile kutengwa, upatikanaji/ukosefu wa misaada ya kijamii
5. Je wilaya yako ina mipango yoyote ya kusaidia wanaoishi na VVU kupata tiba kwa usahihi zaidi?
6. Je una taarifa juu ya program au utekelezaji wa mpango unaohusu kuboresha miadi ya kufika kliniki na ufuatiliaji wa wale wanaoshindwa kufika kwa wakati uliotekelezwa katika kliniki za afya ya uzazi na mwana katika wilaya hii (Peleleza kujua ni lini programu ilianzishwa, na nani na kwa malengo gani)
7. Je mfumo mpya ya kufanya miadi na kufatilia wanaoshindwa kufika kliniki ina tofauti gani na ilivozoeleka kufanyika hapo mwanzo? (Peleleza kujua jinsi program mpya inavofanya kazi tofauti na walivyozoea zamani)
8. Kwa kutumia mfumo mpya, ni jinsi gani wahudumu wa afya wanafanya miaadi na wanaoishi na VVU juu ya siku ya kufika kliniki na kugundua kama kweli wamefika au la?

Peleleza kujua

- 1. Je wanauliza kama siku iliyopangwa ni muafaka kwa mgonjwa? (je wanajadili kuhusu tarehe na muda muafaka)
  2. Je ni wakati gani wa siku miaadi ya kufika kliniki hupangwa? (peleleza kujua kama ni asubuhi/mchana/jioni)
  3. Je wanagundua vipi wagonjwa ambao hawajafika siku walipangiwa?

1. Kwa kutumia mfumo mpya, ni mara ngapi wahudumu wanafatilia taarifa za mahudhurio na wanaoshindwa kufika siku husika? (Peleleza kujua ni mara ngapi taarifa za mahudhurio na wanaokosa kuhuduria zinangaliwa mara ngapi kama ni kila siku, baada ya wiki, mwezi na je wanaona ufuatiliaji huu ni sawa na changamoto gani wanakutana nzao)
2. Je ni jinsi gani wahudumu wa afya wanawafatilia wanawakae wanaoshindwa kufika kliniki siku walizopangiwa?(Peleleza kujua matumizi ya rasilimali jamii kama vile wadumu afya wa nyumbani, mashirika ya kijamii, matumizi ya simu, waishio na virusi vya UKIMWI wenzao) na waliamua vipi kutumia rasilimali jamii tajwa?
3. Je kuna mahusiano gani yaliyopo kati ya Kliniki za afya ya uzazi na mwana na mashirika au vikundi vya kijamii? (Wahudumu afya wa nyumbani, wahudumu afya jamii, vikundi vya kijamii vinavyojihusisha na UKIMWI na vikundi vingine) Je ni jinsi gani wanafanya kazi pamoja kufatilia wanawake wanaotumia dawa za kurefusha maisha? (Peleleza kujua majina ya mashirika au vikundi na kazi zake, faida ya ushirikiano na changamaoto zilizopo katika kufanya kazi pamoja)
4. Je unafikiri mfumo mpya wa kuona na kufuatilia wagonjwa umeboresha uzingatiaji wa tiba na uwepo katika tiba kwa wanawake wanaotumia dawa zaARV? (Kama ndio kwa nini? Peleleza kujua ni jinsi gani wanaona maboresho katika matumizi sahihi ya dawa sasa kulinganisha na zamani, je wanawake wengi zaidi wanafika siku waliyopangiwa kulinganisha na zamani? Kama ndio kwa nini?)
5. Je unafikiri huu mfumo mpya umechangia katika kuboresha utendaji wa vituo vya afya katika wilaya hii? (Peleleza kujua msongamano wa wagonjwa na muda wa kupata matibabu, upatikanaji wa dawa, usiri, mahusiano kati ya daktari na mgonjwa)
6. Je unaupenda vipi mfumo huu mpya wa miaadi na ufuatliaji wa wagonjwa kulinganisha na wa zamani? (Peleleza kujua faida na hasara za kila mfumo, ni jinsi gani mfumo unafanya kazi vizuri sasa, changamoto zake and nini kifanyike kutatua changamoto hizo)
7. Je wanajamii au wanawake wanaotumia dawa za kurefusha maisha waliupokea vipi mfumo huu mpya? (Peleleza kujua kama wanawake waliupenda/waliuchukia mfumo huu na kwani nini, peleleza kama kulikuwa na mambo ya kutengwa au kunyanyaswa na jamii baada ya mfumo kuanza kutumia vikundi au mashirika ya kijamii)
8. Je unafikiri mfumo mpya wa miadi kwa wagonjwa na ufuatiliaji uendelee na uanzishwe katika vituo vingine vya kutolea huduma ya afya (Kama ndio kwa nini na kama hapana kwa nini?)
9. Je kumekuwa na msaada wowote kutoka wilayani hadi katika vituo vya kutolea huduma za afya katika utekelezaji wa huu mfumo mpya (Kama ndio ni msaada gani na kama hakuna kwa nini?)
10. Ni nini unafikiri kifanyike kuboresha mfumo huu mpya ili kuongeza matumizi sahihi ya dawa na uwepo katika tiba kwa wanawake wanaoishi na VVU (Peleleza kujua nini kifanyike kwa ngazi za mtu binafsi, mfumo wa utoaji tiba and katika ngazi ya jamii)
11. Je kuna kitu kingine ungependa kunieleza au kunishirikisha kuhusu mfumo wa miaadi ya kuona wagojwa na uzingatiaji na kuendeleza kuwepo kwenye tiba kwa wanawake wanaotumia dawa za kurefusha maisha?

Asante kwa kushiriki katika utafiti

### 8.4.2 RCH Clinic Staff interview guidelines in Kiswahili

**DODOSO NAMBA 2: MWONGOZO WA MAHOJIANO YA KINA KWA WATOA HUDUMA WA AFYA (KITENGO CHA AFYA YA UZAZI NA MTOTO)**

(Kwa watoa huduma wa vituo vya kutolea huduma za afya vitakazofanyiwa utekelezaji kipindi cha awali na na wale wa vituo vitakavyotembelewa mwishoni mwa utafiti ambavyo havitafanyiwa utekelezaji. Kwa wale wa vituo vitazofanyowa utekelezaji na kutembelewa kipindi cha mwisho wa utafiti, tumia hojaji lililo mwisho kabisa)

(Mahojiano yataanza baada ya kusaini fomu ya ridhaa, wahojiwa watajibu maswali kwa maneno yao wenyewe na mhojaji atapeleleza kupata taarifa Zaidi)

Jina la kituo_______________Wilaya____________Kata________________-

Aina ya kituo (zungushia) a) Umma b) Binafsi c) Dini

Ngazi ya kituo (zungushia): a) Hospitali b) Kituo cha afya c) Zahanati

1. Umefanya kazi kwa muda gani sasa (peleleza kujua a) katika kutoa huduma za afya kwa ujumla b) katika kituo hiki and c) Katika kutoa huduma za HIV na UKIMWI? d) katika kutoa huduma za HIV na UKIMWI katika kituo hiki?.
2. Kituo hiki kilianza lini kutoka huduma na tiba ya VVU katika kliniki za Afya ya uzazi na mtoto? (peleleza kujua ni vijiji/kata ngapi zinahudumiwa na kliniki husika)
3. Je watoa huduma wa afya walipokea vipi mpango mzima wa kuingizwa kwa huduma na tiba ya VVU katika kliniki za afya ya uzazi na mwana?
4. Je jamii ilipokea vipi mpango mzima wa kuingizwa kwa huduma na tiba ya vVVU katika kliniki za afya ya uzazi na mwana?

**UZINGATIAJI NA UENDELEVU WA KUWEPO KWENYE TIBA (Kwa ajili ya Kliniki za afya ya mzazi na mtoto, Maswali kuhusu uzingatiaji wa tiba yaulizwe tofauti na maswali ya uendelevu wa kuwepo kwenye tiba)**

1. Unaelewa nini kuhusu uzingatiaji wa tiba za kurefusha maisha? (peleleza kujua tofauti kati ya uzingatiaji wa tiba na uendelevu wa kuwepo kwenye tiba)
2. Je mnafanya tathimini ya uzingatiaji wa tiba kwa wanaoishi na VVU katika kituo hiki? (kama ndiyo, peleleza njia zinazotumia kama vile ripoti binafsi, kuhesabu dawa)
3. Je ni changamoto zipi vituo vya afya ya uzazi na mtoto vinakutana nazo katika ufuatiliaji wa matumizi sahihi ya dawa kwa watu wanaoishi na VVU? (Je vituo vinatumia vipi taarifa hizi, peleleza kujua kama zinatumika kwa ajili ya utekelezaji wowote)
4. Je kituo kina changamoto gani katika kusaidia wanawake wanaotumia dawa za kupunguza makali ya VVU kuzingatia tiba na kuendelea kuwepo kwenye tiba?
5. Je unafikiri wanawake wanaotumia dawa za kkupunguza makali ya VVU wana matatizo yapi katika matumizi sahihi ya dawa katika kituo hiki? peleleza kujua
   1. Sababu binafsi kama vile za kitabia, za ki miundo na kisaikolojia (tabia za kimaisha, umasikini, mawazo au huzuni)
   2. Sababu za mfumo wa utoa huduma za afya kama vile Mahusiano kati ya mgonjwa na watoa huduma ya afya, usiri, na ukosekanaji wa dawa
   3. Sababu za kijamii kama vile unyanyapaa, upatikanaji/ukosefu wa misaada ya kijamii
6. Je Kuna changamoto katika kliniki za uzazi na mtoto zinazochangia tatizo la uzingatiaji wa tiba na uendelevu duni wa kuwepo katika tiba? (Je ni changamoto zipi?kwa nini unafikiri inakuwa hivi?)
7. Je kuna mipango/mikakati yoyote katika kituo hiki ya kuboresha uzingatiaji wa tiba ya ARV na uendelevu wa uwepo katika tiba? (kama ndio tafadhali elezea ni mipango gani? imeanza lini? Je ni kwa nini mmeitekeleza )
8. Unafikiri nini kifanyike kuboresha uzingatiaji wa tiba na uendelevu wa uwepo kwenye tiba ya UKIMWI kwa wanawake katika vituo vya afya ya uzazi na mtoto ? (peleleza kuhusu utekelezaji katika ngazi ya mtu binafsi, mfumo wa utoaji huduma za afya na katika ngazi ya jamii)

**MFUMO WA KUBORESHA MIADI YA KUFIKA KWENYE MATIBABU NA UFUATILIAJI WA WAGONJWA**

1. Je ni mfumo upi unatumika kuweka miadi au apointmenti kwa wakinamama katika kurudi kliniki kwa ajili ya huduma za tiba za ARV?
2. Je kuna njia yoyote ya kujua kama wamefika kliniki au la?

(Omba kuonan regista and uliza ni jinsi gani inatumika)

Peleleza kujua

1. Je watoa huduma wanauliza kama siku iliyopendekezwa ni muafaka kwa mgonjwa?
2. Je watoa hudumawanaweza kuwagundua vipi wagonjwa walioshindwa kliniki kufika siku waliyopangiwa? (Peleleza kujua ni lini wanagundua)
3. Je kuna mfumo wa kuwafuatilia nyumbani wale wanaoshindwa kufika siku waliyopangiwa?
   1. Kama ndio je ni changamoto zipi wanakutana nazo (peleleza kujua kama wanawapigia simu au hakuna ufuatiliaji wowote unaofanyika?)
4. Je mnashirikiana na mipango gani ya kijamii kufatilia wanaotumia dawa za kupunguza makali? (Kama vile vikundi au mashirika ya kijamii) Peleleza kujua
   1. (matumizi ya vikundi vya kijamii au watoa huduma nyumbani kama vile vikundi au mashirika yanayojihusisha na shughuli za HIV na UKIMWI, wahudumu wa afya jamii) Kama hakuna uhusiano, je ni kwa nini?
5. Je unafikiri ni muhimu kushirikiana au kufanya kazi na vikundi au mipango ya kijamii kufuatilia wanaoshindwa kufika kliniki siku walizopangiwa?
   1. (Kwa nini? faida na hasara zake)
   2. Je unafikiri ni njia gani inaweza kukubalika zaidi kufatilia wale wanaoshindwa kufika kiniki siku walizongiwa?

***Malizia na swali na. 18 iwapo ni utafiti wa mwanzo katika vituo vitakavyofaniyiwa utekelezaji, endelea na swali 19-21 kwa utafiti wa mwisho katika vituo ambavyo havitafanyiwa utekelezaji)***

1. Je kuna jambo ligine ungependa kuniambia au kunishirikisha kuhusu uzingatiaji wa tiba na uendelevu wa uwepo katika tiba ya wanaoishi na VVU katika kituo hiki? (uliza pia kama ana chochote cha kuongeza kuhusu uwekaji mihadi ya kuona wagonjwa)

Asante kwa kushiriki katika utafiti

1. Je umewahi kusikia kuhusu mpango wa uwekaji mihadi ya kufika kliniki na ufuatiliaji wa wanaoishi na VVU ambao umefanyika katika baadhi ya vituo vya kutolea huduma za afya katika wilaya hii? (Kama ndio peleleza kujua walisikia lini kuhusu mpango huo, wanaelewa nini kuhusu jinsi program ilivyofanyika na wanafikiri ni nini faida na hasara ya programu hiyo)
2. Kwa miezi minane iliyopita, je kumekuwa na mabadiliko yoyote kwa jinsi ambavyo kliniki zimekuwa zikifanya mihadi ya kuona wagonjwa na kufatilia wale wanaoshindwa kufika siku ya miadi? (Peleleza kujua kama kuna mpango au utekelezaji wowote uliofanyika miezi minane iliyopita, na kama kuna sera yoyoye iliyobadilika kuhusiana na kuona na kufatilia wanaoshindwa kufika kliniki)
3. Je kuna jambo ligine ungependa kuniambia au kunishirikisha kuhusu uzingatiaji na uendelevu wa uwepo katika tiba kwa wanaoishi na VVU katika kituo hiki? (uliza pia kama ana chochote cha kuongeza kuhusu uwekaji mihadi ya kuona wagonjwa)

Asante kwa kushiriki katika utafiti

**MWONGOZO WA MASWALI KATIKA UTAFITI WA MWSHO KWA VITUO VITAKAVYOFANYIWA UTEKELEZAJI**

1. Jina la kituo_______________Wilaya____________Kata________________
2. Aina ya kituo (zungushia) a) Serikali b) Mtu binafsi c) Dini
3. Ngazi ya kituo (zungushia): a) Hospitali b) Kituo cha afya c) Zahanati
4. Umefanya kazi kwa muda gani sasa (peleleza kujua a) katika kutoa huduma za afya kwa ujumla b) katika kituo hiki and c) Katika kutoa huduma za maswala ya HIV na UKIMWI? d) katika kutoa huduma za HIV na UKIMWI katika kituo hiki?
5. Je kituo kina changamoto gani katika kusaidia wanawake wanaotumia dawa za kupunguza makali ya VVU, uzingatiaji wa tiba na uendelevu wa kuwepo kwenyetiba?
6. Je unafikiri wanawake wanaotumia dawa za kurefusha maisha wana matatizo yapi katika matumizi sahihi ya dawa katika kituo hiki? peleleza kujua
   1. Sababu binafsi kama vile za kitabia, za ki miundo na kisaikolojia (tabia za kimaisha, umasikini, mawazo au huzuni)
   2. Sababu za mfumo wa utoa huduma za afya kama vile Mahusiano kati ya mgonjwa na mtoa huduma ya afya, usiri, na ukosekanaji wa dawa
   3. Sababu za kijamii kama vile unyanyapaa, upatikanaji/ukosefu wa misaada ya kijamii
7. Je kituo chako kina mipango yoyote ya kusaidia wanaoishi na VVU kumeza dawa zao kwa kwa mfululizo?
8. Je una taarifa juu ya mpango au utekelezaji wa mpango unaohusu miadi ya kufika kliniki na ufuatiliaji wa wale wanaoshindwa kufika siku waliopangiwa uliotekelezwa katika kliniki hii? (Peleleza kujua ni lini programu ilianzishwa, na nani na kwa malengo gani)
9. Je mfumo mpya ya kufanya miaadi na kufuatilia wanaoshindwa kufika kliniki una tofauti gani na ilivozoeleka kufanyika mwanzo? (peleleza kujua jinsi program mpya inavofanya kazi tofauti na walivyozoea zamani).
10. Kwa kutumia mfumo mpya, ni jinsi gani wahudumu wa afya wanafanya miaadi na wanaoishi na VVU juu ya siku ya kufika kliniki na kugundua kama kweli wamefika?

(Omba kuona rejesta na uliza inatumika vipi?) peleleza kujua

- 1. Je wanauliza kama siku iliyopangwa ni muafaka kwa mgonjwa? (je wanajadili kuhusu tarehe na muda muafaka)
  2. Je ni wakati gani wa siku mihadi ya kufika kliniki hupangwa? (peleleza kujua kama ni asubuhi/mchana/jioni)
  3. Je wanagundua vipi wagonjwa ambao hawajafika siku waliyopangiwa?

1. Kwa kutumia mfumo mpya, ni mara ngapi wahudumu wanafuatilia taarifa za mahudhurio na wanaoshindwa kufika siku husika? (Peleleza kujua ni mara ngapi taarifa za mahudhurio na wanaokosa kuhuduria zinangaliwa kama ni kila siku, baada ya wiki, mwezi na je wanaona ufuatiliaji huu ni sawa na changamoto gani wanakutana nzao)
2. Je ni jinsi gani wahudumu wa afya wanawafatilia wanawake wanaoshindwa kufika kliniki siku walizopangiwa?(Peleleza kujua matumizi ya rasilimali jamii kama vile wadumu afya wa nyumbani, mashirika ya kijamii, matumizi ya simu, waishio na virusi vya UKIMWI wenzao) na waliamua vipi kutumia rasilimali jamii tajwa?
3. Ni sababu zipi zaidi hutajwa na wakinamama wanaoshindwa kufika siku ya miaadi yao kwa siku moja hadi tatu?
4. Je ni mashirika gani ya kijamii au vikundi mnavyofanya navyo kazi kufatilia wanawake wanaotumia dawa za kurefusha maisha (peleleza kama wanatumia Wahudumu afya wa nyumbani, wahudumu afya wa jamii au mashirika mengine ya jamii- Peleleza kujua majina ya vikundi/mashirika tajwa, kazi zake, faida na hasara za kufanya kazi pamoja)
5. Je mnafanya tathimini ya uzingatiaji wa tiba kwa wanaoishi na VVU? (kama ndiyo, peleleza njia zinazotumika kwa kutumia mfumo mpya, faida na hasara zake, nini kinafanyika inwapo kunakuwa na uzingatiaji duni - kama vile kufanya mikutano ya mwezi kujadili suala hilo)
6. Je unafikiri mfumo mpya wa kuona na kufatilia wagonjwa umeboresha uzingatiaji wa tiba na uendelevu wa uwepo katika tiba kwa wanawake wanaotumia dawa za kurefusha maisha? (Kama ndio kwa nini? Peleleza kujua ni jinsi gani wanaona maboresho katika uzingatiaji wa tiba kwa sasa kulinganisha na zamani, je wanawake wengi zaidi wanafika siku waliyopangiwa kulinganisha na zamani? Kama ndio kwa nini?)
7. Je unafikiri mfumo huu mpya umepunguza matatizo au changamoto katika kituo hiki? (Kama vile msongamano wa wagonjwa na muda wa kupata matibabu, upatikanaji wa dawa, usiri wa utoaji matibabu, mahusiano kati ya daktari na mgonjwa)
8. Je wanajamii au wanawake wanaotumia dawa za kupunguza makali ya VVU waliupokea vipi mfumo huu mpya? (peleleza kujua kama wanawake waliupenda/waliuchukia mfumo huu na kwani nini, peleleza kama kulikuwa na mambo ya kutengwa au kunyanyaswa na jamii baada ya mfumo kuanza kutumia vikundi au mashirika ya kijamii)
9. Je unalinganisha vipi mfumo huu mpya na wa zamani (Peleleza kujua hasara na faida za kila mfumo? Unafanyaje kazi, changamoto katika utekelezaji na nini cha kuboresha)
10. Je unafikiri mfumo mpya wa kuona wagonjwa na kuwafatilia uendelee na uanzishwe katika vituo vingine vya kutolea huduma za afya (Kama ndio kwa nini na kama hapana kwa nini?)
11. Ni nini unafikiri kifanyike kuboresha mfumo huu mpya wa kuona wagonjwa na kuwafatilia ili kuongeza uzingatiaji wa tiba na uendelevu wa uwepo katika tiba kwa wanawake wanaoishi na VVU (Peleleza kujua nini kifanyike kwa ngazi za mtu binafsi, mfumo wa utoaji tiba and katika ngazi ya jamii)
12. Je kuna kitu kingine ungependa kunieleza au kunishirikisha kuhusu mfumo wa mihadi ya kuona wagojwa na uwepo kwenye tiba kwa wanawake wanaotumia dawa za kupunguza makali ya VVU?

Asante kwa kushiriki katika utafiti

### 8.4.3 Patient exit interview guidelines in Kiswahili

**DODOSO NAMBA 3: MWONGOZO WA MAHOJIANO NA WANAWAKE WANAOTUMIA DAWA ZA KUPUNGUZA MAKALI YA VIRUSI VYA UKIMWI**

***Kwa utafiti wa mwanzo kwenye vituo vitakavyofanyiwa utekelezaji na utafiti wa mwisho katika vituo ambavyo havitafanyiwa utafiti***

(Mahojiano yataanza baada ya kusaini fomu ya ridhaa, wahojiwa watajibu maswali kwa maneno yao wenyewe na mhojaji atapeleleza kupata taarifa zaidi)

Jina la kituo cha kutolea huduma ya afya_______________Wilaya____________Kata___________

Aina ya kituo (zungushia): a) Umma b) Binafsi c) Dini

Ngazi ya kituo (zungushia): a) Hospitali b) Kituo cha afya c) Zahanati

1. Je unaweza kunieleza kidogo kuhusu historia yako?
   1. (peleleza kuhusu dini, hali ya ndoa, umri, kiwango cha elimu, na shuguli yako)
   2. Ni lini na wapi ulipima na kugundulika una virusi vya UKIMWI? Je ilikuwa ni katika kituo hiki?
   3. Ni lini ulianza kutumia dawa za kurefusha maisha?
2. Je unaelewa nini kuhusu kipimo cha CD4?
   1. Je unaweza kuniambia kwa nini kipimo hiki kinafanyika?
   2. (peleleza kujua uelewa kuhusu mabadiliko ya kiwango cha chembechembe za CD4 na madhara yake?)
3. Je unafikiri ni muhimu kumeza dawa za kupunguza makali ya Virusi vya Ukimwi (VVU) kwa usahihi kama inavyoshauriwa?
   1. (peleleza kuhusu mafanikio ya matibabu, usugu wa dawa)
   2. Je unafikiri unatakiwa kumeza dawa hizi kwa muda gani?
   3. Je kuna umuhimu gani kunywa dawa kila siku?
4. Je unaweza kuniambia uzoefu wako katika kutumia dawa za kupunguza makali ya VVU?
   1. (peleleza kujua ni rahisi au vigumu kiasi kumeza dawa).
   2. (peleleza kujua sababu zinazomshawishi kumeza dawa –sababu za kibinafsi, zinazoendana na mfumo wa utoaji huduma, na za jamii kwa ujumla)
   3. (Peleleza changamoto za kumeza dawa - za kibinafsi, zinazoendana na mfumo wa utoaji huduma, na za jamii kwa ujumla)
   4. Je unapambana vipi na changamoto hizo?
   5. Je unafikiri unaweza kufanya nini kusaidia kutumia dawa zako kwa usahihi?
   6. Je kuna vitu ambavyo klinki au jamii ingeweza kufanya kukusaidia kutumia dawa zako kwa usahihi (peleleza kujua kitu ambacho kinaweza kufanyika katika ngazi ya mtu binafsi, mfumo wa utoa huduma na jamii kwa ujumla na amabacho kitakubalika zaidi na jamii)
5. Tokea umeanza kutumia dawa za kurefusha maisha umeshawahi kukosa kliniki siku yako ya kuchukua dawa?
   1. Kama hapana: Unaweza kunieleza ni jinsi gani umejitahidi sana kuhudhuria kliniki siku yako ya miadi?
   2. Kama ndio: Unaweza kunieleza sababu za kushindwa kufika siku husika kliniki?
      1. Sababu zilizokushawishi kuhuduria siku uliyopangiwa? (sababu binafsi, za mfumo wa utoaji huduma na sababu za kijamii)
      2. Vizuizi (binafsi, za mfumo wa utoaji huduma na sababu za kijamii)
      3. Je unafanya nini kupambana na changamoto hizo?
6. Je kuna vitu ambavyo kliniki au jamii kwa ujumla ingeweza kufanya kukusaidia kutokukosa kuhudhuria kliniki siku uliyopangiwa?
   1. (peleleza kujua kitu ambacho kinaweza kufanyika katika ngazi ya mtu binafsi, mfumo wa utoa huduma na jamii kwa ujumla na amabacho kitakubalika Zaidi na jamii)
7. Je unaridhika vipi na huduma za dawa unazopata hapa kliniki? peleleza kujua
   1. Usiri
   2. Mahusiano/mawasiliano kati ya mgonjwa na watoa huduma
   3. Upatikananji wa dawa na vifaa vingine
   4. Muda wa kusubiria huduma/Upatikanaji wa huduma kwa uharaka
8. Unapendekeza nini kifanyika kuboresha huduma za dawa za kupunguza makali ya VVU katika klinki hii?
9. Je unasaidiwa kwa lolote katika kukabiliana na hali yako na mashirika au vikundi vya kijamii kama vile wahudumu afya nyumbani , wahudumu afya jamii and mashirika binafsi?
   1. Kama ndio je unasaidiwa kivipi? (Pelelza kujua kwa kila chanzo kilichotajwa, unatathimini vipi huduma zao, na nini unafikiri kifanyike kuboresha huduma hizo?

***Kwa utafiti wa mwanzo (baseline) kwenye vituo vitakavyofanyiwa utekelezaji wa utafiti, malizia na swali namba 10. Kwa utafiti wa mwisho (endline) katika vituo vitakavyofanyiwa utekelezaji wa utafiti endelea (intervention facilities0 na swali 11-15. Kw autafiti wa mwisho katika vituo ambavyo havitafanyiwa utekelezaji wa utafiti (control facilities) usiulize maswali 10-15 ila endelea na maswali 16-18***

1. Kuna kitu kingine ungependa kunieleza au kuongeza kuhusu uzoefu wako katika kutumia dawa za kurefusha maisha?

Asante kwa kushiriki katika utafiti.

**Maswali ya nyongeza katika vituo vitakavyofanyiwa utekelezaji na kutembelewa kwa utafiti wa mwisho**

11. Je umewahi kusikia kuhusu mpango wa kuboresha uwekaji miaadi ya kufika kliniki na ufuatiliaji wa wanaoishi na VVU ambao umefanyika katika kliniki za afya ya mama na mtoto?

- 1. Kama ndio unaweza kunielezea kuhusu programu hii ? Nini kimefanyika tofauti na mwanzo. Je umeupenda mpango huu mpya? Je imekusaida au kutokukusaida kufika kliniki kama inavyotakiwa (Peleleza kujua tarehe za kufika klinkini na majadiliano kuhusu muda wa kufika kliniki , na kama wanaipenda au hawaipendi program hii mpya na kwa nini?)

12. Je umegundua chochote hivi karibuni ni nini kinafanyika pale unaposhidwa kuhudhuria klinki hii kama unavyotakiwa?

a) kama ndio tafadhali elezea mabadiliko haya. Je ulikuwa unafatiliwa hapo kabla?

- 1. Ni nini kinafanyika tofauti na mwanzo? Je unapenda mpango au mfumo huu mpya?
  2. Je mpango au mfumo huu mpya umekurahisishia au kutokukurahishia kufika kliniki siku ya miaadi? (Je ulikubaliwa kufuatiliwa kwa njia hii? na mtu huyu/shirika/kwa njia ya simu, nani amekuwa akikufatilia ukishindwa kufika klinki? Je unaridhika kufatiliwa kwa njia hii?

13. je kukufatilia nyumbani inakurahisishia au inakupa ugumu kuhudhuria kliniki kwa wakati unaotakiwa?

a) Ni matatizo gani yanayosababishwa na wewe kufuatiliwa nyumbani? (Peleleza kujua changamoto kama kutengwa na kunyanyaswa na yule au wale wanaokufatlilia kwa ajili ya matibabu)

14. Unafikiri nini kifanyike kukusaidia kuhudhuria kliniki kwa wakati na kutumia dawa zako kama inavyoshauriwa (peleleza kujua kitu ambacho kinaweza kufanyika katika ngazi ya mtu binafsi, mfumo wa utoa huduma na jamii kwa ujumla)

15. Kuna kitu kingine ungependa kunieleza au kuongeza kuhusu uwekaji miaadi ya kurudi kliniki or chochote kuhusu uzoefu wako kama mtu anayeishi na virusi vya UKIMWI?

Asante kwa kushiriki katika utafiti.

**Maswali ya nyongeza kwa utafiti wa mwisho kwa vituo ambavyo havitafanyiwa utekelezaji**

16. Je umewahi kusikia kuhusu mpango wa uwekaji miaadi ya kufika kliniki na ufuatiliaji wa wanaoishi na VVU ambao umefanyika katika kliniki za afya ya uzazi ya mama na mtoto katika wilaya hii? (Kama ndio unaweza kunielezea ni lini ulisikia kuhusu programu hii? Je unaelewa nini dhumuni la programu hii, unafikiri nini ni faida na hasara za programu hii)

17. Kwa miezi nane iliyopita je kumekuwa na tofauti kwa jinsi wahudumu wanavyopanga miaadi yako ya kufika kliniki na ufatiliaji pale unaposhindwa kufika kliniki siku husika? Peleleza kujua kama kuna program au utekelezaji wowote uliofanyika miezi minane iliyopita, na kama kuna sera yoyoye iliyobadilika kuhusiana na kupanga mihadi na kufatilia wanaoshindwa kufika kliniki

18. Je kuna kingine ungependa kunieleza ua kuongeza kuhusu changamoto za kuhudhuria kliniki na kuendelea kuwepo katika matibabu?

Asante kwa kushiriki katika utafiti.

# 9.0 INFORMATION AND INFORMED CONSENT FORMS

##
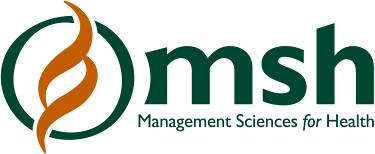
9.1 Information in English

**INFORMATION SHEET**

**Improving Adherence to ART at Reproductive and Child Health Clinics integrating Option B+ in Tanzania**

**About the Study**

Until recently, all antiretroviral therapy (ART) for HIV/AIDS in Tanzania was administered through specialized clinics. In the middle of last year, Tanzania officially adopted Option B Plus for pregnant women in all their Mother and Child Health (MCH) clinics, whereby all HIV positive women now receive lifelong ART regardless of their CD4 count. However, the Ministry of Health and Social Welfare (MOHSW) reports that adherence and retention to treatment are a problem under the program. Good adherence to antiretroviral treatment is essential if treatment is to be successful and if developing resistance to the medicines is to be avoided. Our research aims at among other things identifying factors related to poor adherence and retention in order to come up with an intervention to improve adherence and retention among ART clients, facilitate the management of staff workload and promote sustainable and consistent clinic attendance by HIV-positive patients.

**Study Methods**

As part of this study we will be collecting data at health facilities, conducting interviews with district health officials, clinicians and women on ART in Mbeya region

**Research Regulations**

Your participation in this research project is voluntary: this means that you do NOT have to participate if you do not want to. If you agree to participate, you can refuse to answer any questions that you do not feel comfortable answering. You can also stop the interview at any time, and this will not affect your right to care.

Recording devices will be used to record your responses. The information you provide is confidential: this means we will not tell anyone what you said in this interview. Only a code will be used to identify your answers without identifying you. This information will only be used for research. The institutions responsible for monitoring this study may inspect these records to make sure your rights are protected, but they will not be able to link your answers with your full name.

There is no direct benefit to participation in this study; however, having a better understanding of factors related to poor adherence and retention is essential to come up with relevant interventions to improve the problem and benefit client on ART and the community at large. There is no risk that can be involved in your participation in this study. You can ask a researcher any question during the interview/study.

If you have any further questions about this study or about your rights as a study subject, you can contact Dr. Suleiman Kimatta at cell Number 0684606108 Should you have questions about your rights as a research participant, please contact the National Institute for Medical Research, PO Box 9653, Dar es Salaam, Tanzania, telephone +255-22-2121400.

##
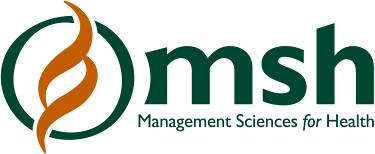
9.2 Informed consent form in English

**Consent Form for study participants**

**Study Title:** **Improving Adherence to ART at Reproductive and Child Health Clinics integrating Option B+ in Tanzania**

The interview will take up to 1 hour to complete, and will be administered by ______________.

During the interview, you will be asked questions about you related experience as a district health official, or health care provider or as a woman on ART.

Signing ( or marking an X ) on this form indicates that you have been told the nature of the study and your involvement in it.

I ____________________________ have read the information sheet concerning this study [or have discussed this information verbally] and I have been told what will be required of me and what will happen to me if I take part in this study. Specifically, I have read the General Information Sheet describing the study, the sponsoring institutions, and the terms of my participation, and I have been given a copy of this information sheet for my reference.

I have been told that the interview will take approximately one hour to complete.

My questions concerning this study have been answered.

I have been told that at any time I may withdraw from this study without giving a reason.

I have been told that some of what I say may be quoted in reports of the findings of this research, but this will be in a way that does not identify me.

I hereby consent to participate in the study.

Interviewee’s signature (or X) ___________________________ Date: __________________

Interviewee’s printed name ___________________________

Interviewer’s signature _______________________________ Date: __________________

Interviewer’s printed name ___________________________

Name of the witness ________________________________ Date: __________________

Signature of the witness __________________________________

Ethical review granted by the National Health Research Ethical Review Committee

National Institute for Medical Research; 2448 Ocean Road, P.O.Box 9653

Dar es Salaam-Tanzania

Dr. Suleiman Kimatta, Local Study PI

## 9.3 Information in Kiswahili


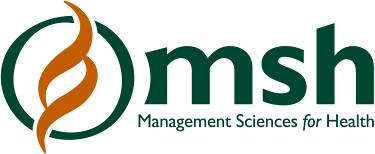


**Karatasi ya Taarifa Kuhusu Utafiti**

**Kuboresha uzingatiaji na uendevevu wa kuwepo katika tiba za ARV katika kliniki za afya ya uzazi wa Mama na Mtoto zinazotoa huduma ya “Option B Plus” Tanzania**

**Kuhusu utafiti**

Mpaka hivi karibuni, dawa za kurefusha maisha kwa ajili ya wanaoishi na virusi vya UKIMWI zilikuwa zinatolewa katika kliniki maalumu nchini Tanzania. Katikati ya mwaka uliopita, Tanzania ilianzisha mfumo mpya ujulikanao kama ‘option B plus’ katika kliniki za mama na mtoto ambapo wanawake wote wanaoishi na virusi vya UKIMWI wanapata huduma ya dawa za kurefusha maisha (ARV) bila kujali kiwango cha CD4. Pamoja na hayo, Wizara ya Afya na Ustawi wa Jamii, inaripoti kuwa uzingatiaji na uendelevu wa tiba imekuwa ni changamoto katika mpango huu. Uzingatiaji wa tiba za kurefusha maisha na uendelevu katika tiba ni muhimu kwa tiba kamilifu na kuzuia usugu wa dawa. Utafiti huu, pamoja na mambo mengine una lengo la kutafiti changamoto zinazohusiana na uzingatiaji hafifu na uendelevu duni katika tiba ili kuja na mbinu mahususi ya kuboresha uzingatiaji na uendelevu katika tiba, kurahisisha ufanyaji kazi wa wahudumu wa afya na kusaidia kuboresha mahudhurio endelevu ya tiba kwa wanaoishi na virusi vya UKIMWI.

**Mbinu za Utafiti**

Kama sehemu ya utafiti huu tutakusanya taarifa husika katika vituo vya afya, tutafanya mahojiano ya kina na wafanyakazi wa afya wa wilaya, wahudmu wa vituo vya kutolea huduma za afya, wanawake wenye VVU na wanaotumia dawa za kurefusha maisha katika mkoa wa Mbeya.

**Taratibu za Utafiti**

Ushiriki wako kwenye utafiti huu ni wa hiari. Hii inamaanisha kuwa hutakiwi kushiriki ikiwa hutaki kufanya hivyo. Waweza kukataa kujibu swali lolote ambalo hujisikii kulijibu na waweza kusitisha mahojiano wakati wowote. Mahojiano yetu yatarekodiwa. Taarifa utakazotoa ni siri inamaanisha kuwa hatutamwambia mtu yeyote kuhusu kile ulichokisema kwenye mahojiano. Namba ya siri tu itatumika kuyatambulisha majibu yako pasipo kukutambulisha wewe. Taarifa hizi zitatumika kwa ajili ya utafiti tu. Taasisi husika zinazoshughulikia utafiti huu zinaweza kukagua taarifa hizi huku wakihakikisha kuwa haki zako zinahifadhiwa. Jina lako halitatokea popote na halitahusishwa na kile ulichokizungumza.

Hakuna faida ya moja kwa moja utakayopata kwa kushiriki kwenye utafiti huu, hata hivyo kuelewa kuhusu changamoto wanazokutana nazo watu wanaoishi na VVU katika uzingatiaji na uendelevu duni katika tiba ni muhimu kwa ajili ya kubuni mbinu za kukabiliana na changamoto hizo, mbinu amabazo zinaweza kuwanufaisha wanaotumia dawa za kurefusa maisha na jamii kwa ujumla. Hakuna hatari zozote zinazoweza kukupata kwa kushiriki kwenye utafiti huu. Waweza kumuuliza mhojaji maswali yoyote wakati wote wa utafiti.

Kama una maswali yoyote kuhusu utafiti huu au kuhusu haki zako kama mshiriki, waweza kuwasiliana na Dr. Suleiman Kimatta kwa namba ya simu 0684606108. Ikiwa una maswali yoyote kuhusu haki zako kama mshiriki wa utafiti tafadhali wasiliana na Taasisi ya Taifa ya Tafiti za Kitabibu kwa S.L.P 9563, Dar es salaam au kwa simu +255-22-2121400.

## 9.4 Informed Consent Form in Kiswahili


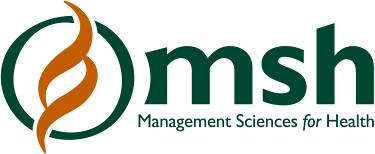


**Fomu ya Ridhaa kwa wahojiwa**

**Jina la utafiti: Kuboresha uzingatiaji na uendevevu wa kuwepo katika tiba za ARV kwa katika kliniki za afya ya uzazi na mwana zinazotoa huduma ya Option B Plus Tanzania**

Mahojiano yatachukua muda wa saa moja na­­­­­­­ yataongozwa na_____________.

Wakati wa mahojiano haya utaulizwa maswali kuhusiana na uzoefu wako kama mtumishi wa afya katika ngazi ya wilaya, mtoa huduma katika kituo cha kutolea huduma ya afya au mama anayetumia dawa za ARV.

Kuweka sahihi (au kuweka alama ya X) kwenye fomu hii kutaonyesha kuwa umefahamishwa kuhusu utafiti huu na ushiriki wako.

Mimi____________________ nimesoma karatasi yenye taarifa kuhusiana na utafiti huu (tumezungumza na mtafiti kuhusu taarifa hii kwa mdomo) na nimeambiwa kuhusu kile kinachohitajika kutoka kwangu na kile kitakachotokea kama nitashiriki kwenye utafiti huu. Hususan, nimesoma karatasi ya taarifa ya ujumla inayoeleza kuhusu utafiti, taasisi inayoufadhili na masharti ya kushiriki kwangu, nimepewa nakala ya karatasi ya taarifa kwa ajili ya kumbukumbu.

Nimeambiwa kuwa mahojiano yatachukua takriban saa moja kukamilika.

Maswali yangu kuhusiana na utafiti huu yamejibiwa.

Nimeambiwa kuwa kwa wakati wowote naweza kujiondoa kwenye utafiti huu pasipo kutoa sababu yoyote

Nimeambiwa kuwa baadhi ya mambo nitakayosema yanaweza kunukuliwa kwenye ripoti za matokeo ya utafiti huu lakini hii itafanyika kwa namna ambayo haitahusishwa na mimi.

Naridhia kushiriki kwenye utafiti huu.

Sahihi ya mhojiwa(au alama ya X) ______________________ Tarehe: __________________

Jina kamili la mhojiwa ___________________________

Sahihi ya mhojaji_______________________________ Tarehe: __________________

Jina kamili la mhojaji ___________________________

Jina kamili la shahidi ___________________________ Tarehe: __________________

Sahihi ya shahidi ____________________________

Utafiti huu umeidhinishwa na :

National Health Research Ethical Review Committee

National Institute for Medical Research

2448 Ocean Road

P.O.Box 9653

Dar es Salaam-Tanzania

Msismamizi mkuu wa utafiti

**Dr Suleiman Kimatta**

MSH

Tanzania

# 10.0 CURRICULUM VITAE OF RESEARCHERS

## 10.1 Dr. Suleiman Kimatta

**Suleiman KIMATTA**

*MSH Country Representative, Tanzania*

**Key Qualifications**

Dr. Suleiman Said Kimatta, MD, MMed (paediatrics) is currently the Management Sciences for Health's (MSH) Country Representative for Tanzania. Dr. Kimatta works as MSH Tanzania's Senior Technical Advisor for the Centre for Pharmaceutical Management of Management Sciences for Health. He has over 28 years of experience with maternal and child health programming at institutional and community levels. Prior to his role with MSH, Dr. Kimatta worked as the Regional Medical Officer (RMO) in the Southern region of Tanzania in Mtwara for four years, and then with UNICEF as their National Project Officer for IMCI, Malaria, EPI, and Nutrition from 1998 to 2006. He has also worked as a Zonal pediatrician, coordinator for the acute respiratory infections (ARI) and diarrheal disease programs. He has also served as a World Health Organization advisor on how to conduct trainings related to control of diarrheal disease and ARI programs to District Health Management Team members. He has an extensive international experience, mostly specializing in maternal and child health.

Dr. Kimatta has a plethora of research experience and publications and has consulted with various organizations. He received his Doctor of Medicine and Master's degree in Pediatrics from the University of Santiago de Cuba; he also has a diploma in the Spanish language from the International School of Languages in Cuba. Dr. Kimatta has received training in community planning in Anglophone African countries, Program Policy Planning (PPP), and other Managerial courses. He speaks fluent Hehe (a local language), Swahili, English, and Spanish.

**Education**

- Master’s Degree in Pediatrics, School of Medical Science, University of Santiago de Cuba, Cuba, 1986.
- Doctor of Medicine, School of Medical Science, University of Santiago de Cuba, Cuba, 1982.
- Diploma in Spanish language, International School of Languages, Havana, Cuba, 1976.

**Professional Experience**

**Management Sciences for Health, 2006–Present**

**MSH Country Representative and Senior Technical Advisor (2012 to date)**

- Ensure that MSH activities and management operations in country are implemented as per MSH mission, values, standards and local laws and regulations
- Facilitate execution of internal/external financial operations, and program reviews or audits with timely follow up to reviews or audit conclusions and recommendations
- I manage 23 staff who work in 4 projects, and I directly supervise 9 of the staff.
- Provide support, coaching and mentoring to staff I supervise in the implementation of the ADDO and Tibu Homa projects.
- Work to improve linkages and referrals among Tanzania’s public, private, and community health care providers.
- Work to introduce case management of pneumonia and diarrhoea in children through the ADDOs using amoxicillin dispersible tablets and co-packed Zinc and ORS and strengthened referral system between ADDOs and primary health facilities.
- Manage maternal and child health interventions within SDSI/ADDO and Tibu Homa projects

**Senior Program Associate, SPS-Child and Maternal Health, Tanzania (2006 to 2011)**

- Introduced and provided oversight the implementation of the child health/IMCI component in the ADDO program (ADDOs being used as platform for the implementation of public health intervention).
- Organized and developed training, advocacy, referral forms and monitoring tools for use in the ADDO program..
- Oversee formative research and the subsequent development of educational materials.
- Formulated and coordinated monitoring and evaluation systems for child health in the private sector (the ADDOs).
- Coordinated and facilitated meetings and liaised with TFDA,PC, MOHSW, USAID Tanzania, WHO, UNICEF and other collaborating partners.
- Prepared monthly, quarterly, annual and ad hoc reports for MSH/SPS, TFDA, MOHSW—IMCI Unit and USAID describing the progress towards program objectives with recommendations for change and remedial actions.
- Introduced an ADDO reporting mechanism which is in place and now being transformed into modern application of modern technology m-health through mobile phones.
- Coordinated strong partnership among organizations; T‐MARC, PSI, AED/POUZN and MSH/SPS together are working to strengthen district capacities in supervising ADDO program and incorporating ADDO interventions into the Comprehensive Council Health Plans (CCHP).
- Led the development of radio spots with messages that promote the services of ADDOs and the General Danger Signs these were broad casted by local radios.
- Conducted formative research and disseminated results at national, district and community levels.
- Disseminated of health information is being done through ADDOs.

**UNICEF, 1998–2006**

**National Project Officer, Health-IMCI, Malaria, EPI, and Nutrition, at National level but with focus in seven learning districts (Makete, Mbarali, Magu, Hai, Masasi, Mtwara rural and Kilosa)**

- Initiated and consolidated implementation of IMCI strategy at facility and community levels in the context of Integrated Early Childhood Development.
- Coordinated experts to develop tools for advocacy, training and monitoring of community IMCI.
- As IMCI master trainer, organized and trained 54 Trainers of Trainers of community IMCI.
- Piloted Discount Voucher System for ITNs to pregnant women in two districts which provided a learning ground for national scale-up.
- Initiated a system of dialogue on child and maternal health by Community Own Resource Persons (CORPs) at household level in seven learning districts.
- Put in place mechanisms that ensured vaccination coverage was raised in low performing districts through building capacities of district health personnel to detect low performing wards and focus in those areas to raise the vaccination coverage.
- Low performing districts raised vaccination coverage to above 90%.
- Discount Voucher System in place nationwide facilitating pregnant women and under‐five children to access ITNs.
- Community IMCI implemented in all districts of Tanzania though with varying strength.
- Advocacy, training, monitoring and IEC materials for CORPs available and widely in use and some of them used in the ADDOs.
- All districts plan and allocate funds for implementation of IMCI

**Ministry of Health and Social Welfare 1986–1998**

**Regional Medical Officer (RMO), Southern Region of Tanzania**

- Managed Health systems in a region of six districts with a total population of 1.1 million people.
- Supervised the development and implementation of district health comprehensive plans; utilized funds inclusive.
- Provided technical support to districts in the control of epidemics; meningitis, cholera, dysentery, measles etc.
- Coordinated work realized by NGOs and other partners including the private sector; in capacity as RMO, worked closely with the DED to establish a District Health Implementation Program for Mtwara region under the financial support of KFW. The program initially focused on improving the much dilapidated health facility infrastructure.
- All districts in the region managed to raised immunization coverage to 90%,.
- Epidemics were better managed shown by low case fatality rates, partners better coordinated and team work evident.
- Health staff well motivated.
- Health facility buildings and other infrastructures renovated/rehabilitated and a workshop for maintenance of hospital equipment established and functioning.

**Zonal Pediatrician, Southern Zone (Ruvuma, Mtwara and Lindi Regions)**

- Provided high technical expertise in managing childhood illness and provided on the job training to health staff on proper child care in three regions that constitute the southern zone.
- Provided supportive supervision to staff managing child health both in institutions and communities.
- Provided updates to staff working with neonates and infants.

**Southern Zonal CDD/ARI Program Coordinator (Ruvuma, Mtwara and Lindi Regions)**

- Developed plans for management of Diarrhea and Acute Respiratory Infections in the three regions that make the Southern zone.
- Coordinated the establishment of Diarrhea Treatment Corners in every health facility in the zone (DTC).
- Conducted supervision to health staff and established linkages with the CORPs.
- Developed manuals on CDD/ARI Program for use by Community Health Workers.
- Plans for CDD/ARI were developed and implemented resulting into lowering the diarrhea case fatality rates.
- Raised availability and use rate of ORS, less stock outs of ORS in health facilities, and DTCs established.

**Short-term Consulting**

- **European Commission, Location, March, 2010.** Reviewed health proposals for funding and provided technical Assistance to establish ACT outlets in Zambia.
- **WHO, Arusha/Tanzania, 1997 and 1998.** Temporary advisor in two inter country IMCI courses.
- **WHO, Ghana and Tanzania, 1993.** Temporary advisor in a course for intermediate and district level management of Disease Control Programs, WHO/AFRO.

## 10.2 Dr. Mwikemo Deborah Kajoka (PMTCT)


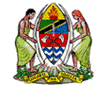


**Ministry of Health and Social Welfare**

**CURRICULUM VITAE**

**Summary**

**MWIKEMO DEBORAH KAJOKA MD, MPH**

Dr. Kajoka has 13 years of expertise and achievements in HIV and AIDS Program design, planning and management, relationship management, Business planning and strategy Health system strengthening, information and influencing policy change and general medical practice.

She is currently the National PMTCT coordinator since 2011 January responsible for leading the country towards achieving the elimination agenda of mother to child transmission of HIV then contributing to the national HIV and AIDS response.

Prior to this Dr. Kajoka was working at National AIDS Control Programme as the head of unit for four years leading the team in scale up of syndromic management of STIs and establishing youth friendly services, her leadership and technical oversight led to scale up of integrated of STI services across the country. She further participated in development of tools guidelines and SOPs for Various HIV interventions. From time to time Dr. Kajoka acted the office of Programme Manager NACP during his absence.

Prior to joining NACP on STI Head of Unit, She served as Liaison Officer to the STD project funded by Belgian Technical Cooperation of Belgian Government. Where she lead the executive of STI project in two regions that were not implementing syndromic management of STI for five years.

She also worked as a District Medical Officer for three years leading the Council Health Management Team in overseeing health Interventions in the district, and as advisor to the district authorities in matters pertaining health. She has been managing Cooperative Agreement under her portfolio for PMTCT under USG support, Global Fund and She also managed STI-BTC grant that was under Belgian Technical Cooperation for Belgian Government.

In terms of capacity building and career development, Dr Kajoka graduated Medical School and further acquired Masters in public Health. In addition she attended various short courses on E- learning course on Basics of Health Economics, 2013 – World Bank Institute (WBI), Professional Certificate in Strategic Health planning, 2012 – London UK, Monitoring and Evaluation of HIV programme, 2009 - Pretoria SA, Behavior Change Communication Strategy for Reproductive Health, 2007 – Tokyo Japan, Management and Administration Training of Trainers course for HIV/AIDS Programme managers 2007 – ESAMI Arusha, Action Oriented Research Methodology 2003 – PHC Iringa and Advance course from managing Diseases to Managing Health Systems 2003 – Bagamoyo. She also attended various International Global AIDS and African ICASA meetings and presented works in some occasions.

She is currently a PI of the soon to be concluded study on Integration of STI diagnosis, Treatment and Prevention in HIV clinical care prevalence and etiology survey (a collaborative work with MoHSW, ICAP and Columbia et all).

Dr. Kajoka is fluent in English, Swahili; under computer literacy she has good knowledge of Word, Excel, Power point and Internet.

**Full Name**: Dr. Mwikemo Deborah Kajoka

**Date of Birth**: 12^th^ December 1962

**Place of Birth**: Kyela - Mbeya Tanzania

**Sex:**  Female

**Nationality**: Tanzanian

**Profession**: Medical Doctor (MPH)

1. **EDUCATION:**

**1.1 Masters in Public Health**

Nuffield Institute – Leeds University in the United Kingdom 2001

- 1. **Medical Degree doctor**

Luganski State Medical University in Ukraine 1995

- 1. **Advanced level Certificate of Secondary Education examination (ACSSE)**

Mwenge Secondary School 1984

- 1. **Certificate of Secondary Education Examination (CSSE)**

Nganza Secondary school 1981

**2.0 EMPLOYMENT**

**2.1 National PMTCT coordinator**

PMTCT Programme, RCHs, MoHSW 2011 (Feb – To date)

**2.2 Ag Programme Manager**

National AIDS Control Programme 2011 (Jan – Feb)

**2.3 Head STI Unit:**  2007– 2010

National AIDS Control Programme

**2.4 Liaison officer** 2003 – 2007

Project “Support to STD Component of the NACP”

funded by the Royal Government of the Kingdom of Belgium.

**2.5 District Medical Officer** 2001- 2003

Mbarali District Council

**2.6 Medical Officer In-charge** 1997 - 2000

Mbozi District Hospital and Kyela District Hospital

**3.0 PROFESSIONAL EXPERIENCE**

- 1. **National PMTCT Coordinator: 2011 –To date**

1. National coordinator for Prevention of Mother to Child Transmission of HIV (PMTCT) services in the country
2. Coordinating the implementation of the Global Plan for Elimination of New HIV Infection Among Children by 2015, and Keeping their Mothers Alive
3. Provided guidance in the review of the National PMTCT guidelines to align with WHO 2012 recommendations
4. Led the Country team to develop a strategic plan for elimination of Mother to Child Transmission of HIV (eMTCT - Plan), 2012 – 2015
5. Provided leadership and technical oversight to the development, publication and dissemination of the Bottleneck Analysis for PMTCT 2011

- 1. **Head STI Unit: 2007 -2010**
  2. Overall coordinator for Clinical STI services in the country.
  3. Participated in planning, Budgeting and implementation of the Sexually Transmitted Disease (STD) programme activities under Global Fund (GF) and Medium Term Expenditure Framework (MTEF)
  4. Assisted and periodically worked as Ag.Programme Manager at National AIDS Control Programme (NACP) in the absence of the Programme Manager (while he was performing other official duties out of his work station up-country and internationally).
  5. Chairperson for Human Resource Committee of the Task force that was responsible for overseeing the implementation of the campaign all over the country during the National HIV testing campaign (2007)

Contributed in the development of Health Sector HIV/AIDS Strategic Plan II (2008-2012)

- 1. Led the team to develop the National Essential Health Sector HIV Intervention Package (2009)
  2. Participated in review of STI National Guidelines and the Manual for service providers
  3. Organized and participated in different trainings of health care workers in the country including :STI syndromic case Management and Reproductive Tract Infection, Syphilis screening of Antenatal mothers at Reproductive and Child Health (RCH) clinic and Adolescent Sexual Reproductive health friendly services

**3.3 Liaison officer 2003 – 2007** for the project “Support to STD Component of the National AIDS Control Programme” funded by the Royal Government of the Kingdom of Belgium.

1. Liaise with the Ministry of Health and Social Welfare, Belgian Technical Cooperation (BTC), Regional and District Health Management Teams in the implementation of the STD project.
2. Participated in planning, Budgeting and implementation of the STD project activities for the two project regions (Kilimanjaro and Ruvuma)
3. Implemented financial controls of the project
4. Organized and participated in different trainings of health care workers/communities (under the project) in area of

- STI syndromic case Management

- Syphilis screening of Antenatal mothers at Reproductive and Child Health (RCH) clinic

- HIV/AIDS/STI Peer Health Educators

1. Conducted supportive supervision to health facilities offering STI services to ensure its quality.
2. Participated in the Mid Term Review of the project in 2005 and Final Evaluation 2008
3. Constituted secretariat of the steering committee “Joint Local Partner Committee” (JLPC)
4. Prepared and submitted supervision, progress and annual reports to the NACP/BTC and to the steering committee.

**3.4 District Medical Officer in Mbarali district – Mbeya (2001- 2003)**

1. Constituted the chairperson of Council Health Management Team responsible for all health matters in the district
2. Provided advice to District Executive Director on health issues in the district and interpreted directives from Ministry of Health into action.
3. Developed Comprehensive Council Health plan and coordinated its implementation.
4. Participated in the preparation of the Council Budget (MTEF), financial and human resource management
5. Conducted Supervision to health facilities in the district
6. Participated in the promotion of health facilities utilization
7. Assisted the communities to improve their health conditions through sensitization, involvement in implementing activities under different programmes and projects such as Malaria, HIV/AIDS, TB and Leprosy, IMCI and EPI.
8. Prepared quarterly and annual reports which were submitted to District Executive Director (DED), RMO and to then Ministry of health

**3.5 Medical officer in-charge 1997 – 2000 at Vwawa-Mbozi and Kyela District Hospitals in Mbeya region.**

1. As a member of the Hospital Management Team, I participated in administrative and decision making work.
2. Executed daily medical duties at Out Patient Department (OPD) and In Patient Department (IPD).
3. Periodically worked as ag.DMO on his absence

**4.0 SHORT COURSES AND CONFERENCES**

**4.1 Trainings**

- E-learning course on Basics of Health Economics Cohort 4, 27^th^ Mar – 30^th^ Apr 2013
- Professional Certificate in Strategic Health Planning, International Centre for Parliamentary Studies, London UK, 2^nd^ -5^th^ July 2012
- Monitoring and Evaluation of HIV programmes, PAN African Training –Pretoria, 3^rd^ – 29^th^ May 2009
- Workshop on STIs Case Management Skills, Bangkok Thailand, 24 – 28 Nov2008
- Behavior Change Communication (BCC) Strategyfor Improving Reproductive Health Status for
- African Countries, JICA- Tokyo Japan, Nov 22, 2007 to 12^th^ Dec 2007
- Management and Administration Training of Trainers, Course for HIV/AIDS Programme Managers ESAMI, Arusha, 16 Apr – 4^th^ May, 2007
- Orientation programme on Adolescent Health for Health Care Providers organized by MOHSW and WHO, 13^th^ – 18^th^ Dec, 2004
- Action Oriented Research Methodology Course organized by Ministry of Health from 13^th^ – 31^st^ Jan 7^th^ – 25^th^ July 2003
- Advanced course From Managing Diseases to ManagingHealth Systems – Bagamoyo Tanzania, 5^th^ – 17^th^ Oct 2003

**4.2 Conferences**

- The XIX International AIDS Conference in Washington DC, USA, 22^nd^ – 27^th^ July 2012
- XVI International Conference on AIDS and STIs in Africa, (XVI^th^ ICASA) in Addis Ababa Ethiopia, 4^th^ – 8^th^ Dec 2011
- XVIII International AIDS Conference in Vienna, Austria, 18^th^ – 23^th^ July 2010
- International Conference on AIDS and STIs in Africa,(XV^th^ ICASA) in Dakar Senegal, 3^rd^ to 7^th^ Dec 2008
- International Conference on Community Health in,the African Region in Addis Ababa Ethiopia, 20^th^ - 22^nd^  Nov 2006
- Hormonal Contraception and HIV:Science and Policy: Africa Regional Meeting, 19^th^ – 21^st^ Sept 2005
- The XV International AIDS Conference in Bangkok, Thailand 11^th^ – 16^th^ July 2004

**5.0 STUDIES**

- Thesis: Determinants of Transmission of HIV among Adolescent in Mbeya region : Mbeya experience 2000 (MPH)
- PI for a study : Integration of STI diagnosis, Treatment and Prevention in HIV Clinical Care , a prevalence and Etiology Survey (collaborative work with MoHSW, ICAP, Columbia et all)

**6.0** **SKILLS AND COMPETENCES**

**Language**

Fluent in English, Swahili and Russian languages

**Computer Literacy**

Good knowledge of Word, Excel, Power point and Internet

**Leadership**

Supervising ABC Fellows from (MUHAS) participating in Leadership course and attached at PMTCT

## 10.3 Mrs. Salama Mwatawala

Salama Mwatawala

P.O.Box 75352, DSM

Mobile number (+255) 683500550 or (+255) 754855065

Email: [smwatawala@msh.org](mailto:smwatawala@msh.org) or ssmwatawala2004@yahoo.ca

**SUMMARY**

Pharmacist and public health specialist with seventeen years’ experience in pharmaceutical related field both in private and public sectors. Have experience in supporting pharmaceutical management programs related to HIV/AIDS, Malaria, TB and other essential medicines as well as management of public health related projects funded by different international organizations and institutions such as USAID, Bill & Melinda Gates Foundation and SIDA. Have successfully coordinated planning, implementation, and evaluation of several capacity building initiatives targeting pharmaceutical personnel in both private and public sector. Skilled in coordinating activities related to monitoring safety of medicines, conducting operational researches and rapid assessments and performance monitoring of pharmaceutical management systems.

**EXPERIENCE**

**12/2012 to date: Senior Technical Advisor,USAID Systems for Improved Access to Pharmaceuticals and Services (SIAPS) , Management Sciences for Health**

- SIAPS regional technical advisor for TB pharmaceutical management-Africa region
- Act as SIAPS focal person for Global Drug Facility in Anglophone countries responsible for supporting monitoring missions where need arises, provide technical assistance in implementation of early warning system to prevent stock out or wastage of TB medicines and providing technical assistance to selected TB programs in addressing procurement bottlenecks.
- Provided support in piloting and developing training package for Quan TB software used by GDF in quantification of TB medicines and as early warning system.
- Facilitated two applied quantification workshops using Quan TB software in November 2013 involving Kenya, Uganda and Zambia and February 2014 involving Tanzania, Zimbabwe and Ethiopia.
- Conducted GDF monitoring missions to Kenya, Tanzania, Uganda, Zambia and Zimbabwe and supported quantification of TB medicines and monitoring performance of pharmaceutical management system for TB.
- Participated in external evaluation of the National TB Control Program in Nigeria, Kenya, and Uganda as pharmaceutical and supply chain management (PSM) technical lead as well as Tanzania as local expert
- Participated in the development of early warning indicators to prevent stock out of TB medicines and assessing feasibility of collecting EWS data in two countries - Nigeria and Tanzania. The activity is being implemented by SIAPS in collaboration with GDF.
- Participated in the 2^nd^ Africa regional conference on management of TB medicines organized by MSH/SIAPS in collaboration with GDF and WHO –December 2012. The conference focused on identifying weakness and solutions in the TB supply chain system and developing an action plan to address main challenges based on the country specific context, 13 countries attended meeting.
- Involved in the development of training package for TB pharmaceutical management and facilitated the 5 day regional training workshop for TB supply chain managers from 8 African countries held in Swaziland, organized by MSH/SIAPS.
- Supported piloting of interventions related to engagement of private retail pharmaceutical outlets in TB case finding through strengthening linkage with TB diagnostic centers in Tanzania.

**9/2011 to 12/2012** Support implementation of Sustainable Drug Seller Initiative Program (SDSI) to ensure maintenance and sustainability of the ADDO program through strengthening regulatory authority, increase product availability aimed at ensuring good quality of services and profitability.

- Worked with other team members to prepare scope of works for consultants, budget review , review of assessment tools and monitoring implementation of contracted activities under Bill and Melinda Gates funded project-SDSI

**12/05 to 08/11: Senior Program Associate, USAID Rational Pharmaceutical Management Plus (RPM Plus) and later for Strengthening Pharmaceutical System Project (SPS), Management Sciences for Health**.

Under this capacity, I have worked in various assignments related to provision of technical support in planning; implementation, monitoring, and evaluation of selected interventions geared towards strengthening pharmaceutical management systems and improve performance of pharmacovigilance systems. The activities included providing support in the following areas:

*A: National-Level Technical Coordination and Planning with the MoHSW, Other Implementing Partners and Overall Program Management.*

- Participated in national level working groups to support strengthening supply chain and pharmaceutical management system for HIV/AIDS and PMTCT commodities, providing technical support in planning and implementation of different interventions.
- Actively participated in coordination meetings between MSH/SPS and USAID, with other USG implementing partners and maintained regular close contact with national and district level counterparts involved in pharmaceutical management for HIV/AIDS (NACP), PMTCT, Malaria, as well as medicines safety monitoring issues with TFDA.
- Contributed in workplans development, preparation of semi-annual and annual PEPFAR progress reports, quarterly reports as well as ad hoc reports and presentations to be delivered to MOHSW, NACP, TFDA, USAID and other stakeholders related to strengthening the operational capacity of health facilities, particularly in the pharmaceutical management and rational use of HIV/AIDS related medicines.
- Provide oversight role and guidance to three Program Associate staffs to ensure good performance in the implementation of planned activities on pharmaceutical management for HIV/AIDS commodities.
- Prepare scopes of work, and budgets for assigned activities in line with USAID and other donors’ regulations and policies.

*B: Supply Chain and Pharmaceutical Management Related Activities*

- Provided technical assistance in developing various rapid assessment and performance monitoring tools and conducted several assessments aimed at evaluating performance of pharmaceutical and supply chain systems including those related to ART and PMTCT.
- Coordinated the assessment of medicines safety monitoring systems and performance in Tanzania as part of the multi-country study involving 46 Sub Saharan African countries
- Provided technical support to Tanzania Food and Drug Authority in the review of national guidelines for monitoring medicines safety, ADR reporting tools, medication error reporting forms, quality monitoring tools as well as review of pharmacovigilance training package
- Involved in the development/review of various pharmaceutical management training packages including those related to national ARV and HIV test kits logistics/ supply chain management system, rational use of HIV/AIDS related medicine, management of antimalarial medicines and pharmacovigilance training package as well as participating in training of pharmaceutical personnel
- Supported the National AIDS Control program in the development of standard operating procedures (SOPs) and Job aids for dispensing practices, pediatric dosing charts, and medication use counseling.
- Provided supervisory support to ART sites to improve inventory management, data capturing, ordering and reporting as well as ensuring appropriate use of HIV/AIDS Medicines and other supplies.
- Provided technical support to National Malaria Control Program in changing malaria policy from SP to ACT as part of the national Artemisinin-Based Combination Therapy (ACT) technical working group. Responsibilities: support development of procurement plan, operational plan for storage and distribution of ACTs, tracking antimalarial consumption rate as well as tracking flow of subsidized ACTs distributed through Accredited Drug Dispensing Outlets (ADDOs).

**Research Experience**

**1/08- 6/11** **MSH/TZ focal person for Swedish International Development Aid (SIDA) International Network for Rational Use of Drugs-Initiative on Adherence to Antiretroviral (INRUD-IAA) project.**

- In collaboration with the National AIDS Control Programme, coordinated implementation of the pilot ART adherence study titled ‘’Strengthening Appointment and Patient Tracking Systems for Patients on Antiretroviral Therapy in Tanzania.
- Supported the design of pilot interventions, proposal development, developing tools for tracking ART patients’ attendance and tracing defaulters, training of health care providers in participating facilities and provision of oversight role in the monitoring and evaluation of the project, data analysis and in report writing.
- Supported MOHSW/NACP in adaption of the tested adherence intervention for scale up countrywide by providing technical support in reviewing the tools developed under ART adherence study by relevant stake holders, development of participants reference guide and trainers guide and coordinated training of trainers for the adapted appointment and patient tracking tools. The tools are now part of national HIV/AIDS M&E system.

**08/06-10/08**: Coordinated piloting of Monitoring, Training and Planning (MTP) approach in Tanzania, a capacity building initiative to strengthen pharmaceutical management in collaboration with Muhimbili University College of Health and Allied Sciences/Regional Training Resource Collaboration (RTRC).

**10/04-9/05 Pharmacist In charge and Managing Director of Sabrina Pharmacy**

- Responsible for overall management of Sabrina pharmacy with over 120 million shillings annual sales turn over. Involved in supervision of staff, office operations, finance management, and decisions making on issues of procurement, storage, and distribution of pharmaceuticals and other supplies.

**7/97-12/99**  **Pharmacist in charge of Bahari Pharmaceutical Company Limited**

- Overseeing all activities related to procurement, inventory management, rational use of medicines and all activities related to professional ethics.

**EDUCATION**

**10/04-9/05** Master of Public Health (MPH), University of Dar es Salaam

**10/92-07/96** Bachelor of Pharmacy (BPharm), University of Dar es Salaam

**Professional Training**

**11/2007** Pharmaceutical Management for Technical Assistance Course organized by MSH Cape Town, South Africa.

**7/2007** Management Development program for HIV/AIDS Managers organized by Johnsons &Johnsons/University of California Los Angeles (UCLA), Nairobi Kenya.

**1/ 2006** Regional HIV/AIDS Pharmaceutical Management course, organized by MSH, Dar-es Salaam Tanzania.

**11/ 2005** Regional Pharmaceutical Management for Malaria Medicines training course, organized by Management Sciences for Health (MSH), Dar-es Salaam Tanzania.

**7/96-7/99** Muhimbili National Hospital; A twelve months apprenticeship in hospital drug management.

**International Workshops and Conferences.**

**11/2014** Attended 45^th^ Union World Conference on Lung Health in Barcelona Spain and made presentation one presentation.

**11/2013** Attended the 44^th^ Union World Conference on Lung Health in Paris France from 28^th^ Oct to 4^th^ November, 2013 and made two presentations

**11/2013** Attended the 43^rd^ Union World conference on Lung Health in Kuala Lumpur Malaysia from 13^th^ to 17^th^ November, 2012

**11/2012** Participated in the 2^nd^ Africa regional conference on management of TB medicines organized by SIAPS in collaboration with GDF and WHO –December 2012 in Zanzibar Tanzania. The conference focused on identifying weakness and solutions in the TB supply chain system and developing an action plan to address main challenges based on the

**5/2013** Participated in the 5 day regional Support Officers’ (RSO) workshop organized by the Global Drug Facility (GDF) in Geneva from April 29^th^ to May 3^rd^ 2013.

**11/2011** Presented at the third international conference for improving use of medicines ICIUM held in Antalya Turkey from 14^th^ to 18^th^ November 2011.

**07/ 2010** East African regional meeting for analyzing data on interventions to improve adherence to Antiretroviral (ARV) held in Nairobi Kenya organized by INRUD-IAA.

**6/2010** Global Technical Staff Meeting for MSH/Strengthening Pharmaceutical System, a USAID funded Program held in Arlington, United States.

**11/2009** Attended 3rd Annual East African INRUD Meeting on Adherence to and Retention of Patients on Antiretroviral, Gisenyi, Rwanda

**11/2009** 5-day workshop on medicines safety monitoring for ART program organized by World Health Organization (Geneva) targeting regulatory authorities, and other partners aimed at building capacity in conducting pharmacovigilance activities.

**06/2009** Global Technical Staff Meeting for MSH/Strengthening Pharmaceutical System, a USAID funded program held in Arlington, United States.

**11/2008** Participated in the Second Annual East African INRUD Meeting on Adherence to and Retention of Patients on Antiretroviral (ARV) Therapy in Addis Ababa, Ethiopia.

**Membership in Professional Institutions /Societies**

- Registered with Pharmacy Council (Tanzania)
- Member of Pharmaceutical Society of Tanzania (PST)
- Chairperson and member of the National Committee of Medicines Safety Committee of Tanzania Food and Drug Authority.
- Member of National TB Supply Chain Management committee
- Treasurer and founder member of Tanzania Association of Women in Pharmacy Profession (TAWOPHA)
- Member of International Network for Rational Use of Drugs (INRUD)

## 10.4 Ms. Levina Lema

**Levina Lema**

**Personal Information**

Country of Birth: Tanzania

Place of Birth: Mwanza

Date of Birth: 14^th^ September 1974

Sex: Female

Marital Status: Married with four Children

Current Occupation: Strategic Information Officer

Ministry of Health and Social Welfare

6 Samora Machel Avenue

11478 Dar es Salaam, Tanzania

Phone: +255 22 2127176

Fax: +255 22 2127175

E-Mail: levlema@yahoo.com

**Education**

- M.A Statistics, University of Dar es Salaam 2005-2007
- B. A Statistics, University of Dar es Salaam 1996-1999
- Advanced Certificate of Secondary Education 1992-1995
- Ordinary Certificate of Secondary Education 1989-1992

**Other Training/Short Courses**

- Project Monitoring, Evaluation and Reporting, IDCA, Swaziland 9-23 October 2009
- Trainer of Trainer (TOT) Course in Prevention of Mother to Child

Transmission of HIV (PMTCT) 16-26 June 2008

- SAS Training (Introduction to SAS Programming, Survival Analysis,

using Proportional Hazards Model) Johannesburg South Africa 26-30 December 2007

- Tanzania PMTCT program management Workshop 12-16 November 2006
- Seeking Online Health information, INFORM In-service Training-

NIMR. HQ 4-6 December 2006

- IMHOTEP 2004 Internship project, MOREHOUSE college, Atlanta USA 24^th^May-6^th^August 2004
- Workshop on the conduct of GCP-Compliant TB/HIV, Kampala Uganda 20-14, October 2003
- Training on Health System Management Course, Bagamoyo 05-17 October 2003
- Training course in EPI Info 2002, Epidemiological Investigation,

Harare, Zimbabwe 21-24 July 2002

**Experience**

- Strategic information Officer, Ministry of health and Social Welfare Reproductive and Child Health, PMTCT Unit August 2007 To date
- Research Scientist, National Institute for Medical Research, Muhimbili Medical Research Centre 1999-July 2007

**Publications/Research Experience**

- HIV Early Infant Diagnosis (HEID)Monitoring System in Tanzania
- Determinant of high risk sexual behavior leading to HIV/AIDS among youth in Kibaha district, Coast Region
- Evaluation of KEMR. I Hepcel II Test kit for detection of hepatitis B surface antigen
- Epidemiology of Tuberculosis of the spine in Same district, Tanzania
- Situation Analysis on the Active Management of the Third Stage of Labour in Tanzania
- Situation analysis for implementing Community –based TB Dots in Arumeru and Karatu districts
- Knowledge, Attitude and Practice towards tuberculosis among community members in Mbeya region
- Situation Analysis of the laboratories capacities and good laboratory practices in HIV testing
- Capacity of Public and Private laboratories in diagnosing HIV/AIDS related opportunistic infection in Tanzania
- Epidemiology of Infuenza A virus in Tanzania
- Costing study for HIV related laboratories in different zones in Tanzania
- Survey of Specimen transportation and archiving system in Tanzania

**PMTCT Work/Related Experience**

- PMTCT Trainer and Supervisor
- Responsible for supporting all strategic information, monitoring and evaluation, and performance reporting in country.
- Responsible for planning of and implementing all Monitoring and Evaluation activities in PMTCT program;
- Ensure availability of PMTCT data collection and reporting Tools at all levels
- Maintain PMTCT database
- Liaise with district and regions to ensure smooth flow of data to the national level.
- Conduct data audit visits at regional/district and at facility level
- Perform data editing and verification to ensure completeness and error free
- Liaise with Implementing Partners on the PMTCT monitoring and Evaluation activities including reviews and update of M and E tools, Guideline, SOP and training package
- Participate in training of both paper base and electronic PMTCT system
- Prepare quarterly, Semi-annual and Annual progress reports for HIV/AIDS, PMTCT
- Organize and facilitate PMTCT trainings (Refresher, Comprehensive and Monitoring and Evaluation)
- Organize and Facilitate RCH Zonal meetings
- Organize and Facilitate Annual Review meetings
- Coordinate Monitoring and Evaluation Sub team meetings
- Participate in the development of Health Sector Strategic Plan II for HIV and AIDS 2009-2013
- Participate in the development of PMTCT scale up plan to operationalize the Health Sector Strategic Plan II
- Participate in development of Essential PMTCT and Paediatric Planning package.
- Participate in the development of Health Sector Strategic Plan II for HIV and AIDS 2009-2013
- Participate in the development of Health Sector performance Profile
- Participate in the development of Tanzania Elimination of Mother To Child Transmission of HIV
- Participate in the development of National Community Strategy for Elimination of Child Transmission of HIV (eMTCT)
- Participate in the development of Integrated Supportive Supervision Tools
- Participate in development of District Health Information System (DHIS II) database
- Participate in MNCH MTR Qualitative Assessment
- Participate in Development of Care and Treatment reports
- Participate in developments of Annual Health Profiles
- Participate in development of District Health Profile Template

**Memberships**

- Member of Tanzania Public Health Association (THP)
- Member of secretariat of the national Task Force Team for Elimination of Mother to Child Transmission of HIV
- Members of the Maternal and New Born Child Health (MNCH) Mid Term Review (MTR) Task team
- Member of the National Evaluation Team (NEP)
- Member of the MOHSW Monitoring and Evaluation Team.

**Publications**

Lema,LA. Katapa, R.S &Musa, A.S(2008) HIV /AIDS and sexual behavior among youth in Kibaha district, Tanzania. Tanzania Journal of Health Research 10 (2), 79-83

Kilale A.M, Mfinanga GS, Kunda J, Lema L, Chonde TM, Mfaume S (2001): The Operational Implication of the Spot-Morning-Spot sputum collection for diagnosis of tuberculosis. Int J of Tuberc. Lung Dis Vol 5 no. 11 Nov 2001. Suppl 1 pg 236

Kilale AM, LEMA AL, Kunda J, Mhomisoli FP: Epidemiology of Tuberculosis in a tea farming area in Mufindi District,Tanzania. Health Research bulletin (2001) Vol 3, No 2.

A. Kahwa, GS Mfinanga, GD Kimaro, B. Mutayoba, E. Ngadaya, G. Mbogo, JW Ogondiek, AM Mdemu, VR Nyigo, L. Lema, S. Kivuyo and G. Mubius: Exploring the cost of laboratory testing for HIV and major source of funding for Tanzania: Research Journal of Medical Services 2 (1) 33-37, 2008

Prevalence of HIV among tuberculosis patients in an urban health facility in Dar es Salaam, Tanzania. Tanzania Health Research Bulletin Vol 5 2003: 56-60

Kilale A.M, Mushi A.K , Lema L.A., Kunda J, Makasi, CE, mwaseba, D., Range N.S & Mfinanga, G,S. (2008). Perception of tuberculosis and treatment seeking behavior in Ilala and kinondoni Municipalities in Tanzania. Tanzania Journal of Health Research 10 (2), 89-94

Mfinanga G.S, Ngadaya E, Kimaro , G. Mtandu R, Lema L.A, Basra D, Lwila F., Egwaga S. & Kitua A. Y. (2008). Capacity of health care facilities in the implementation of Direct Observed Treatment Strategy for tuberculosis in Arumeru and Karatu Districts, Tanzania. Tanzania Journal of Health Research 10(2), 95-98.

## 10.5 Mr. Jafary Liana

**Jafary H Liana**

P.O.Box 65256 Dar es salaam,

Mobile. No. +255 784 262981, +255658009908

Email: ; [jafaryl@yahoo.com](mailto:jafaryl@yahoo.com)

**Summary**

Pharmacist with fifteen years’ experience in Pharmaceutical Sector in Tanzania both private and public. Experience in managing private pharmaceutical business and programs related to access to essential medicines. Experiences with private and public supply chain management of health commodities, and have successfully coordinated planning, implementation, and evaluation of private sector medicine access initiatives. Skilled in coordinating activities related to rapid assessments and monitoring and evaluation of private sector pharmaceutical management initiatives.

**Experience**

**Management Sciences for Health, Sustainable Drug Seller Initiatives (SDSI) Senior Technical Advisor, Tanzania, 2011-Present**

- Technical lead for SDSI Tanzania, a Bill and Melinda Gates Foundation-funded program, which supports the maintenance and sustainability of the accredited drug dispensing outlet (ADDO) program. The ADDO program is a national public-private initiative that aims to improve access to essential medicines and quality pharmaceutical services in rural and underserved areas.
- Responsible for overall coordination of the program activities in partnership with the Pharmacy Council of Tanzania. Responsibilities include project management, activity and personnel supervision, and financial management.
- Provides technical oversight for all program sub-contractor activities, field activity implementation, operational research and evaluations, and meetings and workshops.
- Successfully coordinated and completed a comprehensive medicines access survey in four regions of Tanzania covered by the ADDO Program, which included facility and household surveys, mystery shopping, qualitative interviews, and laboratory testing on medicine quality. Responsibilities included overseeing and coordinating data collection teams and ensuring smooth operations of data collection process and results.

**Management Sciences for Health, East Africa Drug Seller Initiative (EADSI), Senior Program Associate, Tanzania, 2008 -2010**

- Oversaw and implemented activities related to the EADSI Program, a Bill and Melinda Gates Foundation-funded initiative for the ADDO program in Tanzania. The initiatives documented the ADDO model as a regional template approach to address access problems.
- Key responsibilities included coordinating project activities with government counterparts, organizing meetings and workshops, implementing field activities, and coordinating and supervising sub-contractors.

**Management Sciences for Health, Rational Pharmaceutical Management Plus (RPM Plus) and Strengthening Pharmaceutical Systems (SPS) Senior Program Associate, Tanzania, 2006-2008**

- Responsible for the implementation of the PEPFAR funded accredited drug dispensing outlet program scale-up in Morogoro region and integration of Home Based Care kit distribution through ADDOs.
- Provided technical support to PEPFAR funded ARV- Pharmaceutical Management at the facility level in collaboration with EGPAF.
- Provided technical support to Tanzania Food and Drugs Authority to scale-up the ADDO program in Tanzania.

**Management Sciences for Health, Strategies for enhancing Access to Medicines (SEAM) Program Consultant, Tanzania, 2004 -2005**

- MSH/SEAM program consultant providing supportive supervision for the ADDO program pilot in Tanzania.
- Key responsibilities included providing onsite supervision for ADDO dispensers on pharmaceutical regulations, dispensing practices, record keeping, and pharmaceutical stock management.

**Morocco Pharmacy & New Magomeni Pharmacy, Managing Director and Pharmacist in Charge, Tanzania, 2000 -2005**

- Responsible for overall management of retail pharmaceutical business including financial, staff, and pharmaceutical management.
- Managed inventory, including quantification, and procurement and storage of pharmaceutical and medical devices.
- Supervised dispensing activities and record keeping.

**UGMC Store (Uhuru Pharmacy), Pharmacist in Charge, 1998-2000**

- Responsible for overall supervision and management of all pharmaceutical related activities including procurement, storage and dispensing services.

**Education**

Bachelor of Pharmacy degree, University of Dar Es Salaam Tanzania 1996.

**Additional Coursework and Training**

- International Course in Applied Epidemiology, Emory University School of Public Health. Atlanta, USA, September 2011.
- Strategic Communications Training, Capetown, South Africa, October 2008.
- National Training of Trainers for ADDO Program Dispenser Training, Tanzania, June 2008.
- National Training of Trainers for HIV/AIDS Commodity Logistic Management, Tanzania, July 2008.
- Training on USAID Administrative and Financial Management of USAID awards, Tanzania, February 2007.
- Training on Assessments for Medicine Access, Ecumenical Pharmaceutical Network. Malawi, June 2005.
- Internship at Muhimbili National Hospital on hospital drug management. Topics included dispensing practices, compounding, production of intravenous fluids, storage procedures, and inventory management.

**Short Term Consultancy Assignments**

**Tanzania, MSH/EURO Health Group - April** **2007**

- Consultant from MSH in collaboration with Euro Health Group on Drug Tracking Study under the Ministry of Health and Social Welfare. The study focused mainly on identifying gaps in the pharmaceutical sector supply chain with a focus on commodities and financing.

**Liberia, MSH/SDSI – January 2013**

- Support to Liberia Medicines & Health Regulatory Authority (LMHRA) and Pharmacy Board of Liberia on capacity building of drug inspectors.

**Presentations to International Conferences**

- International Conference on Improving Access to medicines (ICIUM) – Antalya Turkey - November 2011
- Stakeholders consultation on informal health care providers – Chennai India – March 2013
- Improving Access to Essential Medicines for Mental, Neurological and Substance Use Disorders in Sub-Saharan Africa – Addis Ababa Ethiopia- January 2014

**Professional Affiliations**

Member; Pharmaceutical Society of Tanzania, Present

Treasurer; Pharmaceutical Society of Tanzania, 2005

Secretary**;** Pharmaceutical Society of Tanzania Dar es Salaam Branch, 2004

**Languages**

Swahili – Native, English – Fluent

**REFERENCES:**

**Dr. Romuald Mbwasi**

Lecturer, St John’s University

Tel No: 0784202234 or +255767202235

Email: [mbwasir@yahoo.com](mailto:mbwasir@yahoo.com)

**Dr. Suleiman Kimatta**

Country Representative – Management Sciences for Health (Tanzania office)

Telephone: +255 684 606108

Email: [skimatta@MSH.org](mailto:skimatta@MSH.org)

## 10.6 Dr. Angel Dillip

1

**ANGEL DILLIP**

**P.O.BOX 32892 PHONE +255 713 251518**

**DAR ES SALAAM EMAIL – adillip@ihi.or.tz/**

**TANZANIA angdil1@yahoo.com**

**PROFILE**

A social epidemiologist with diverse academic and field experience in public health research. Angel has been well involved in project designing, management, as well as monitoring and evaluation in social and human development discourses, especially development interventions in the grassroots, particularly community and rural development. For the past 10 years Angel has been immersed in public health related social research focusing on social underpinnings of ill-health, the social impacts of disease and illness and epidemiological implications of social behavior.

**EDUCATIONAL QUALIFICATION**

2008- June 2012: PhD (Sandwich Program), Public Health and Epidemiology, University of Basel, Switzerland

2002-2004: M.A. (Sociology), University of Dar es Salaam,

Concentrations: Advanced Sociological Theory, Methods and Techniques in Social Research, Medical Sociology and Anthropology, Gender and Population Studies, Urban Social Studies

1999-2002: B.A. (Sociology) (Hons)-University of Dar es Salaam,

Concentrations: Social Problems of Urbanization, Medical Sociology and Anthropology, Social Psychology and Counseling, Sociology of Religion, Social Science Research Methods, Race, Class and Ethnicity

**SKILLS**

• Microsoft Word, Excel

• Proficient with statistical and qualitative analysis software: SPSS, Stata and MAXqda

**EXPERIENCE**

***Research Scientist***- **June 2006-present**

*Ifakara Health Institute (IHI), Ifakara-Tanzania*

*July 2012- Present*

Work with Data Analysis Cluster (DAC). Main responsibilities include:

• Co-manage DAC junior members to produce output using secondary data

• Lead a component of DAC training for analyses and publication

• Provide expert consultancy on mixed methods analyses at the outset of project work

• Work with data archiving / data central and our juniors to manage data brought in by and generated by DAC consultancies so we have a nice data store (including SPD/CSS/DSS datasets) and ultimately hand them over for archiving

• Take responsibility for first authoring agreed publications (at least 2 in the first year)

• Work to build the list of DAC advisers-linking to the new circle of fellows unit

• Lend methodological (mixed methods) expertise to the PH MSc development headed by the training committee

***2006-June 2012***

Implement the ACCESS Malaria Program aimed at improving access to prompt and effective treatment and care for all Malaria episodes in children adults and pregnant mothers. Working on monitoring and evaluation component of the project, activities revolves around;

• Studies on morbidity and mortality

• Community survey on care seeking behavior

• Assessment of quality of care at health facilities

• Assessment of drug availability and quality of services in drug selling shops

• Data analysis and report writing.

**Assistant Program Officer February 05-May 2006**

*Mwalimu Nyerere Foundation, Tanzania*.

Responsible in;

• Concept and protocol development

• Implementation of research and projects

• Data analysis and report writing

• Monitoring and evaluation of intervention

**Researcher April 2004-January 2005**

*Global Network of Religions for Children (GNRC AFRICA), Tanzania*

Implement the ‘Education for peace program’. Activities revolved around;

• Monitor and review community based education for peace initiatives among children in Dar es Salaam and Zanzibar areas.

• Working with communities and NGOs in designated areas regarding issues touching on the children and youth in difficult circumstances determining how these situations impact, in both the long and short term, on the security and peace of the communities, tolerance and peaceful co-existence.

**Postgraduate Teaching Assistant October 2002- March 2004**

*University of Dar es Salaam, Department of Sociology, Tanzania*

Duties entailed;

• Facilitating seminar classes for undergraduate classes

• Marking tests, presentations and assignments

• preparing students coursework prior University exams

**RESEARCH/CONSULTANCIES**

**April-July 2013**

• Assessment of quality of dispensing practice by dispensers in Accredited Drug Dispensing Outlets (ADDOs) in Morogoro, Singida, Tanga and Mbeya. A study organized and funded by Management Science for Health (MSH) Tanzania

**May- September 2012**

• Situational and Optional analysis for collaboration between ADDOs and Community Based Health Initiatives (CBHI) in Kilombero and Pwani regions. A study organized and funded by Management Science for Health (MSH) Tanzania

**November-December 2010**

• Assessment of stakeholders’ satisfaction with decentralized ADDO implementation process in Sindiga and Mtwara regions. A study organized and funded by Management Sciences for Health (MSH) Tanzania.

**June-August 2010**

• Study on Referral Pathways to CCBRT for children born with impairments in Temeke and Rufiji districts. A study organized and funded by CCBRT Hospital

**March-May 2010**

• Trials of Improved practices (TIPs) on WASH (Water, Sanitation and Hygiene) in Dodoma Region. A study organized by T-MARC and funded by the Agency for Education Development.

**May-June 2009**

• Situational Analysis of HIV and AIDS among health care and social welfare workers in the health sector in Tanzania. A study organized by the Ministry of Health and Social Welfare, Tanzania

**May –June 2007**

• A review of Effectiveness of the Participatory Hygiene and Sanitation Transformation Strategy (PHAST) in four districts in Tanzania. An evaluation supported by WATERAID, UNICEF, WSP and the Tanzania Ministry of Health and Social Welfare

**February-March 2005**

• The Situation of Poaching In Tanzania-A Case Study of Selous Game Reserve in Ruvuma, Lindi, Morogoro and Coast region’ Organized by the Ministry of Natural Resource and Tourism-Dar es Salaam

**November 2003-December 2004**

• Health Seeking Behavior among Street Children in Tanzania, a case study of Ilala and Kinondoni districts-Dar es Salaam. MA Sociology dissertation under the supervision of Dr. A. Mvungi, department of sociology-UDSM

**April-May 2003**

• Environmental Impact Assessment in Zanzibar: organized by TASAF Funded Projects

**February- March 2003**

• Public Service Delivery Survey in Tanga Region’, organized by Economic and Social Research Foundation, (ESRF)

**September-October 2001**

• Socio-economic Effects of HIV/AIDS in Mwanga district, Moshi’. Organized by Research on Poverty Alleviation (REPOA)

**July-August 2001**

• Baseline Study (Survey) on HIV/AIDS under Area Development Project, Ngerengere district, Morogoro. Organized By World Vision, Tanzania.

**MEMBERSHIP**

• Member of the Partnership for social scientists in Malaria control (PSSMC)-A group of social scientists in Africa and the world aiming at contributing to malaria control through enhancing the appreciation and application of social science approaches to public health

• Member of the Medical Anthropology Research Group (MARG) – An Interdisplinary Group of Scientist at the Swiss Tropical Institute, the Institute of Social Anthropology and The African Studies Center of the university of Basel in Switzerland

**PRESENTATIONS AND POSTERS**

**1. Dillip A**, Alba S. Mshana C, Hetzel M, Lengeler C, Mayumana I, Weiss M, Mshinda H, Schultze A, Obrist B: Acceptability: A Neglected Dimension of Access to Health Care: Findings from a study on childhood convulsions in rural Tanzania*: Oral presentation at the American Society for Tropical Medicine and Hygiene, December 7 2011, Philadelphia, USA*

**2. Dillip. A,** M.W.Hetzel, D Gosoniu, F. Kessy, Lengeler C, I. Mayumana, C. Mshana, H. Mshinda, A. Schulze, C.Pfeiffer, M.G Weiss M, B. Obrist: Treatment seeking for fever and convulsion in Rural Tanzania. *Poster presentation to the Multilateral Initiative on Malaria (MIM), November 2009, Nairobi Kenya.*

3. **Dillip A**, Alba S, Hetzel M, Obrist B, Kessy F, Lengeler C, Schulze A, Mayumana I, Mshana C, Makemba A, Mshinda H: Malaria treatment in the private sector in rural Tanzania. *Poster presentation to the American Society of Tropical Medicine and Hygiene, 57th annual meeting, December 2008* New Orleans, United States

4. **Dillip A**, Hetzel M, Gosoniu D, Alba S, Lengeler C, Kessy F, Mayumana I, Makemba A, Mshana C, Schulze A, Mshinda H, Pfeiffer C, Weiss M, Obrist B: Treatment seeking patterns for fever and convulsion in rural Tanzania. *Oral presentation to the Swiss Society of Tropical Medicine and Parasitology, October 2008*

Switzerland

5. **Dillip A**, Hetzel, M. W., Lengeler C, Obrist B, Alba S J A. Makemba A et al: Shortages of Antimalaria in health facilities and shops in Kilombero and Ulanga districts-Implications for the Roll-Out of Arteminism-Based Combination Therapy (ACT). *Oral presentation to the 22nd Annual Joint Scientific Conference of the National Institute for Medical Research, 2007* Arusha, Tanzania

**PUBLICATIONS**

1. **Dillip. A,** Alba S, Mshana C, Hetzel M., Lengeler C,, I. Mayumana I, Schulze A, Mshinda H, Weiss M.G, Obrist B: Acceptability – A Neglected Dimension of Access to Health Care: Findings from a study on childhood convulsions in rural Tanzania. *BMC Health Services Research* 2012, **12**:113

2. Alba S, M.W.Hetzel, Goodman C, **Dillip A**, Liana J, Mshinda H and Lengeler C: Improvements in Access to malaria Treatment after switch to Artemisinin combination therapy and the introduction of accredited drug dispensing outlets- a provider perspective. *Malaria Journal 2010,* **9***:164*

3. Alba S, **Dillip A**, Hetzel M, Mayumana I, Mshana C, Makemba A, Alexander M, Obrist B, Schulze A, Kessy F, Mshinda H, Lengeler C: Improvements in access to malaria treatment in Tanzania following community, retail sector and health facility interventions-a user perspective. Malaria Journal 2010, **9**: 163

4. **Dillip. A,** M.W.Hetzel, D Gosoniu, F. Kessy, Lengeler C, I. Mayumana, C. Mshana, H. Mshinda, A. Schulze, C.Pfeiffer, M.G Weiss M, B. Obrist: Socio-cultural factors explaining timely and appropriate use of health facilities for *degedege* in south-eastern Tanzania. *Malaria Journal 2009,* ***8****:144*

5. Hetzel, M. W., **A. Dillip,** C. Lengeler, B. Obrist, J.J. Msechu, A. Makemba, C. Mshana, A. Schulze, H. Mshinda (2008) Malaria treatment in the retail sector: Drug sellers’ knowledge and practices in rural Tanzania. *BMC Public Health 2008;* ***8****:157*.

6.Hetzel, M. W., N. Iteba, A. Makemba, C. Mshana, C. Lengeler, B. Obrist, A. Schulze, R. Nathan, **A. Dillip**, S. Alba, I. Mayumana, R.A. Khatib, J.D. Njau, H. Mshinda. (2007) Understanding and improving access to prompt and effective malaria treatment and care in rural Tanzania: *The ACCESS Project. Malaria Journal 2007*

*Jun 29;* ***6****(1): 83.*

7.Hetzel, M. W., N. Iteba, C. Lengeler, B. Obrist, **A. Dillip**, S. Alba, A. Makemba, C. Mshana, A. Schulze, H. Mshinda (2007) Improving access to prompt and effective malaria treatment: better drugs are not enough. *Tropical Medicine and International Health* ***12****(s1): 32-33.*

8.Obrist, B., N. Iteba, C. Lengeler, A. Makemba, C. Mshana, R. Nathan, S. Alba, **A. Dillip**, M. Hetzel, I. Mayumana, A. Schulze, H. Mshinda (2007) Access to Health Care in Contexts of Livelihood Insecurity: *A*

*Framework for Analysis and Action. PLoS Medicine October 2007;* ***4****(10):1584-1588.*

## 10.7 Dr. John Chalker

**John C Chalker**

**KEY QUALIFICATIONS**

Dr. John Chalker is a UK-trained physician with a Diploma in tropical medicine and hygiene, a Master’s degree in Community Health in Developing Countries and a Doctorate in Health Systems Research.Dr. He is a Principal Technical Advisor for Management Sciences for Health based in England. He has recently completed coordinating a five year Swedish funded program to learn how to monitor and improve adherence to antiretroviral medicine in East Africa. He has responsibility for coordinating the International Network for the Rational Use of Drugs (INRUD) and for providing technical support to CPM Program in areas related to promoting rational medicine use. He has more than 25 years of international experience, in designing, implementing, and managing health development projects and quality improvement interventions in a wide range of resource poor countries in Africa, Asia and the Middle East, including managing projects in Yemen, Vietnam, and Burma. He served as a WHO Technical Officer in Malawi and spent two years as medical director for the Britain-Nepal Medical Trust in Nepal.

**EDUCATION**

Karolinska Institute, Stockholm. Health Systems Research, December 2003, PhD

London School of Hygiene and Tropical Medicine, Community Health in Developing Countries, 1991-2, MSc(Med).

Liverpool School of Tropical Medicine, Diploma of Tropical Medicine and Hygiene, 1986, DTM&H.

University of Bristol, Medicine, 1980-85, MB ChB.

University of Sussex, School of African and Asian Studies, Social Psychology, 1968-72 BA (Hons)11i.

**PROFESSIONAL EXPERIENCE**

July 2003 – present Management Sciences for Health, Arlington VA

Principal Program Associate / Principal Technical Advisor. He is now the chief advisor for a 3ie funded project to improve adherence to antiretroviral medicine by pre and post natal HIV positive women attending reproductive health clinics in Tanzania. He was the Coordinator of the International Network for the Rational Use of Drugs and project director of the Swedish funded 5 year adherence to antiretroviral medicines program in East Africa which started in September 2006. He was Chair of the International Organizing Committee for the Second (2004) and Third (2011) International Conferences for Improving the Use of Medicine. He also facilitated a number of courses for Drugs and Therapeutics Committees, Promoting Rational Drug Use and Managing Drug Supply.

October 2000 – July 2003 Management Sciences for Health, Arlington VA

Technical Coordinator. As Global Technical Coordinator for the USAID-funded RPM Plus, he provided leadership and technical support on combating antimicrobial resistance, to child health and malaria interventions. He initiated the development of a community assessment tool to understand private-sector services for malaria and IMCI. He was also the Coordinator of the International Network for the Rational Use of Drugs.

February 2000 – October 2000 Management Sciences for Health, Arlington VA

Senior Program Associate, Drug Management Project. During this time the required role was to take on any outstanding tasks at the wrap up of the seven year rational pharmaceutical management project.

April 1996 – April 2000 London School of Hygiene and Tropical Medicine

UK Coordinator of a multi-disciplinary controlled intervention study, in collaboration with the Karolinska Institute, in Thailand and Vietnam, to find ways of improving good pharmacy practice, including private pharmacist’s prescribing and dispensing practices.

August 1999 to January 2000 Elsevier Science, Biomedical Division, Amsterdam

Sub-editor for Meyler’s side effects of drugs, 14th edition, published during 2000.

1996 April- 1999 April: Euro Health Group & Dutch Government in Yemen

Team leader on the Yemen Drug Action Program:This four year programme’s aim was to nationally improve the rational use of drugs and to establish a national drug policy, an essential drug list and use of treatment guidelines as well as to effect legislation, registration, inspection, quality control, procurement and distribution logistics.

1993 December - March 96 for SCF UK in Vietnam and the Health Policy Unit, London School of Hygiene and Tropical Medicine

Project Manager and Visiting Research Fellow: to improve the quality of care and the financing situation at the 217 peripheral health stations in Hai Phong Province.

1993 April - 1993 November: WHO in Malawi:

National Prescriber Training Officer, for the Malawi Essential Drugs Program.

1992 October - 1993 March: WHO, in the Republic of Yemen:

Consultant: To write a four year action plan for the National Essential Drug Program, and a pharmaceutical sector review.

1989 February - 1991 June: Britain Nepal Medical Trust, Nepal:

Medical Director, of this NGO, based in East Nepal with 120 Nepali and 8 expatriate staff. In eight hill districts the main programs were: TB services, Drug Schemes, Community Health Volunteers and Adult Women's Literacy.

1987 August - 1988 August: Health Unlimited in Burma:

Team Member To establish primary health care among a civilian population disrupted by war, along a river valley, in dense jungle, in the part of Burma run by the Karen.

**CONSULTANCIES**

September to December 1999. World Bank and Danish Trust fund project through Euro Health Group, in Indonesia,

Team Leader. To help formulate a master plan for health service delivery throughout North Sumatra, taking into account all interest groups and to include a unifying strategy to make use of the public and private sectors as well as non-government organizations. The characteristics of this master plan were that it should serve to protect the access for the poor. This involved two visits of six weeks and two weeks with a four-person team. It involved extensive consultation and consensus building at both National and Provincial level.

1999 April: Euro Health Group and the Danish Red Cross in Uganda

Deputy Team Leader. To make recommendations on the harmonization of the pharmaceutical supply system in Uganda. This was a four-week mission for a six member team including specialists in law, health economics, economics, pharmaceuticals, Ministry of Health activity and public health. The technical committee for the harmonization of drug supply in Uganda included senior people from the MOH, National Drug Administration, National Drug Stores and donors. Separate procurement by major donor programs, Government supply and the private sector had resulted in an inefficient and irrational situation from the health service provider’s point of view. The committee had asked for a group of consultants to assess the situation and make recommendations.

1999 March: Euro Health Group and the African Development Bank in Cote D’Ivoire

Consultant to assist in a study of the ADB staff medical benefits. This was mainly a health economics exercise to make a comparative assessment of the ADB benefits compared to other similar organizations and to work out cost effective ways of including pensioners and short term staff. Apart from assisting the health economist, my role was to assess both the quality and relevance of the ADB health center services and to assess other private services available in Abidjan.

1998 February: Euro Health Group and Danida in Vietnam

Consultant. Advising a team preparing a project with the Vietnam Health Insurance Company.

1997 March: Euro Health Group and the EU in Sierra Leone

Consultant. After the recent war in Sierra Leone the EU plan to donate around $ 30 million over five years to rehabilitate the health services. My role was to make a project proposal for the pharmaceutical sector

**SELECTED PUBLICATIONS**

Chalker J. Wagner A, Tomson G, Laing R, Johnson K, Wahlstrom R, and Ross-Degnan D, on behalf of INRUD-IAA. Appointment systems are essential for improving chronic disease care in resource-poor settings: learning from experiences with HIV patients in Africa **Int. Health** 2013**, 5 (3):** 163-165.

Chalker J, Andualem T, Gitau L, Ntaganira J, Obua C, Tadeg H, Waako P, Ross-Degnan D. Measuring adherence to antiretroviral treatment in resource-poor settings: The feasibility of collecting routine data for key indicators. **BMC Health Services Research** 2010 **10**:43

Ross-Degnan D, Pierre-Jacques M, Zhang F, Tadeg H, Gitau L, Ntaganira J, Balikuddembe R, Chalker J, Wagner A. Measuring adherence to antiretroviral treatment in resource-poor settings: The clinical validity of key indicators. **BMC Health Services Research** 2010 **10**:42

Chalker J. Wagner A, Tomson G, Laing R, Johnson K, Wahlstrom R, and Ross-Degnan D, on behalf of INRUD-IAA. Urgent need for coordination in adopting standardized antiretroviral adherence performance indicators. **Journal of Acquired Immune Deficiency Syndromes** 2010, **53(2):**159-161

Chalker J, Andualem T, Minzi O, Ntaganira J, Ojoo A, Waako P, Ross-Degnan D. Monitoring Adherence and Defaulting for Antiretroviral Therapy in 5 East African Countries: An Urgent Need for Standards; **Journal of the International Association of Physicians in AIDS Care, 7 (4):** 193-199, 2008

Mwatawala S, Sando D, Malele R, Moshiro C, Senyael B, Somi G, Maselle A, Chalker J, & Ross-Degnan D. Strengthening the appointment and tracking systems for patients on antiretroviral therapy in Tanzania: A optimizing adherence to ART as part of people-centered public health. **International Journal Of Person Centered Medicine***,*2012, **2(4):**825-836. doi:10.5750/ijpcm.v2i4.302.

Obua C, Gusdal A, Waako P, Chalker J, Tomson G, Wahlström R, and The INRUD-IAA Team. Multiple ART Programs Create a Dilemma for Providers to Monitor ARV Adherence in Uganda. **The Open AIDS Journal,** 2011**, 5**, 17-24.

Gitau L, Boruett P, Njogo S, Nguhiu P, Awour C, Kagai D, Chalker J, Tomson G, Wahlstrom R. Implementing tools to promote adherence to antiretroviral therapy at facilities in Kenya: Exploring staff’s perceptions, motivation and practice. **East African Medical Journal,** 2014**, 91 (10):** about to be published

Boruett P, Kagai D, Njogo S, Nguhiu P, Awuor C, Gitau L, Chalker J, Ross-Degnan D, Wahlström R, Tomson G, on behalf of INRUD-IAA. Facility-level intervention to improve attendance and adherence among patients on anti-retroviral treatment in Kenya¿a quasi-experimental study using time series analysis. **BMC Health Services Research** , 2013, **13:242**.

Gusdal AK, Obua C, Andualem T, Wahlström R, Chalker J, Fochsen G, on behalf of the INRUD-IAA project. Peer Counselor’s role in supporting patients’ adherence to ART in Ethiopia and Uganda. **AIDS Care**, June 2011 **23**:6, 657-662

Obua C, Gusdal A, Waako P, Chalker J, Tomson G, Wahlström R, and The INRUD-IAA Team. Multiple ART Programs Create a Dilemma for Providers to Monitor ARV Adherence in Uganda. **The Open AIDS Journal,** 2011**, 5**, 17-24.

Colvin CJ, Konopka S, Chalker JC, Jonas E, Albertini J, Amzel A, Fogg K. A Systematic Review of Health System Barriers and Enablers for Antiretroviral Therapy (ART) for HIV-Infected Pregnant and Postpartum Women. **PLOS One**, October 2014, 9(10) - e108150. doi: 10.1371/journal.pone.0108150.

Rowe A, Rowe S, Vujicic M, Ross-Degnan D, Chalker J, Holloway K, Peters D. **Chapter 3: Review of strategies to Improve Health Care provider Performance.** In Editors: Peters D, El Saharty S, Siadet B, Janovsky K, Vujicic M. **Improving Health Service Delivery in Developing Countries: From Evidence to Action**. World Bank 2009, Washington

Chalker J, Ratanawijitrasin S, Chuc NTK, Petzold M and Tomson G. Effectiveness of a multi-component intervention on dispensing practices at private pharmacies in Vietnam and Thailand - A randomized controlled trial. **Social Science and Medicine,** **60 (1):** 131-141, 2005

Chalker J, Chuc NTK, Falkenberg T, Do NT and Tomson G. Private pharmacies in Hanoi Vietnam: A randomised trial of a 2 year multi-component intervention on knowledge and stated practice regarding ARI, STD and antibiotic/steroid requests, **Journal of Tropical Medicine and International Health**, **7(9)**:803-81. September 2002

Chalker J. Improving antibiotic prescribing in Hai Phong Province, Vietnam. The “Antibiotic Dose” indicator, **Bulletin of the World Health Organisation, 79 (4):** 313-320, 2001

Chalker J, Chuc NTK, Falkenberg T, Do NT and Tomson G. STD management by Private Pharmacies in Hanoi:Practice and knowledge of drug sellers. **Sexually Transmitted Infections**. **76(4)**: 299-302. August 2000

Chalker J: Effect of a drug supply and cost sharing system on prescribing patterns and on utilisation of health facilities, a controlled trial from health posts in the hills of Nepal. **Health Policy and Planning 10 No 4:** pp 423-430**,** 1995.

Chalker J: Viet Nam: profit and loss in health care. **World Health Forum** Vol 16 **No 2**: 194-5, 1995.

Chalker J, Madhu Kapali and Bhagwati Khadka: Health post usage in a mountain district in eastern Nepal. **Essential Drugs Monitor, No 14,** 1993.

Chalker J; Nepal: Experiments in cost sharing; **The Lancet**, **337**: 1336-7, 1991.

**MEMBERSHIPS**

General Medical Council, UK: Full registration as a medical practitioner

Fellow of the Faculty of Public Health Medicine of the Royal College of Physicians of the United Kingdom.

## 10.8 Dr. Dennis Ross-Degnan

Dennis Ross-Degnan, ScD

Brief Curriculum Vitae

**Key Qualifications**

Dr. Dennis Ross-Degnan is Associate Professor at the Department of Population Medicine at Harvard Medical School and Director of Research at Harvard Pilgrim Health Care Institute. He holds a doctorate in health policy and management from the Harvard School of Public Health. For over thirty years, Dr. Ross-Degnan’s work has focused on improving health systems in the US and low income countries, including research on the behavioral and systems factors that determine use of medicines, impacts of health and pharmaceutical policies on utilization and clinical outcomes, and appropriate methods for health and pharmaceutical systems research. He has served as lead or senior investigator on many domestic and international research studies to evaluate health systems initiatives to improve quality and outcomes of care, including: different forms of interactive education; innovations in insurance benefits design; electronic outreach to health providers and patients to improve utilization; and systems interventions in low resource settings to promote medication adherence. In 1990, Dr. Ross-Degnan co-founded the International Network for Rational Use of Drugs (INRUD), a global network of academics, health managers, and policymakers involved in testing interdisciplinary interventions to improve use of medicines. He has consulted extensively with the World Health Organization on access to and appropriate use of medicines, and on evaluation of WHO health systems intervention strategies. From 2004 to 2013, he co-directed the WHO Collaborating Center on Pharmaceutical Policy based jointly at Harvard Medical School and Boston University. He has received the A. Clifford Barger Excellence in Mentoring Award from Harvard Medical School and the Klaus Peter International Teaching Award for distinction in international medical education and mentoring from Harvard Medical International and Harvard Medical School.

**Education**

Doctor of Science, Department of Health Policy and Management, Harvard School of Public Health, Boston, MA, 1987

Master of Science in Public Health, Department of Health Policy and Management, Harvard School of Public Health, Boston, MA, 1979

Bachelor of Arts, Department of Psychology, Boston College, Chestnut Hill, MA, 1972

**Professional Experience**

**Associate Professor (1999-present), Assistant Professor (1992-1999), Instructor (1987-1992), Consultant (1982-1986), Department of Population Medicine (2009-present), Ambulatory Care and Prevention (1993-2009) and Social Medicine (1982-1993), Harvard Medical School and Harvard Pilgrim Health Care Institute, Boston, MA, 1982–present**

- Conducts U.S. and international health systems research and training; technical assistance on clinical and economic impacts of pharmaceutical policies; pharmaceutical measurement methodologies; interventions to improve medication use; technical support and research capacity building; program evaluation

**Visiting Fellow, Discipline in Clinical Pharmacology, Faculty of Medicine, University of Newcastle, Waratah, New South Wales, Australia and Inter University Center - Social Studies, Gadjah Mada University, Yogyakarta, Indonesia, 1993-1995**

- Joint Harvard, Newcastle, Gadjah Mada University project to develop training programs and intervention projects in Southeast Asia region related to improving pharmaceutical use

**Project Director, King Khaled Eye Specialist Hospital, Riyadh, Saudi Arabia for International Eye Foundation, Bethesda, MD, 1983-1985**

- Design, manage, and analyze national clinical examination survey of eye disease and national institutional survey of eye care services

**Health Planner, Kenya Rural Blindness Prevention Project, Nairobi, Kenya for International Eye Foundation, Bethesda, MD), 1980-1981**

- Develop community based blindness prevention projects; implement and analyze field prevalence surveys of eye disease; design monitoring and evaluation plan

**Research Fellow, Dept. of COMMUNITY Health, University of Nairobi Medical School, Nairobi, Kenya (Sinclair Kennedy Traveling Fellow), 1979-1980**

- Conduct research on coordinated nutrition planning and supplementary feeding program for children; teach sections in statistics and research methods

**Short-term Consulting (last 10 years)**

**Department of Medicines Policy and Standards (formerly Essential Drugs and Medicines Policy Department), World Health Organization, 1998-present**

- Develop strategy for evaluating global, regional, country action programs and technical products; assist Chinese State Drug Authority and Ministry of Health in design and implementation of national prescribing quality improvement interventions; develop methodology for country program monitoring indicators; develop household survey on access to medicines; develop methodologies for measuring medicines prices in low resource settings

**Institute for Healthcare Improvement (IHI) JOINTS Project, 2011**

- Assist in developing approaches and materials for large-scale intervention testing methods for disseminating best practice recommendations in hip and knee replacement surgery

**United Kingdom Department for International Development, 2007-2009**

- Assist in the development of the strategy and assessment framework for the global Medicines Transparency Alliance and in the implementation of MeTA-Philippines

**World Health Organization Western Pacific Regional Office, 1996-2010.**

- Expert Committee to develop regional essential medicines action plan (2009-2010); with Philippines Department of Health to develop strategies for improving practice in private sector pharmacies (1996-1997)

**Center for Pharmaceutical Management (formerly Drug Management Program), Management Sciences for Health, Boston, MA, 1986-2010**

- Data systems and quantitative indicators for assessing pharmaceutical needs and drug utilization; evaluate pharmaceuticals for child survival in Indonesia, Nepal, and Honduras; evaluate health sector financing projects in Indonesia and Kenya; design of intervention to improve antiretroviral therapy in Namibia

**Languages**

English – native

**Papers/Publications (selected from over 200 peer-reviewed publications)**

Wagner AK, Graves AJ, Fan Z, Walker S, Zhang F, Ross-Degnan D. Need for and access to health care and medicines: are there gender inequities? PLoS ONE. 2013;8(3):e57228. Epub 2013 Mar 7.

Stephens P, Ross-Degnan D, Wagner AK. Does access to medicines differ by gender? Evidence from 15 low and middle income countries. Health Policy. 2013 Apr;110(1):60-6. Epub 2013 Feb 17.

Wharam JF, Soumerai S, Trinacty C, Eggleston E, Zhang F, LeCates R, Canning C, Ross-Degnan D. Impact of emerging health insurance arrangements on diabetes outcomes and disparities: rationale and study design. Prev Chronic Dis. 2013;10E11.

Garabedian LF, Ross-Degnan D, Ratanawijitrasin S, Stephens P, Wagner AK. Impact of universal health insurance coverage in Thailand on sales and market share of medicines for non-communicable diseases: an interrupted time series study. BMJ Open. 2012;2(6).

Pérez-Cuevas R, Doubova SV, Suarez-Ortega M, Law M, Pande AH, Escobedo J, Espinosa-Larrañaga F, Ross-Degnan D, Wagner AK. Evaluating quality of care for patients with type 2 diabetes using electronic health record information in Mexico. BMC Med Inform Decis Mak. 2012 Jun 6. [Epub ahead of print].

Wharam JF, Graves AJ, Zhang F, Soumerai SB, Ross-Degnan D, Landon BE. Two-year trends in cancer screening among low socioeconomic status women in an HMO-based high-deductible health plan. J Gen Intern Med. 2012 Sep;27(9):1112-9. Epub 2012 Apr 29.

Zhang F, Wagner AK, Ross-Degnan D. Simulation-based power calculation for designing interrupted time series analyses of health policy interventions. J Clin Epidemiol. 2011 Nov;64(11):1252-61.

Pande AH, Ross-Degnan D, Zaslavsky AM, Salomon JA. Effects of healthcare reforms on coverage, access, and disparities: quasi-experimental analysis of evidence from Massachusetts. Am J Prev Med. 2011 Jul;41(1):1-8.

Reiss SK, Ross-Degnan D, Zhang F, Soumerai SB, Zaslavsky AM, Wharam JF. Effect of switching to a high-deductible health plan on use of chronic medications. Health Serv Res. 2011 Oct;46(5):1382-401. Epub 2011 Mar 17.

Galbraith AA, Ross-Degnan D, Soumerai SB, Rosenthal MB, Gay C, Lieu TA. Nearly half of families in high-deductible health plans whose members have chronic conditions face substantial financial burden. Health Aff (Millwood). 2011 Feb;30(2):322-31.

Serumaga B, Ross-Degnan D, Avery AJ, Elliott RA, Majumdar SR, Zhang F, Soumerai SB. Effect of pay for performance on the management and outcomes of hypertension in the United Kingdom: interrupted time series study. BMJ. 2011 Jan 25. [Epub ahead of print].

Faden L, Vialle-Valentin C, Ross-Degnan D, Wagner A. Active pharmaceutical management strategies of health insurance systems to improve cost-effective use of medicines in low- and middle-income countries: a systematic review of current evidence. Health Policy. 2011 May;100(2-3):134-43. Epub 2010 Dec 24.

Carapinha JL, Ross-Degnan D, Desta AT, Wagner AK. Health insurance systems in five Sub-Saharan African countries: medicine benefits and data for decision making. Health Policy. 2011 Mar;99(3):193-202. Epub 2010 Dec 16.

Zhang F, Wharam JF, Ross-Degnan D. Comparing simulation and threshold approaches when analysing data with probabilities of categories. J Eval Clin Pract. 2010 Oct;16(5):964-7.

Wagner AK, Graves AJ, Reiss SK, Lecates R, Zhang F, Ross-Degnan D. Access to care and medicines, burden of health care expenditures, and risk protection: results from the World Health Survey. Health Policy. 2011 May;100(2-3):151-8. Epub 2010 Sep 9.

Bertoldi AD, Barros AJ, Camargo AL, Hallal PC, Vandoros S, Wagner A, Ross-Degnan D. Household expenditures for medicines and the role of free medicines in the Brazilian public health system. Am J Public Health. 2011 May;101(5):916-21. Epub 2010 Aug 19.

Chen W, Tang S, Sun J, Ross-Degnan D, Wagner AK. Availability and use of essential medicines in China: manufacturing, supply, and prescribing in Shandong and Gansu provinces. BMC Health Serv Res. 2010 Jul 17. [Epub ahead of print].

Briesacher BA, Ross-Degnan D, Wagner AK, Fouayzi H, Zhang F, Gurwitz JH, Soumerai SB. Out-of-pocket burden of health care spending and the adequacy of the Medicare Part D low-income subsidy. Med Care. 2010 Jun;48(6):503-9.

Madden JM, Meza E, Ewen M, Laing RO, Stephens P, Ross-Degnan D. Measuring medicine prices in Peru: validation of key aspects of WHO/HAI survey methodology. Rev Panam Salud Publica. 2010 Apr;27(4):291-9.

Chalker JC, Andualem T, Gitau LN, Ntaganira J, Obua C, Tadeg H, Waako P, Ross-Degnan D, . Measuring adherence to antiretroviral treatment in resource-poor settings: the feasibility of collecting routine data for key indicators. BMC Health Serv Res. 2010 Feb 19.

Ross-Degnan D, Pierre-Jacques M, Zhang F, Tadeg H, Gitau L, Ntaganira J, Balikuddembe R, Chalker J, Wagner AK. Measuring adherence to antiretroviral treatment in resource-poor settings: the clinical validity of key indicators. BMC Health Serv Res. 2010 Feb 19.

**Professional Affiliations**

**Member,** American Public Health Association, 1978-present

**Member,** AcademyHealth, 1987-present

**References**

| **Name** | **Position** | **Telephone** | **E-mail** |
| --- | --- | --- | --- |
| John C. Chalker | Center for Pharmaceutical Management, Management Sciences for Health | (703) 524-6575 | jchalker@msh.org |
| Richard Laing | Professor, International Health, Boston University School of Public Health | (617) 414-1445 | richardl@bu.edu |
| Kathleen Holloway | Regional Advisor Essential Medicines, WHO Regional Office for South-East Asia | +91 11 2337 0804 | hollowayk@who.int |

## 10.9 Dr. Catherine Vialle-Valentin

| **Date Prepared:** | **5 February 2015** |
| --- | --- |
| **Name:** | **Catherine Vialle-Valentin** |
| **Office Address:** | **Department of Population Medicine, 133 Brookline Avenue, Boston, MA 02215** |
| **Work Phone:** | **+1 617 509 9312** |
| **Work Email:** | [**Catherine.Vialle@post.harvard.edu**](mailto:Catherine.Vialle@post.harvard.edu) |
| **Work Fax:** | **+1 617 859 8112** |
| **Place of Birth:** | **Saint-Julien-en-Jarez, France** |

[Education](http://cv.hms.harvard.edu/index.php?page=education)

| 1982 | MD | Medicine | Claude Bernard University, Lyon, France |
| --- | --- | --- | --- |
| 2004 | SM | Health Care Management | Harvard School of Public Health, Boston, MA |

[Postdoctoral Training](http://cv.hms.harvard.edu/index.php?page=postdoc)

| Year(s) | Title | Specialty/Discipline  (Lab PI for postdoctoral research) | Institution |
| --- | --- | --- | --- |

| 10/1985-04/1987 | Research Fellow | Lipid Metabolism | University of Texas Health Science Center, San Antonio, TX |
| --- | --- | --- | --- |
| 2006-2007 | Research Fellow | Pharmaceutical Policy Research | Department of Ambulatory Care and Prevention, Harvard Medical School, Boston, MA |
| 2007-2008 | Thomas O. Pyle Fellow | Pharmaceutical Policy Research | Harvard Pilgrim Health Care Foundation and Harvard Medical School, Boston, USA |

[Faculty Academic Appointments](http://cv.hms.harvard.edu/index.php?page=academic_appt)

| Year(s) | Academic Title | Department | Academic Institution |
| --- | --- | --- | --- |

| 1982-1985 | Assistante des Hopitaux | Endocrinology | Claude Bernard Medical School, Lyon, France |
| --- | --- | --- | --- |
| 2008-2010 | Research Associate | Population Medicine | Harvard Medical School and Harvard Pilgrim Health Care Institute, Boston, USA |
| 2010- | Senior Researcher | Population Medicine | Harvard Medical School and Harvard Pilgrim Health Care Institute, Boston, USA |

[Appointments at Hospitals/Affiliated Institutions](http://cv.hms.harvard.edu/index.php?page=hospital_appt)

| Year(s) | Position Title | Department (Division, if applicable) | Institution |
| --- | --- | --- | --- |

| 10/1982-09/1985 | Chef de Clinique Endocrinologue | Endocrinology | Hôpital de l'Antiquaille, CHU Lyon, France |
| --- | --- | --- | --- |
| 05/1987-09/1987 | Attending Endocrinologist | Endocrinology | Hôpital de l'Antiquaille, CHU Lyon, France |

[Other Professional Positions](http://cv.hms.harvard.edu/index.php?page=other)

| Year(s) | Position Title | Institution |
| --- | --- | --- |

| 1988-1990 | Medical Advisor | Procter & Gamble Pharmaceuticals, Norwich, NY |  |
| --- | --- | --- | --- |
| 1990-1993 | Senior Medical Advisor | Procter & Gamble Pharmaceuticals, London, UK | |
| 1993-1994 | Medical Consultant | Procter & Gamble Pharmaceuticals, Cincinnati, OH | |
| 1995 | Associate Medical Director | PAREXEL International Corporation, Boston, MA | |
| 1995-1998 | Medical Director | PAREXEL International Corporation, Boston, MA | |
| 1998-2006 | Senior Medical Director | PAREXEL International Corporation, Boston, MA | |
| 2008- | Epidemiologist | Harvard Pilgrim Health Care Institute, Boston, MA | |

[Professional Societies](http://cv.hms.harvard.edu/index.php?page=societies)

| Year(s) of  Membership | Society Name |  |
| --- | --- | --- |
| Dates of Role(s) | Title of Role(s) |  |

| 1982- | Ordre des Médecins, France | Member |
| --- | --- | --- |
| 1989-2003 | Drug Information Association | Member |
| 1990-1995 | British Association for Pharmaceutical Physicians | Member |
| 1996-2001 | American Diabetes Association | Member |

[Publications](http://cv.hms.harvard.edu/index.php?page=publications)

[Peer-reviewed publications in print or other media](http://cv.hms.harvard.edu/index.php?page=peer_review)

*Research Investigations*

| 1. | Chauplannaz G, Sassolas A, El Haouchi S, Serusclat P, **Valentin C**, Trillet M, Schott B. Intérêt du dosage de l'apolipoprotéine B chez des sujets ayant eu un infarctus cérébral. *Lyon Medical*. 1980;246(20):447-450. |
| --- | --- |
| 2. | **Valentin C**, Chauplannaz G, Duquesnel J, Sindou M, Trocme Y, Trillet M. Anévrysme géant juxta-clinoidien à séméiologie akinéto-hypertonique et endocrinienne. *Lyon Médical*. 1981;246(14):77-80. |
| 3. | Verdugo C, Perrot L, Ponsin G, **Valentin C**, Berthezène F. Time-course of alterations of high density lipoproteins (HDL) during thyroxine administration to hypothyroid women. *Eur J Clin Invest*. 1987;17(4):313-6. PMID 3117568 |
| 4. | **Valentin C**, Chauplannaz G. La médiation chimique dans les systèmes nerveux de commande et de transmission. *Cahiers Médicaux*. 1981;6(29):1901-1909. |
| 5. | Berthezène F, Perrot L, De Parscau L, **Valentin C**, Richard L. [Thyroid hormones and the metabolism of lipoproteins]. *Ann Endocrinol* (Paris). 1983;44(1):73-6. PMID 6347029 |
| 6. | Berthezène F, Perrot L, **Valentin C**, Sautot G. [Insulin-dependent diabetes and lipoprotein metabolism]. *Journ Annu Diabetol Hotel Dieu*. 1984: pp. 115-23. PMID 6399527 |
| 7. | Berthezène F, **Valentin C**. Hyperlipidémies. *La Pratique Médicale*. 1985;79:7-10. |
| 8. | Valente AJ, Graves DT, **Vialle-Valentin CE**, Delgado R, Schwartz CJ. Purification of a monocyte chemotactic factor secreted by nonhuman primate vascular cells in culture. *Biochemistry*. 1988;27(11):4162-8. PMID3415979 |
| 9. | Ponsin G, **Vialle-Valentin C**, Berthezène F. Alterations of high density lipoproteins induced by thyroid hormones in man and rat. *Adv Exp Med Biol*. 1991;285:147-54. PMID 1858545 |
| 10. | **Vialle-Valentin CE**, Ross-Degnan D, Ntaganira J and Wagner AK. Medicines Coverage and Community-Based Health Insurance in Low-Income Countries. *Health Research Policy and Systems*. 2008;(6):11. PMC2584623 |
| 11. | Faden L, **Vialle-Valentin C**, Ross-Degnan D, Wagner AK. Active pharmaceutical management strategies of health insurance systems to improve cost-effective use of medicines in low- and middle-income countries: A systematic review of current evidence. *Health Policy*. 2011; 100: 134-143. PMOID 21185616 |
| 12. | **Vialle-Valentin CE**, LeCates RF, Zhang F, Desta AT, Ross-Degnan D. Predictors of antibiotic use in African communities: evidence from medicines household surveys in five countries. *Tropical Medicine and International Health*. 2012; 17: 211–222. PMID 21999394 |
| 13 | Ewen M, Al Sakit M, Saadeh R, **Vialle-Valentin CE**, Seita A. UN Relief and Works Agency for Palestine Refugees in the Near East's medicine procurement processes and prices: a comparative performance assessment. Health in the Occupied Palestinian Territory 2012. *The Lancet*, 1 October 2012, 380: S15. doi:10.1016/S0140-6736(13)60197-X. |
| 14 | Holloway KA, Ivanovska V, Wagner AK, **Vialle-Valentin C**, Ross-Degnan D. Have we improved use of medicines in developing and transitional countries and do we know how to? Two decades of evidence. *Tropical Medicine & International Healt*h, 2013 Jun;18(6):656-64. PMID 23648177 |
| 15 | Holloway KA, Ivanovska V, Wagner AK, **Vialle-Valentin C**, Ross-Degnan D. Prescribing for acute childhood infections in developing and transitional countries 1990-2009. *Paediatr Int Child Health*. 2014 Feb 6: 2046905514Y0000000115. [Epub ahead of print]. PMID 24621245 |
| 16 | Emmerick IC, Luiza VL, Camacho LAB, **Vialle-Valentin CE**, Ross-Degnan D. What are the main barriers in household access to medicines for chronic conditions in Latin America? A health system perspective (Accepted for publication in Health Policy and Planning pending revisions) |
| 17 | Pande AH, **Vialle-Valentin CE**, LeCates R, Ross-Degnan D, Wagner AK. Are there gender disparities in access to medicines in middle and low income countries? Evidence from WHO household medicines surveys in five Sub Saharan African countries. (Submitted) |
| 18 | **Vialle-Valentin CE**, LeCates RF, Zhang F, Ross-Degnan D. Treatment of Febrile Illness with Artemisinin Combination Therapy: Prevalence and Predictors in Five African Household Surveys. (Accepted for publication in the J Pharm Policy Pract.) |
| 19 | **Vialle-Valentin CE**, Serumaga B, Wagner AK, Ross-Degnan D. Evidence on Access to Medicines for Chronic Diseases from Household Surveys in Five Low- and Middle-Income Countries. Health Policy Plan. 2014 Sep 24 |
| 20 | Ewen M, Al Sakit M, Saadeh R, **Vialle-Valentin CE**, Seita A, Bunders B and Laing R. Comparative assessment of medicine procurement prices in the United Nations Relief and Works Agency for Palestine Refugees in the Near East (UNRWA) J Pharm Policy Pract. 2014 Oct 21;7 |
| 21 | Balaban RB, Galbraith AA, Burns ME, **Vialle-Valentin CE**, Larochelle MR, and Ross-Degnan D Randomized Controlled Trial to Test a Patient Navigator Intervention to Improve Post-Discharge Care Transitions in a High Risk Safety Net Population J Gen Intern Med. 2015 Jan 24. |

*Other Peer-Reviewed Publications*

| 1. | Faden L, **Vialle-Valentin C**, Ross-Degnan D, Wagner AK. The Role of Health Insurance in the Cost-Effective Use of Medicines in Low- and Middle-Income Countries. Commissioned paper. WHO/Health Action International, Amsterdam. |
| --- | --- |

[Non-peer reviewed scientific or medical publications/materials in print or other media](http://cv.hms.harvard.edu/index.php?page=peer_review)

| 1. | WHO, 2009. Medicines Use in Primary Care in Developing and Transitional Countries. A Fact Book summarizing results from studies reported between 1990 and 2006.  Contribution to the report preparation. |
| --- | --- |
| 2 | Pharmaceutical Situation in Barbados. WHO Assessment of Level II – Health Facilities and Household Survey. (ISBN 978-92-75-13158-9) Washington; Pan-American Health Organization. 2011.  Analysis and report preparation |
| 3 | USAID/MSH (In press) Improving Access and Use of Medicines for Child Health- A Guide to Developing Interventions  Contribution to the guide revision |

[Thesis](http://cv.hms.harvard.edu/index.php?page=thesis)

| Vialle-Valentin C. Activité lipasique post-héparinique sérique. Mise au point d’une méthode de dosage. Intérêt au cours des dysthyroïdies chez l’homme. Lyon I - France: Université Claude-Bernard;1982. |
| --- |

1. Ross-Degnan D, Pierre-Jacques M, Zhang F, et al. Measuring adherence to antiretroviral treatment in resource-poor settings: the clinical validity of key indicators. *BMC Health Serv Res* 2010;10:42. [↑](#footnote-ref-1)
2. Chalker J, Andualem T, Minzi O, et al. Monitoring adherence and defaulting for antiretroviral therapy in 5 East African countries: an urgent need for standards. *J Int Assoc Physicians AIDS Care* 2008;7:193–9. [↑](#footnote-ref-2)
3. Mwatawala S, Sando D, Malele R, et al. Strengthening the appointment and tracking systems for patients on antiretroviral therapy in Tanzania: a strategy to optimize adherence to ART. Int J Pers Cent Med 2012;2(4):825-36. [↑](#footnote-ref-3)
4. Nyamusore J, Hinda R, Mutabazi V, et al. Effects of patient tracking systems and providers incentives on patient appointment keeping in Rwanda. Presentation at the Third International Conference for Improving Use of Medicine, Antalya, Turkey, November 2011. <http://www.inrud.org/ICIUM/ConferenceMaterials/817-nyamusore-_c.pdf#search=%22nyamusore%22>. [↑](#footnote-ref-4)
5. Boruett P, Kagai D, Njogo S, Nguhiu P, Awuor C, Gitau L, Chalker J, Ross-Degnan D, Wahlström R, Tomson G, on behalf of INRUD-IAA. Facility-level intervention to improve attendance and adherence among patients on anti-retroviral treatment in Kenya: a quasi-experimental study using time series analysis. BMC Health Services Research , 2013, 13:242. [↑](#footnote-ref-5)
6. Gusdal AK, Obua C, Andualem T, Wahlström R, Tomson G, Peterson S, Ekström AM, Thorson A, Chalker J, Fochsen G, on behalf of the INRUD-IAA project. Voices on adherence to ART in Ethiopia and Uganda: A matter of choice or simply not an option? *AIDS Care* 2009; [21](http://www.informaworld.com.ezp-prod1.hul.harvard.edu/smpp/title~db=all~content=t713403300~tab=issueslist~branches=21#v21) ([11](http://www.informaworld.com.ezp-prod1.hul.harvard.edu/smpp/title~db=all~content=g916270514)):1381–1387. [↑](#footnote-ref-6)
7. Ibid. [↑](#footnote-ref-7)
8. Ross-Degnan D, Pierre-Jacques M, Zhang F, et al. Measuring adherence to antiretroviral treatment in resource-poor settings: the clinical validity of key indicators. *BMC Health Serv Res* 2010;10:42. [↑](#footnote-ref-8)
9. Chalker J, Andualem T, Gitau L et al. Measuring adherence to antiretroviral treatment in resource poor settings using available data: a health system approach. *BMC Health Serv Res* 2010;10:43. [↑](#footnote-ref-9)
10. Chalker J, Andualem T, Minzi O et al. Monitoring adherence and defaulting for antiretroviral therapy in 5 East African countries: an urgent need for standards. *J Int Assoc Physicians AIDS Care* 2008;7:193–9. [↑](#footnote-ref-10)
11. Mwatawala S, Sando D, Malele R, et al. Strengthening the appointment and tracking systems for patients on antiretroviral therapy in Tanzania: a strategy to optimize adherence to ART. *Int J Pers Cent Med.* 2012;2(4):825-36. [↑](#footnote-ref-11)
12. Nyamusore J, Hinda R, Mutabazi V, et al. Effects of patient tracking systems and providers incentives on patient appointment keeping in Rwanda. Presentation at the Third International Conference for Improving Use of Medicine, Antalya, Turkey, November 2011. <http://www.inrud.org/ICIUM/ConferenceMaterials/817-nyamusore-_c.pdf#search=%22nyamusore%22>. [↑](#footnote-ref-12)
13. Boruett P, Kagai D, Njogo S, Nguhiu P, Awuor C, Gitau L, Chalker J, Ross-Degnan D, Wahlström R, Tomson G, on behalf of INRUD-IAA. Facility-level intervention to improve attendance and adherence among patients on anti-retroviral treatment in Kenya: a quasi-experimental study using time series analysis. *BMC Health Serv Res* 2013, 13:242. [↑](#footnote-ref-13)
14. Bangsberg DR, Perry S, Charlebois ED, Clark RA, Roberston M, Zolopa AR, et al. Nonadherence to highly active antiretroviral therapy predicts progression to AIDS AIDS, 2001;15(9): 1181-1183. [↑](#footnote-ref-14)
15. Garcia de Olalla P, Knobel H, Carmona A, Guelar A, Lopez-Colomes JL, Cayla JA. Impact of adherence and highly active antiretroviral therapy on survival in HIV-infected patients. *JAIDS* 2002; 30(1), 105-110. [↑](#footnote-ref-15)
16. Bangsberg DR, Kroetz DL, Deeks SG. Adherence-resistance relationships to combination HIV antiretroviral therapy. *Current HIV/AIDS Reports* 2007; 4(2):65-72. [↑](#footnote-ref-16)
17. Diabate S, Alary M, Koffi CK. Determinants of adherence to highly active antiretroviral therapy among HIV-1-infected patients in Cote d'Ivoire. *AIDS* 2007; 21(13):1799-1803. [↑](#footnote-ref-17)
18. Sarna A, Pujari S, Sengar AK, Garg R, Gupta I, Dam J. Adherence to antiretroviral therapy & its determinants amongst HIV patients in India. *Indian J Med Res* 2008;127(1):28-36. [↑](#footnote-ref-18)
19. Gilks CF, Crowley S, Ekpini R, Gove S, Perriens J, Souteyrand Y, et al. The WHO public-health approach to antiretroviral treatment against HIV in resource-limited settings. *Lancet* 2006;368(9534):505-510. [↑](#footnote-ref-19)
20. Schneider H, Blaauw D, Gilson L, Chabikuli N, Goudge J. Health systems and access to antiretroviral drugs for HIV in Southern Africa: service delivery and human resources challenges. *Reprod Health Matters* 2006;14(27):12-23. [↑](#footnote-ref-20)
21. Uzochukwu BS, Onwujekwe OE, Onoka AC, Okoli C, Uguru NP, Chukwuogo OI. Determinants of non-adherence to subsidized anti-retroviral treatment in southeast Nigeria. *Health Policy Plan* 2009; 24(3):189-196. [↑](#footnote-ref-21)
22. Chalker JC, Andualem T, Gitau LN, Ntaganira J, Obua C, Tadeg H, Waako P, Ross-Degnan D; INRUD-IAA. Measuring adherence to antiretroviral treatment in resource-poor settings: the feasibility of collecting routine data for key indicators. *BMC Health Serv Res* 2010 Feb 19;10:43. doi: 10.1186/1472-6963-10-43. [↑](#footnote-ref-22)
23. Ross-Degnan D, Pierre-Jacques M, Zhang F, Tadeg H, Gitau L, Ntaganira J, Balikuddembe R, Chalker J, Wagner AK; INRUD IAA. Measuring adherence to antiretroviral treatment in resource-poor settings: the clinical validity of key indicators. *BMC Health Serv Res* 2010 Feb 19;10:42. doi: 10.1186/1472-6963-10-42. [↑](#footnote-ref-23)
24. Gusdal AK, Obua C, Andualem T, Wahlström R, Chalker J, Fochsen G, on behalf of the INRUD-IAA project. Peer counselor’s role in supporting patients’ adherence to ART in Ethiopia and Uganda. *AIDS*

    *Care* June 2011 23:6, 657-662. [↑](#footnote-ref-24)
25. Gusdal AK, Obua C, Andualem T, Wahlstrom R, Tomson G, Peterson S, Ekstrom AM, Thorson A, Chalker J, Fochsen G; INRUD-IAA project.Voices on adherence to ART in Ethiopia and Uganda: a matter of choice or simply not an option? *AIDS Care* 2009 Nov;21(11):1381-7. [↑](#footnote-ref-25)
26. Green, L.W. and Kreuter, M.W. Health Program Planning: An Educational and Ecological Approach. 4th edition. NY: McGraw-Hill Higher Education, 2005. [↑](#footnote-ref-26)
27. Ross-Degnan D, Pierre-Jacques M, Zhang F, et al. Measuring adherence to antiretroviral treatment in resource-poor settings: the clinical validity of key indicators. *BMC Health Serv Res* 2010;10:42. [↑](#footnote-ref-27)
28. Mwatawala S, Sando D, Malele RS, Moshiro C, Senyael BR, Somi G, Maselle A, Chalker J, Ross-Degnan D. Strengthening the appointment and tracking systems for patients on antiretroviral therapy in Tanzania: optimizing adherence to ART as part of people-centered public health. Intl Journal of Person Centered Medicine 2012; 2(4): 825-836. [↑](#footnote-ref-28)
29. Boruett P, Kagai D, Njogo S, Nguhiu P, Awuor C, Gitau L, Chalker J, Ross-Degnan D, Wahlström R, Tomson G, INRUD –IAA. Facility-level intervention to improve attendance and adherence among patients on anti-retroviral treatment in Kenya: A quasi-experimental study using time series analysis. BMC Health Services Research.2013, 13:242. [↑](#footnote-ref-29)
30. Obua C, Kayiwa J, Waako P, Tomson G, Balidawa H, Chalker J, Ross-Degnan D, Wahlstrom R. Improving adherence to antiretroviral treatment in Uganda with a low resource facility-based intervention. Global Health Action. 2014; 7:24198. [↑](#footnote-ref-30)
31. Chalker J, Wagner AK, Tomson G, Johnson K, Wahlström R, Ross-Degnan D. Appointment systems are essential for improving chronic disease care in resource-poor settings: Learning from experiences with HIV patients in Africa. International Health 2013 (Jul 12): 1-3. doi:10.1093/inthealth/iht013 [↑](#footnote-ref-31)
32. Chalker JC, Andualem T, Gitau LN, Ntaganira J, Obua C, Tadeg H, Waako P, Ross-Degnan D, INRUD-IAA. Measuring adherence to antiretroviral treatment in resource-poor settings: the feasibility of collecting routine data for key indicators. BMC Health Serv Res. 2010 Feb 19; 10: 43. [↑](#footnote-ref-32)
33. Ross-Degnan D, Pierre-Jacques M, Zhang F, Tadeg H, Gitau L, Ntaganira J, Balikuddembe R, Chalker J, Wagner AK, INRUD IAA. Measuring adherence to antiretroviral treatment in resource-poor settings: the clinical validity of key indicators. BMC Health Serv Res. 2010 Feb 19; 10:42. [↑](#footnote-ref-33)
34. Wagner AK, Soumerai SB, Zhang F, Ross-Degnan D. Segmented regression analysis of interrupted time series studies in medication use research. J Clin Pharm Ther. 2002 Aug; 27 (4):299-309. PMID: 12174032. [↑](#footnote-ref-34)
35. Zhang F, Wagner AK, Soumerai SB, Ross-Degnan D. Methods for estimating confidence intervals in interrupted time series analyses of health interventions. J Clin Epidemiol. 2009 Feb; 62 (2):143-8. PMID: 19010644. [↑](#footnote-ref-35)
36. Kong M, Cambon A, Smith MJ. Extended Logistic Regression Model for Studies with Interrupted Events, Seasonal Trend, and Serial Correlation. Communication in Statistics – Theory and Methods. 2012; 31: 3528-3543. [↑](#footnote-ref-36)
37. Hosmer DW, Lemeshow S. Chapter 7. Extensions of the Proportional Hazards Model. Applied Survival Analysis. John Wiley & Sons, NY: 1999. [↑](#footnote-ref-37)
38. Wagner A, Ross-Degnan D, Gurwitz J, Zhang F, Gilden D, Soumerai S. Effect of New York state regulatory action on benzodiazepine prescribing and hip fracture rates. Annals of Internal Medicine 2007;146:96 - 103. [↑](#footnote-ref-38)
39. Shadish, Cook TD, Cambell DT. Experimental and Quasi-Experimental Designs for Generalized Causal Inference. Boston; Houghton Mifflin.: 2002. [↑](#footnote-ref-39)
40. Rossi PH, Lipsey MW, Freeman HE. Evaluation: A Systematic Approach (7th ed.). Los Angeles; SAGE Publications: 2004. [↑](#footnote-ref-40)
41. Fretheim A, Soumerai SB, Zhang F, Oxman AD, Ross-Degnan D. Interrupted time series analysis yielded an effect estimate concordant with the cluster-randomized controlled trial result. Journal of Clinical Epidemiology 2013 Aug;66(8):883-7. [↑](#footnote-ref-41)
42. Fretheim A, Zhang F, Ross-Degnan D, Oxman AD, Cheyne H, Foy R, Goodacre S, Herrin J, Kerse N, McKinlay RJ, Wright A, Soumerai SB. Cluster Randomized Trials versus Interrupted Time-Series: A comparative study of methods for evaluating health system interventions. (in revision) [↑](#footnote-ref-42)
43. Wagner AK, Soumerai SB, Zhang F, Ross-Degnan D. Segmented regression analysis of interrupted time series studies in medication use research. J Clin Pharm Ther. 2002 Aug; 27 (4):299-309. PMID: 12174032. [↑](#footnote-ref-43)
44. Cook TD, Campbell, DT. Quasi-Experimentation: Design and Analysis Issues for Field Settings. Boston: Houghton Mifflin; 1979. [↑](#footnote-ref-44)
45. Donner, A. and Klar, N. 2000. Design and Analysis of Cluster Randomization Trials in Health Research. Arnold. London. [↑](#footnote-ref-45)
46. Shadish, Cook TD, Cambell DT. Experimental and Quasi-Experimental Designs for Generalized Causal Inference. Boston: Houghton Mifflin. 2002. [↑](#footnote-ref-46)
47. Wagner AK, Soumerai SB, Zhang F, Ross-Degnan D. Segmented regression analysis of interrupted time series studies in medication use research. J Clin Pharm Ther. 2002 Aug; 27 (4):299-309. PMID: 12174032 [↑](#footnote-ref-47)
48. Reiss SK, Ross-Degnan D, Zhang F, Soumerai SB, Zaslavsky AM, Wharam JF. Effect of Switching to a High-Deductible Health Plan on Use of Chronic Medications. *Health Serv Res* Mar 17 2011. [↑](#footnote-ref-48)
49. Cook TD, Campbell, DT. Quasi-Experimentation: Design and Analysis Issues for Field Settings. Boston: Houghton Mifflin; 1979 [↑](#footnote-ref-49)
50. Slattery ML, Murtaugh MA, Quesenberry C, Caan BJ, Edwards S, Sweeney C. Changing population characteristics, effect-measure modification, and cancer risk factor identification. *Epidemiol Perspect Innov* 2007;4:10. [↑](#footnote-ref-50)
51. *SAS Institute Inc. SAS OnlineDoc®, Version 9. SAS Institute Inc.: Cary, NC 2002-2006.* [↑](#footnote-ref-51)
52. Allison PD, SAS Institute. Survival analysis using the SAS system : a practical guide. Cary, NC: SAS Institute; 1995. [↑](#footnote-ref-52)
53. Clayton DG. A Monte Carlo method for Bayesian inference in frailty models. *Biometrics* Jun 1991;47(2):467-485. [↑](#footnote-ref-53)
